# Manganese-catalyzed dehydroxyamination of catechols for the synthesis of redox-active and heterocyclic scaffolds

**DOI:** 10.1039/d5ra09855f

**Published:** 2026-05-05

**Authors:** Zahra Naseri Motlagh, Jasem Aboonajmi, Farhad Panahi, Laleh Khalvati, Hashem Sharghi

**Affiliations:** a Department of Chemistry, College of Sciences, Shiraz University Shiraz 71454 Iran Panahi@shirazu.ac.ir j.aboonajmi.ch@gmail.com +98 7132280926 +98 7136137136

## Abstract

A catalytic protocol employing manganese(ii) nitrate [Mn(NO_3_)_2_] has been developed for the selective C–O bond cleavage of catechols, enabling subsequent C–N bond formation. This transformation offers a versatile approach to the synthesis of 2-(arylamino)phenols, dihydrobenzo[*d*]oxazoles, and benzoxazoles under mild conditions. Aromatic amines such as anilines reacted smoothly to afford 2-(arylamino)phenols in excellent yields. In the presence of ketones, the methodology was further extended to the efficient synthesis of novel dihydrobenzo[*d*]oxazole derivatives. In contrast, the use of aliphatic amines directly led to benzoxazole products, with the corresponding 2-(alkylamino)phenol intermediates not being observed under the optimized conditions. This Mn(ii)-catalyzed strategy demonstrates broad substrate scope and high selectivity, offering a practical route to valuable N-heterocyclic scaffolds.

## Abstract 2

A catalytic protocol employing manganese(ii) nitrate [Mn(NO_3_)_2_] has been developed for the selective C–O bond cleavage of catechols, enabling subsequent C–N bond formation.

## Graphical abstract

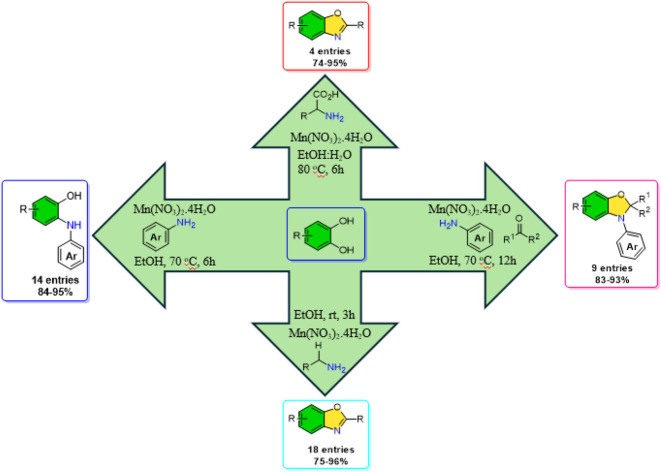

## Introduction

**Scheme 1:**
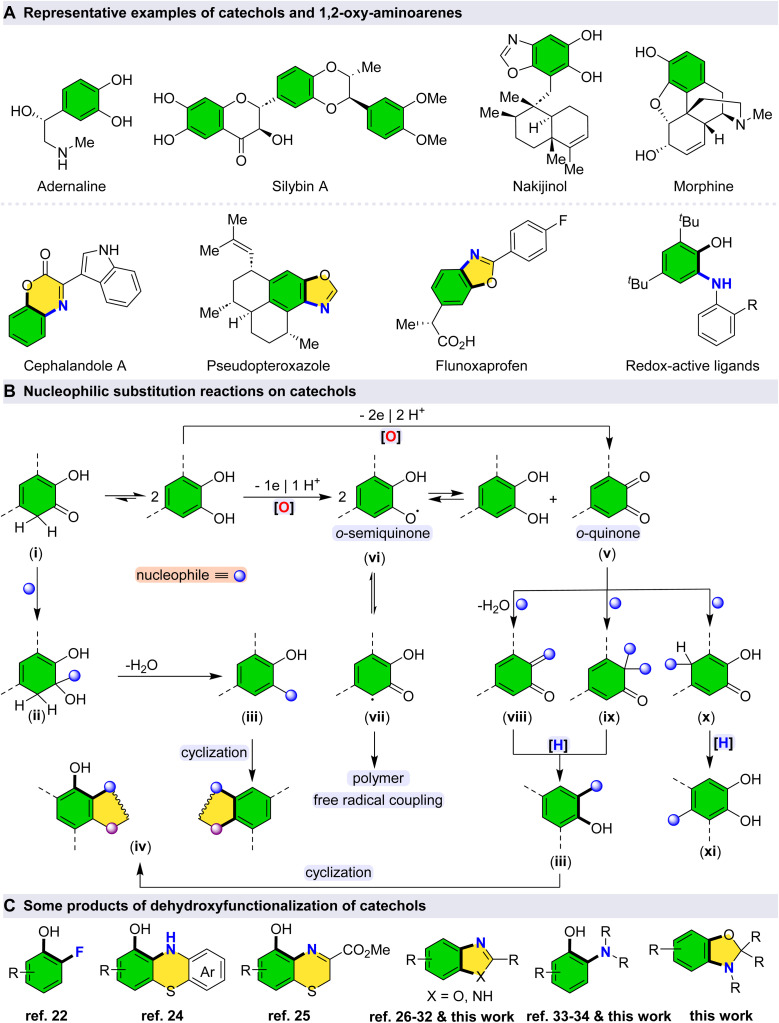
(A) Representative examples of catechols and their corresponding 1,2-oxy-aminoarene derivatives; (B) synthetic strategies for nucleophilic substitution reactions on catechols *via* dehydroxyfunctionalization; (C) selected products obtained from the dehydroxyfunctionalization of catechols.

Catechols represent an important class of naturally occurring organic compounds that exhibit a broad spectrum of biological activities and hold significant value as substrates in organic synthesis (Scheme 1A).^1–4^ The development of new methodologies for the derivatization of catechols not only facilitates the construction of novel organic molecules based on these privileged scaffolds but also enables late-stage functionalization of natural products, thereby enhancing their chemical diversity for potential biological and medicinal applications.^5,6^

Selective C–O bond cleavage in catechols can be achieved under mild catalytic conditions, providing a useful strategy for their functionalization, particularly *via* nucleophilic substitution reactions (Scheme 1B). The intrinsic reactivity of the C–O bond in catechols is influenced by tautomerization, which can be further activated in the presence of Lewis acid catalysts.^7^ However, to prevent undesirable C–C bond cleavage or degradation of the catechol core, the use of mild and selective catalysts is essential.^8^ Such catalysts allow for fine control over both reactivity and selectivity, offering a practical approach to the chemoselective transformation of catechol derivatives.^9^

The reactivity of catechols can, in part, be rationalized through keto–enol tautomerization, which facilitates the formation of dehydroxyfunctionalized and subsequently cyclized products in the presence of a nucleophile.^10^ Upon tautomerization to the keto form (i), the resulting carbonyl group becomes susceptible to nucleophilic attack (ii), leading—following a dehydration step—to the formation of dehydroxyfunctionalized intermediates (iii).^11^ These intermediates can subsequently undergo intramolecular cyclization to afford cyclic products (iv).^12,13^ Alternatively, dehydroxyfunctionalized products may also arise through oxidative pathways. Given the relatively high oxidation potential of catechols, such transformations can be promoted even by mild oxidants, including molecular oxygen from air.^10^ Consequently, oxidative dehydroxyfunctionalization has emerged as an efficient and environmentally benign strategy for the synthesis of structurally diverse cyclic and substituted compounds.^14,15^ In the presence of an appropriate oxidizing agent, *o*-quinone species (v) can be generated directly *via* a two-electron oxidation process.^16^ Meanwhile, *o*-semiquinone species (vi) can be generated *via* one-electron transfer processes.^17^

These highly reactive intermediates can undergo disproportionation to regenerate catechol and *o*-quinone species.^18^ Additionally, the *o*-semiquinone moiety is capable of polymerization through C–C bond formation, likely proceeding *via* a stabilized radical isomer (vii), with the extent of free radical coupling being subject to control under appropriate conditions.^19,20^ The carbonyl functionality of *o*-quinone is susceptible to nucleophilic attack, for example by amines, through a dehydration mechanism, leading—upon subsequent reduction—to dehydroxyfunctionalized products (viii, iii).^21^ In the presence of fluoride sources, this transformation may proceed *via* an alternative pathway involving the formation of a difluoroketone intermediate. Thus, the oxidative deoxyfluorination of catechols has been successfully achieved by sequential oxidation, fluoride addition, and reduction steps (ix, iii).^22^

It is important to note that *o*-quinone, as an *α*,*β*-unsaturated ketone, can undergo conjugate (Michael-type) nucleophilic addition reactions, affording a variety of substituted products following reduction (x, xi).^23^ Nucleophilic substitution on catechols *via* selective C–O bond cleavage (*i.e.*, dehydroxyfunctionalization) presents a versatile approach for bond formation, enabling the diversification of catechol scaffolds and the generation of novel building blocks. Indeed, oxidative dehydroxyfunctionalization of catechols constitutes an efficient strategy for the synthesis of cyclic and substituted derivatives (Scheme 1C).^22,24–34^

**Scheme 2:**
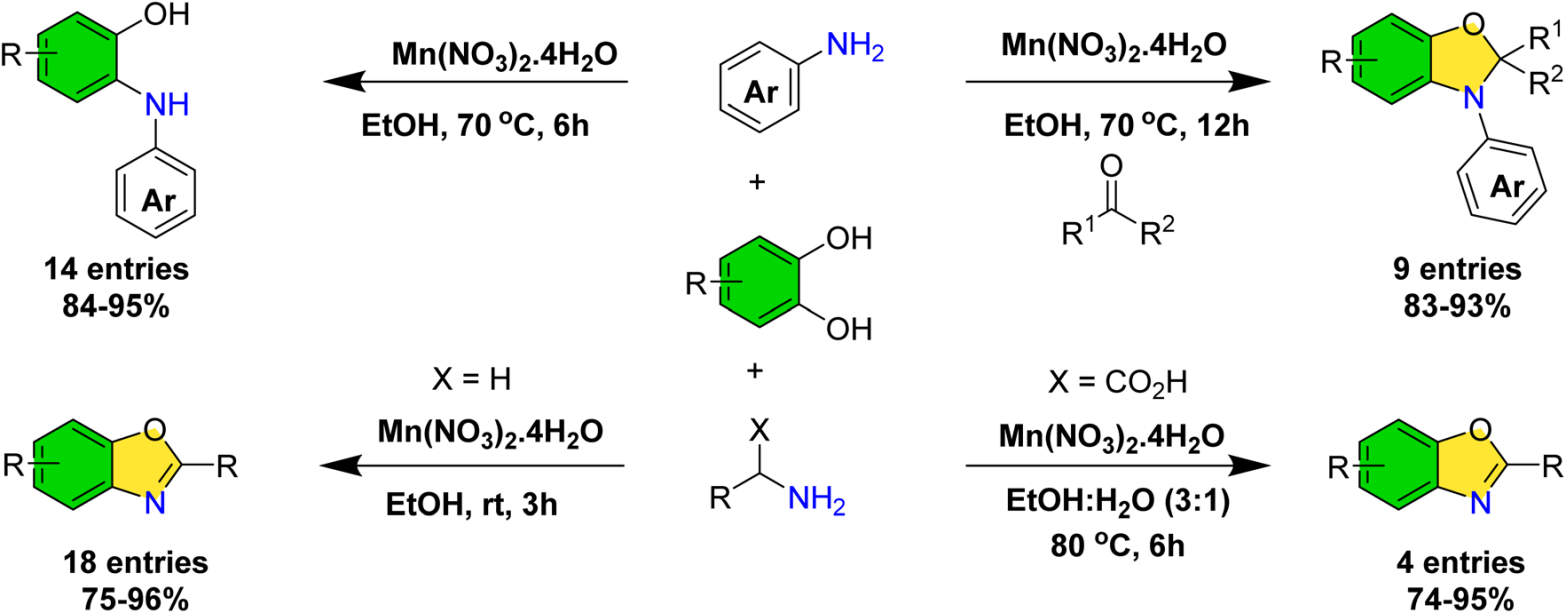
Synthesis of 2-(arylamino)phenols, dihydrobenzo[*d*]oxazoles and benzoxazoles manganese-catalyzed dehydroxyfunctionalization of catechols.

Building upon our ongoing research on the application of catechols in organic synthesis^35–39^ and recognizing the significance of 2-(arylamino)phenols as valuable redox-active ligands^40–42^—whose synthetic accessibility remains limited^43,44^—we sought to develop an efficient catalytic methodology for their preparation. During the course of this investigation, we discovered that dihydrobenzo[*d*]oxazoles can be synthesized *via* a multicomponent reaction involving catechols, anilines, and ketones. Furthermore, employing aliphatic amines under mild, room temperature conditions allowed for the isolation of the corresponding benzoxazole derivatives. Notably, subtle modifications in the reaction parameters enabled the successful synthesis of benzoxazole derivatives derived from amino acids (Scheme 2).

## Results and discussion

To identify optimal conditions for the synthesis of 2-(arylamino)phenol derivatives, a series of experiments was conducted using the model reaction between 3,5-di-*tert*-butylbenzene-1,2-diol (1) and 4-methylaniline (2a) under various reaction parameters (Table 1).

**Table 1:** Optimization of the reaction conditions for the synthesis of 2-(arylamino)phenol derivatives^a^

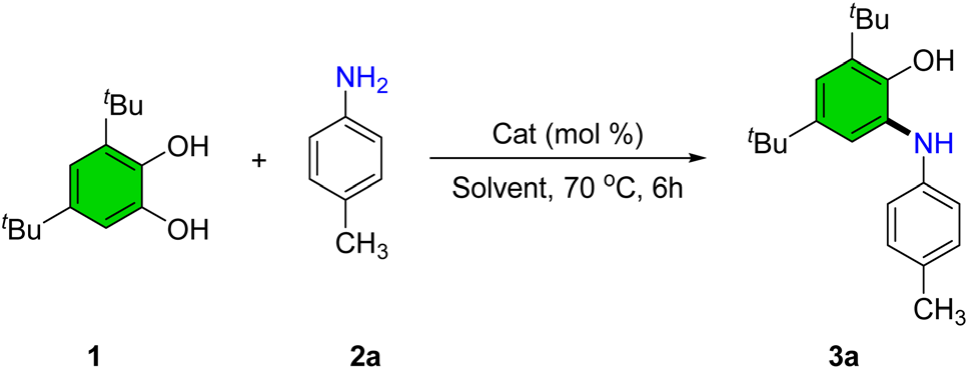			
Entry	Cat (mol%)	Solvent	Yield^b^ (%)
1	—	EtOH	Trace
2	Cr(NO_3_)_3_·9H_2_O (5 mol%)	EtOH	35
3	Bi(NO_3_)_3_·6H_2_O (5 mol%)	EtOH	18
4	Cu(NO_3_)_2_·3H_2_O (5 mol%)	EtOH	57
5	Cd(NO_3_)_2_·4H_2_O (5 mol%)	EtOH	65
6	Zn(NO_3_)_2_·6H_2_O (5 mol%)	EtOH	70
7	La(NO_3_)_3_·6H_2_O (5 mol%)	EtOH	88
8	**Mn(NO**_**3**_ **)**_**2**_ **·4H**_**2**_ **O (5 mol%)**	**EtOH**	**95**
9	MnCl_2_	EtOH	75
10	Mn(NO_3_)_2_·4H_2_O (5 mol%)	THF	18
11	Mn(NO_3_)_2_·4H_2_O (5 mol%)	Dioxane	5
12	Mn(NO_3_)_2_·4H_2_O (5 mol%)	CH_3_CN	50
13	Mn(NO_3_)_2_·4H_2_O (5 mol%)	MeOH	87
14	Mn(NO_3_)_2_·4H_2_O (5 mol%)	DCE	20
15	Mn(NO_3_)_2_·4H_2_O (5 mol%)	Isopropanol	55
16	Mn(NO_3_)_2_·4H_2_O (5 mol%)	—	Trace
17	Mn(NO_3_)_2_·4H_2_O (10 mol%)	EtOH	94
18	Mn(NO_3_)_2_·4H_2_O (2 mol%)	EtOH	62
19^c^	Mn(NO_3_)_2_·4H_2_O (5 mol%)	EtOH	20
20^d^	Mn(NO_3_)_2_·4H_2_O (5 mol%)	EtOH	72

a Reaction conditions: 3,5-di-*tert*-butylbenzene-1,2-diol (1.0 mmol), 4-methylaniline (1.0 mmol), solvent (5.0 mL), 6 h.

b Yield of isolated product.

c 25 °C.

d 50 °C.

In the absence of a catalyst and with refluxing ethanol, only trace amounts of product were observed (Table 1, entry 1). To improve the yield, several Lewis acid catalysts were screened. Cr(NO_3_)_3_·9H_2_O (5 mol%) afforded 35% product yield (Table 1, entry 2). Subsequently, nitrate salts of various metals, including Bi, Cu, Cd, Zn, La, and Mn were evaluated (Table 1, entries 3–8). Among these, Mn(NO_3_)_2_·4H_2_O exhibited the highest catalytic activity, providing the desired product in 95% yield, and was thus selected as the catalyst of choice. An alternative manganese source, MnCl_2_, afforded a moderate yield of 75% (Table 1, entry 9).

A range of solvents-including tetrahydrofuran, dioxane, acetonitrile, methanol, dichloroethane, and isopropanol-were tested; however, none improved upon the reaction performance observed in ethanol (Table 1, entries 10–15). Solvent-free conditions yielded only a trace product (Table 1, entry 16). Increasing catalyst loading beyond 5 mol% did not enhance conversion, whereas reducing it to 2 mol% led to a significant drop in yield to 62% (Table 1, entries 17 and 18). Lowering the reaction temperature also adversely affected product formation, with only 72% yield at 50 °C and substantially reduced conversion at ambient temperature (Table 1, entries 19 and 20).

**Scheme 3:**
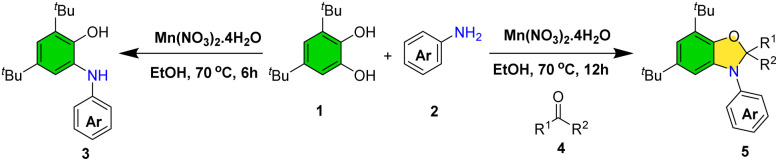
Synthesis of 2-(arylamino)phenols without acetone and dihydrobenzo[*d*]oxazole derivatives in the presence of acetone.

During optimization, the use of acetone as solvent revealed its participation in the reaction, yielding 2,3-dihydrobenzo[*d*]oxazole (5a) instead of the expected 2-(arylamino)phenol. Consequently, reaction conditions were further optimized for this product class (see SI, Table S1). Thus, conditions for the selective synthesis of both 2-(arylamino)phenols and dihydrobenzo[*d*]oxazoles were established (Scheme 3).

**Scheme 4:**
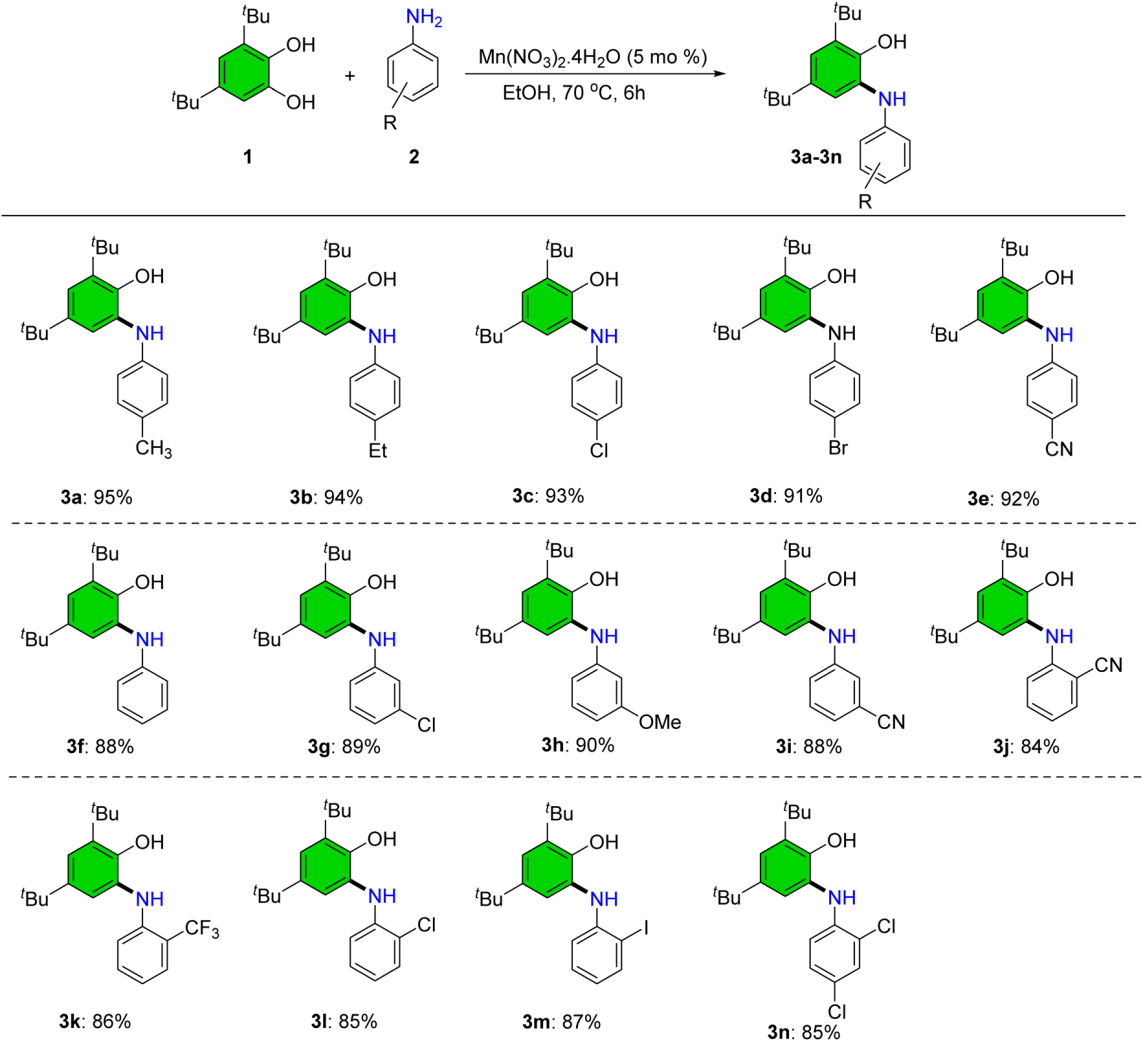
Synthesis of 2-(arylamino)phenol derivatives. Reaction condition: 1 (1.0 mmol), 2 (1.0 mmol), Mn(NO_3_)_2_·4H_2_O (5 mol%), EtOH (5.0 mL), and 70 °C. Yields corresponds to the isolated products.

Applying the optimized parameters, a variety of 2-(arylamino)phenol derivatives were synthesized from 3,5-di-*tert*-butylbenzene-1,2-diol and substituted anilines (Scheme 4). Anilines bearing electron-donating groups (–Me, –Et, –OMe) at *meta* and *para* positions afforded the corresponding products (3a, 3b, 3h) in excellent yields (>90%). An electron-withdrawing substituent (–CF_3_) was also well tolerated at the *ortho* position, furnishing product 3k in 86% yield.

The electron-withdrawing cyano group (–CN) was compatible at the *ortho*, *meta*, and *para* positions, delivering the corresponding products (3e, 3i, 3j) in excellent yield. Halogen-substituted anilines similarly yielded the expected products in high yields (3c, 3d, 3g, 3l–3n). Sterically hindered anilines such as 3k and 3n were well tolerated under these conditions (Scheme 4).

**Scheme 5:**
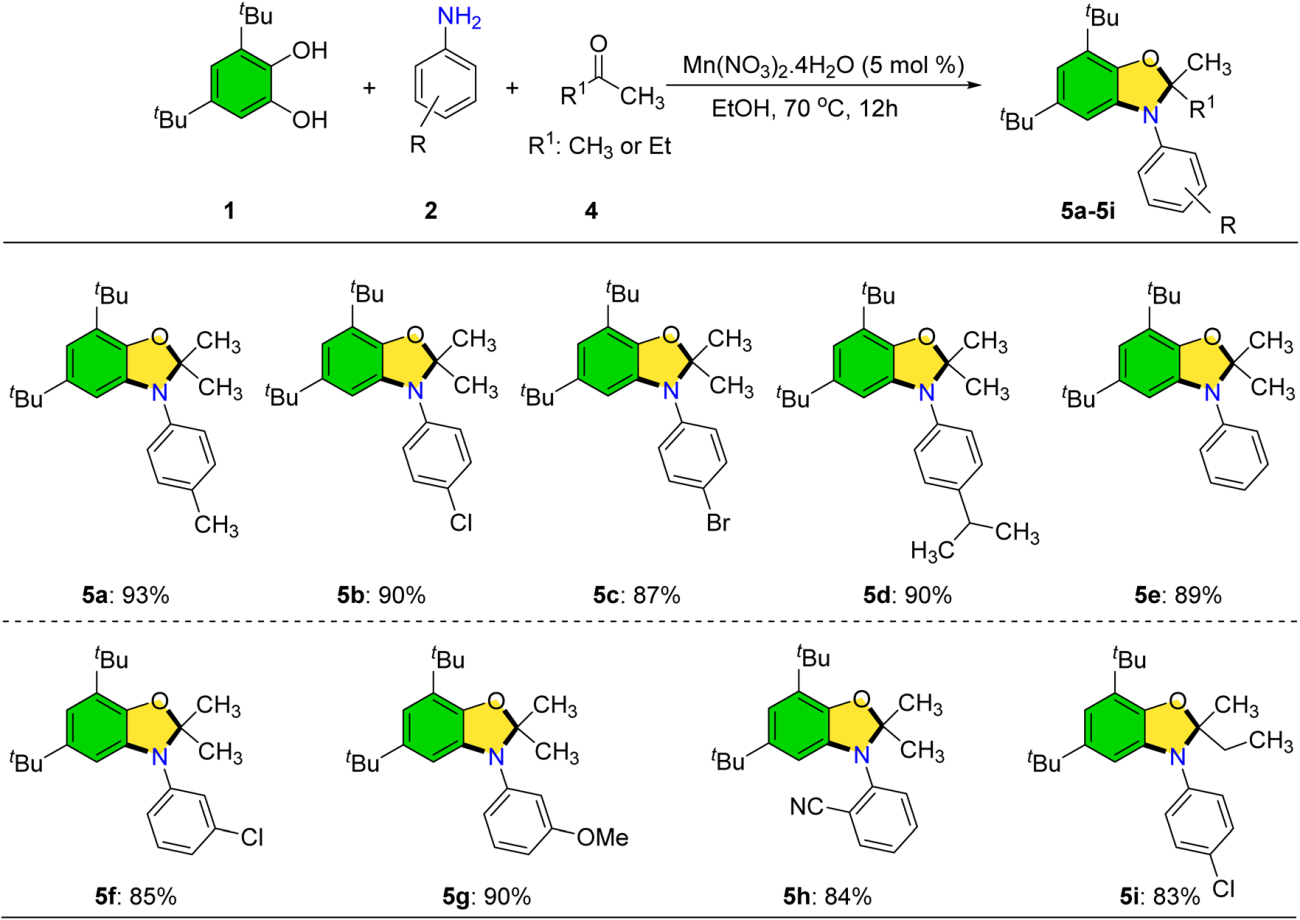
Synthesis of dihydrobenzo[*d*]oxazoles. Reaction conditions: 1 (1.0 mmol), 2 (1.0 mmol), 4 (5.0 mL), Mn(NO_3_)_2_·4H_2_O (5 mol%), EtOH (5.0 mL), and 70 °C. Yields correspond to isolated products.

We have investigated the reactivity of biologically relevant catechols such as DOPAC, dopamine, and l-DOPA under our optimized reaction conditions for 24 h. Unfortunately, these substrates did not provide the desired products (SI, Scheme S1A–C). We conducted a model reaction between 3,5-di-*tert*-butylbenzene-1,2-diol and indole under our standard optimized conditions for 24 h. However, no desired product was observed (SI, Scheme S1D). Following the successful synthesis of 2-(arylamino)phenols, attention was turned to the preparation of dihydrobenzo[*d*]oxazoles *via* a three-component reaction involving catechols, anilines, and acetone catalyzed by Mn(NO_3_)_2_·4H_2_O (Scheme 5).

Anilines bearing both electron-donating and electron-withdrawing groups at various positions underwent smooth transformation to the target oxazoles in high yields. Moreover, ethyl methyl ketone was found to be a viable ketone partner, delivering product 5i in 83% yield; this compound contains a stereogenic center. However, bulkier ketones such as acetophenone and benzophenone failed to afford cyclized products, instead yielding only the corresponding 2-(arylamino)phenols.

In order to evaluate the scope of our method, we tested cyclic ketones such as cyclopentanone and cyclohexanone under the optimized reaction conditions for 24 h. However, no desired product was obtained in these cases.

**Scheme 6:**
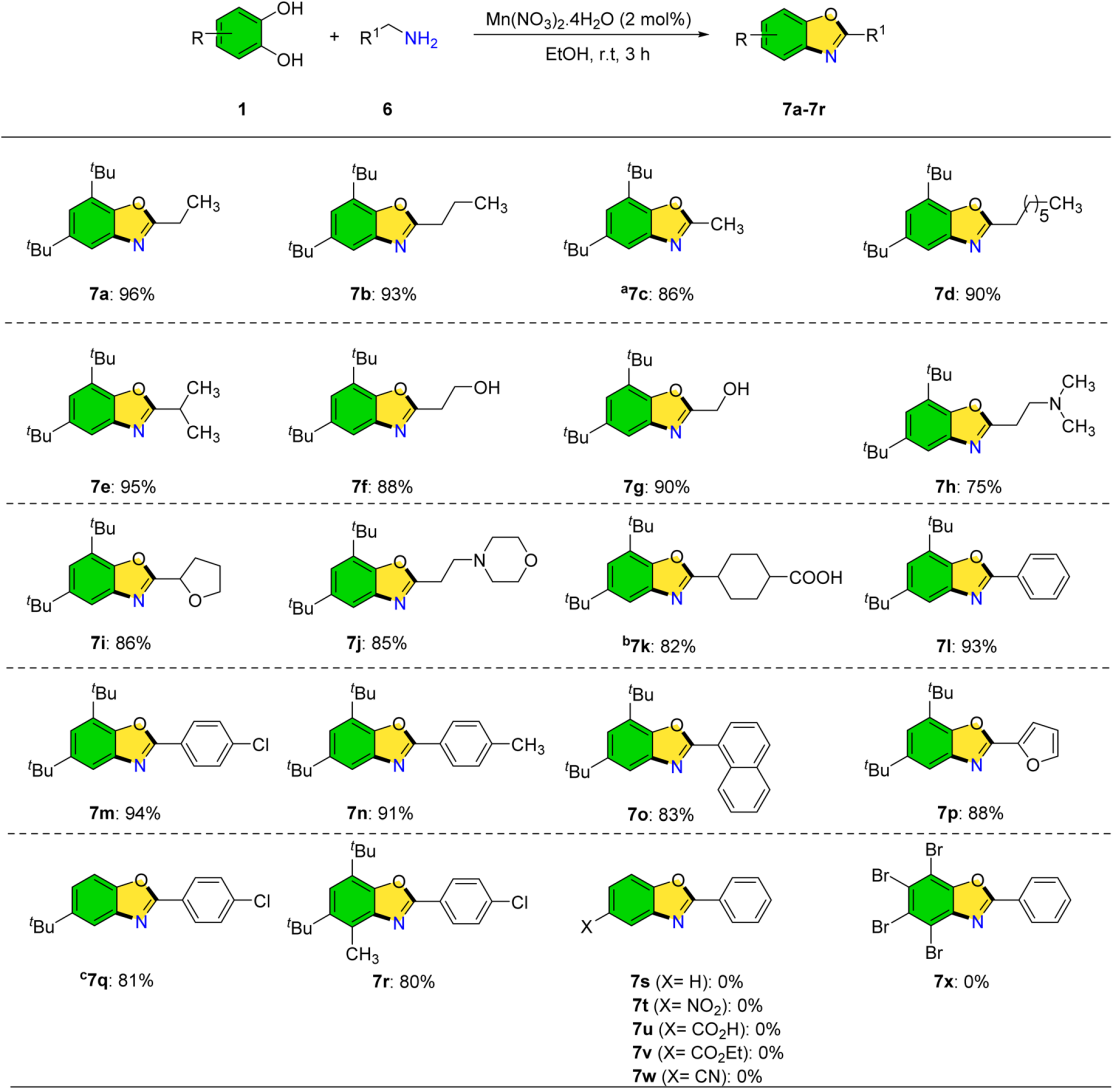
Synthesis of benzoxazoles *via* primary amines. Reaction condition: 1 (1.0 mmol), 6 (1.0 mmol), Mn(NO_3_)_2_·4H_2_O (2 mol%), and EtOH (5.0 mL). Yields correspond to the isolated product. ^*a*^70% aqueous solution of ethylamine. ^*b*,*c*^6 h.

The reaction of catechol with primary amines was also investigated to synthesize 2-(alkylamino)phenols. Under optimized conditions, however, benzoxazole products were predominantly formed at room temperature in the presence of catalytic manganese. This approach offers a mild, efficient route to benzoxazoles from abundant starting materials (Scheme 6). Various primary amines including propylamine, butylamine, ethylamine, and octylamine were compatible, furnishing the corresponding benzoxazoles (7a–7d) in good to excellent yields. Branched aliphatic amines such as isobutylamine (7e) and amino alcohols like propanolamine and ethanolamine (7f, 7g) also participated efficiently. *N*,*N*-Dimethyl-1,3-propanediamine yielded a benzoxazole product in 75% yield. Heterocyclic primary amines bearing morpholine and tetrahydrofuran moieties afforded bis-heterocyclic derivatives (7i, 7j). Notably, benzoxazole derivative 7k, known for its biological activity against severe pain and bleeding, was synthesized using tranexamic acid as the amine precursor. Compounds 7l–7n were created with good to excellent yields after testing the reaction of benzylamines with electron-donating and electron-withdrawing substituents (–Cl, and –CH_3_) with 3,5-di-*tert*-butylbenzene-1,2-diol to increase the number of benzoxazole derivatives. The synthesis of benzoxazole derivatives was achieved using naphthalen-2-ylmethanamine and provided the corresponding products in good yields. Although the naphthyl group introduces significant steric hindrance, the reaction proceeded smoothly (7o). The use of furfurylamine led to the successful formation of the desired benzoxazole derivatives in good yields (7p).

The reaction was also conducted using different catechol derivatives, such as 4,6-di-*tert*-butyl-3-methylbenzene-1,2-diol, and 4-(*tert*-butyl)benzene-1,2-diol in order to increase the diversity of synthesized products. These reactions produced significant outcomes as well as good product yields 7q–7r. The reactions of benzylamine with catechol, 4-nitrobenzene-1,2-diol, 3,4-dihydroxybenzoic acid, ethyl 3,4-dihydroxybenzoate, 3,4-dihydroxybenzonitrile, and 3,4,5,6-tetrabromobenzene-1,2-diol were investigated over 24 h; however, no desired products were obtained (7s–7x).

**Scheme 7:**
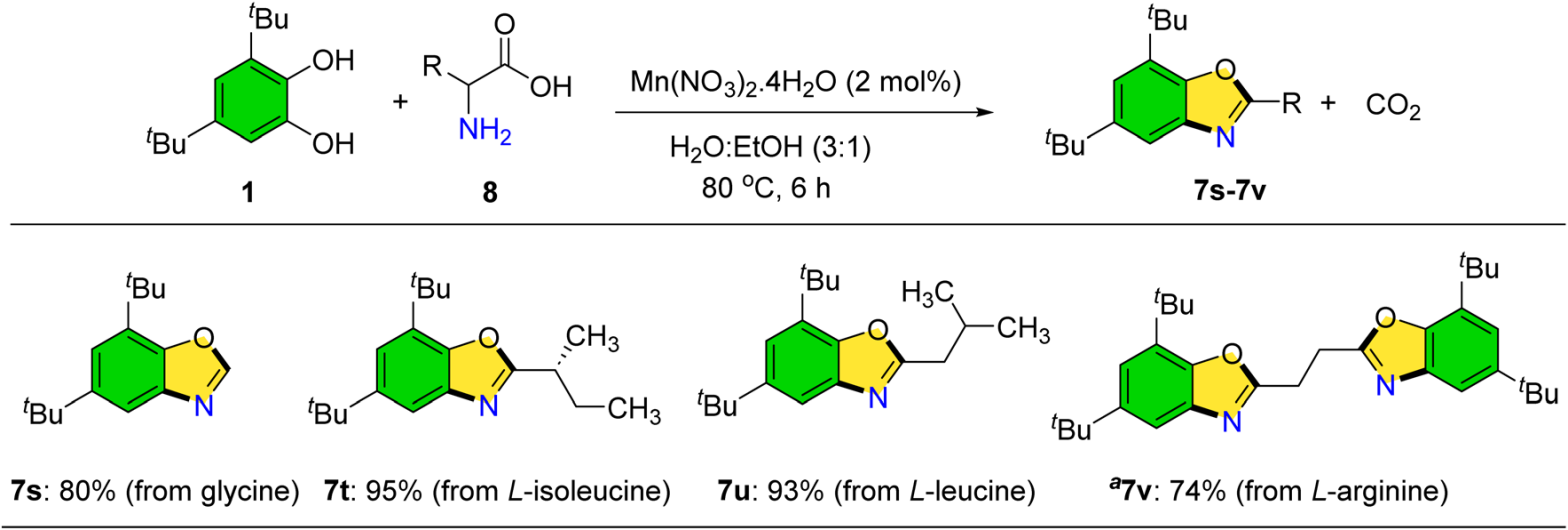
Synthesis of benzoxazoles through amino acids. General condition: 1 (1.0 mmol), 8 (1.0 mmol), Mn(NO_3_)_2_·4H_2_O (2 mol%), and H_2_O : EtOH (3 : 1). Isolated yield. ^*a*^2 mmol of 3,5-di-*tert*-butylbenzene-1,2-diol.

When amino acids were employed as primary amines, carbon dioxide release was observed, and benzoxazole products were isolated in high yields (Scheme 7).^32^ Optimization of this transformation established that a water–ethanol solvent mixture (3 : 1) under 80 °C improved product yields. Benzoxazole derivatives incorporating glycine, l-isoleucine, l-leucine, and l-arginine were successfully prepared (7s–7v).

**Scheme 8:**
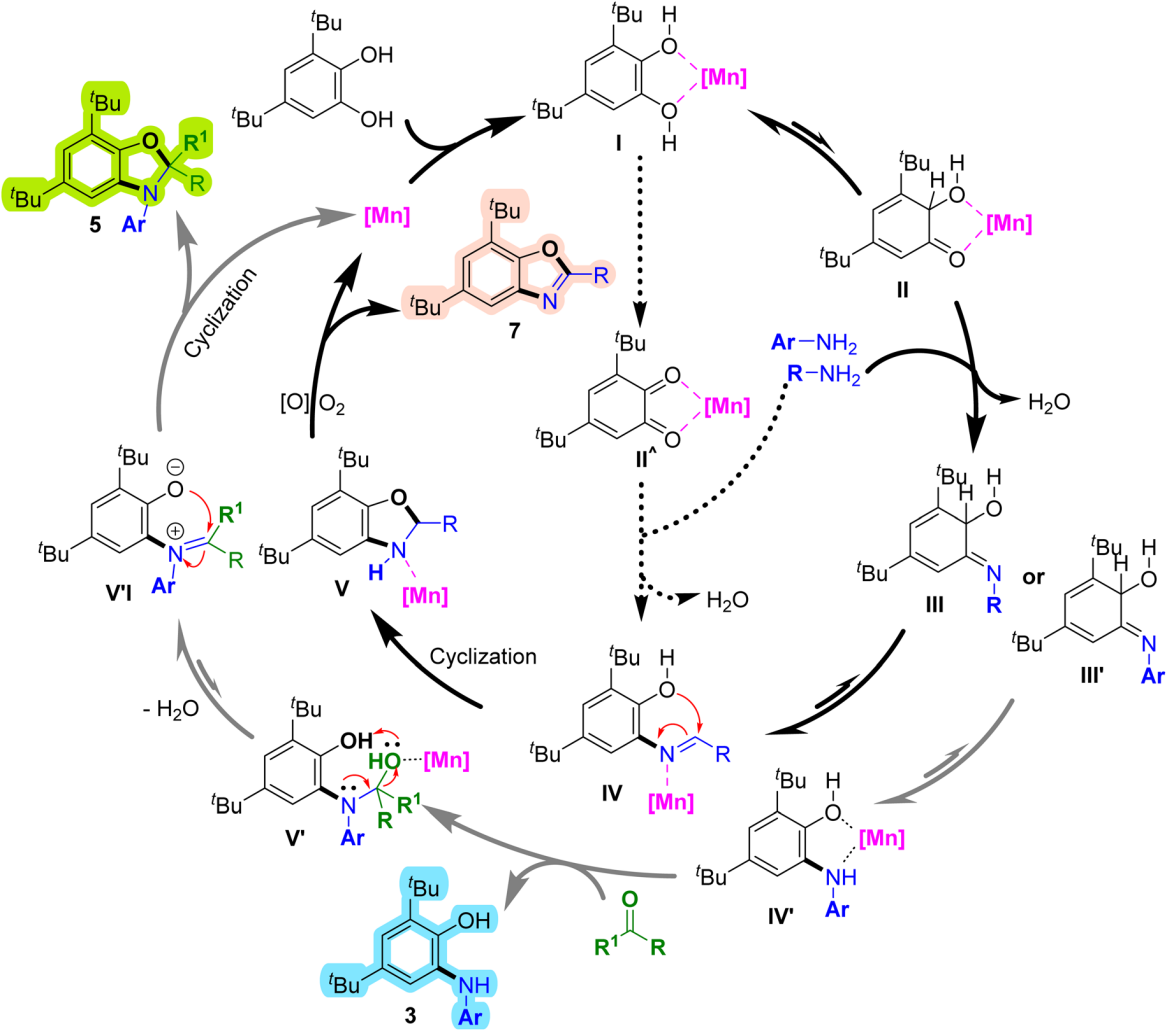
Proposed mechanism for the synthesis of 2-(arylamino)phenols, dihydrobenzo[*d*]oxazoles and benzoxazoles. Mn is assumed to maintain an oxidation state of +2 during the reaction cycle.

Based on the proposed catalytic cycle (Scheme 8), manganese coordinates with 3,5-di-*tert*-butylbenzene-1,2-diol to form the initial intermediate (I).^45^ Subsequent enol–keto tautomerization generates a semiquinone intermediate (II).^30,35,46^ Nucleophilic attack by the aliphatic amine forms an imine intermediate (III), which tautomerizes to a Schiff base (IV).^26,29^ Cyclization of (IV) affords the benzoxazoline intermediate (V).^30,32,35,47^ Finally, aerobic oxidative dehydrogenation converts (V) into the benzoxazole product (7).^35,47,48^ Alternatively, condensation of *ortho*-quinone (II′) with the amine can lead to a similar intermediate (IV), followed by cyclization, aromatization, and hydrogen elimination. When aromatic amines are used instead of aliphatic amines, the imine intermediate (III′) is formed, which tautomerizes to 2-(arylamino)phenol (3). Subsequent nucleophilic attack of the amino group on the ketone yields intermediate (V′), which dehydrates and cyclizes to give the dihydrobenzo[*d*]oxazole product (5).

## Experimental section

All chemicals used in the reactions were purchased from Merck, Sigma-Aldrich, and Fluka. The progress of the proposed reactions was followed by thin-layer chromatography (TLC) using silica gel 254 UV/S plates, and all products were analyzed by spectral data after purification. Infrared (IR) spectra were recorded on a Shimadzu FT-IR 8300 spectrophotometer. ^1^H NMR and ^13^C NMR spectra were performed by Bruker Avance DPX-300 and Bruker Avance DPX-400 instruments, and tetramethylsilane (TMS) was used as the internal standard. The *J* and *δ* values in all spectra are in Hz and ppm, and the singlet, doublet, triplet, quaternary, and multiplet spectral line splittings are indicated by the symbols s, d, t, q, and m, respectively.

### General procedure for the synthesis of 2-(arylamino)phenol derivatives 3a–3n from aromatic amines

In a 10 mL round bottom flask, a mixture of manganese nitrate tetrahydrate (5 mol%) with 3,5-di-*tert*-butylbenzene-1,2-diol (1.0 mmol), aniline derivatives (1.0 mmol) in ethanol solvent (5 mL) was stirred at 70 °C for 6 h. The progress of the reaction was monitored by TLC. After extraction with chloroform (5 mL) and water (10 mL), the crude products are purified by column chromatography with ethyl acetate/petroleum ether solvent.

### General procedure for the synthesis of dihydrobenzo[*d*]oxazoles 5a–5i*via* aromatic amines and acetone

In a 50 mL round bottom flask equipped with a magnetic stirrer, a mixture of manganese nitrate tetrahydrate catalyst (5 mol%), 3,5-di-*tert*-butylbenzene-1,2-diol (1.0 mmol), aniline derivatives (1.0 mmol), acetone (5 mL), and ethanol solvent (5 mL) at 70 °C was stirred, and the progress of the reaction was monitored by TLC. After 12 h, the solvent was evaporated under reduced pressure. The residual material was extracted with chloroform (10 mL) and water (20 mL) three times. The corresponding organic phase was separated and purified using column chromatography and ethyl acetate/petroleum ether solvents.

### General synthesis of benzoxazole derivatives 7a–7r using aliphatic amines

In a 10 mL round-bottom flask equipped with a magnetic stirrer, 3,5-di-*tert*-butylbenzene-1,2-diol (1.0 mmol), aliphatic amine (1.0 mmol), and manganese nitrate tetrahydrate (2 mol%) were dissolved in ethanol (2 mL) at room temperature. After 3 h of monitoring the reaction progress using TLC, the crude benzoxazole product was synthesized. After extracting the obtained material with chloroform (10 mL) and water (20 mL), it was purified by column chromatography with ethyl acetate/petroleum ether solvent to purify the synthetic product.

### General synthesis of benzoxazole compounds 7s–7v through amino acids

In a 25 mL round-bottom flask, a mixture of manganese nitrate tetrahydrate (2 mol%), 3,5-di-*tert*-butylbenzene-1,2-diol (1.0 mmol), and amino acid (1.0 mmol) in 4 mL of ethanol and water (3 : 1) was stirred at 80 °C for 6 h. The resulting material was then cooled to room temperature, and after extraction with chloroform (10 mL) and water (20 mL), purified by column chromatography with ethyl acetate/petroleum ether.

## Conclusions

In summary, we have developed a versatile and efficient manganese-catalyzed method for the synthesis of 2-(arylamino)phenol derivatives, a significant class of redox-active ligands. The reaction of catechols with arylamines in the presence of catalytic Mn(NO_3_)_2_ proceeded under mild conditions and furnished the desired products in excellent yields. Furthermore, the incorporation of ketones into the reaction enabled the synthesis of dihydrobenzo[*d*]oxazole derivatives. Under optimized conditions, aliphatic amines underwent smooth transformation with catechols to afford benzoxazole products in good to high yields. Notably, amino acids were also successfully employed as amine sources, providing access to structurally diverse benzoxazoles *via* a decarboxylative pathway. This methodology offers a practical and modular approach to the synthesis of nitrogen- and oxygen-containing heterocycles from readily available starting materials.

## Author contributions

J. A., F. P., and H. S. conceived and designed the study. J. A. and Z. N. M. carried out the experimental work. L. K. contributed to the synthesis of selected derivatives. J. A. and F. P. prepared the initial draft of the manuscript. All authors contributed to data analysis and interpretation and reviewed and approved the final version of the manuscript.

## Conflicts of interest

There are no conflicts to declare.

## Supplementary Material

RA-016-D5RA09855F-s001

## Data Availability

All data generated or analyzed during this study are included in this published article and its supplementary information (SI). Supplementary information: experimental section, spectral data and copy of NMRs. See DOI: https://doi.org/10.1039/d5ra09855f.
