# Supplementary material for: Manganese-catalyzed dehydroxyamination of catechols for the synthesis of redox-active and heterocyclic scaffolds
Source: RSC Adv. 2026 May 5;16(26):23326–34. doi: 10.1039/d5ra09855f (PMC13140292; doi:10.1039/d5ra09855f)
Supplement: RA-016-D5RA09855F-s001 [file RA-016-D5RA09855F-s001.pdf]

Supplementary Information (SI) for RSC Advances.
This journal is © The Royal Society of Chemistry 2026

Manganese-Catalyzed Dehydroxyamination of Catechols for the Synthesis of Redox-Active and Heterocyclic Scaffolds

Zahra Naseri Motlagh,[a,T] Jasem Aboonajmi,[a,T]* Farhad Panahi,[a,]* Laleh Khalvati[a] and Hashem Sharghi[a]

[a] Department of Chemistry, College of Sciences, Shiraz University, Shiraz 71454, Iran

[T] These authors contributed equally to this work.

Table of Contents	
**Contents**	**Page**
Experimental Section	S2
General procedure for the synthesis of 2-(arylamino)phenol derivatives **3a-3n** from aromatic amines	S2
General procedure for the synthesis of dihydrobenzo[*d*]oxazoles **5a-5i** via aromatic amines and acetone	S2
General synthesis of benzoxazole derivatives **7a-7r** using aliphatic amines	S2
General synthesis of benzoxazole compounds **7s-7v** through amino acids	S2
Table S1. Optimization of the reaction conditions for the synthesis of dihydrobenzo[*d*]oxazoles	S3
Table S2. Investigating of nitrate salts for the synthesis of benzoxazole via primary amine in the presence of various solvents	S4
Scheme S1. The reaction of amine (1.0 mmol) and catechol derivatives (1.0 mmol) in the presence of $Mn(NO_3)_2$ (5 mol%) in EtOH (5 mL) at 70 °C.	S5
Product characterization data	S6-S19
References	S20
Copy of NMR spectra of 2-(arylamino)phenols, dihydrobenzo[*d*]oxazoles, and benzoxazoles	S21-S65

Experimental Section

All chemicals used in the reactions were purchased from Merck, Sigma Aldrich, and Fluka. The progress of the proposed reactions was followed by thin-layer chromatography (TLC) using silica gel 254 UV/S plates, and all products were analyzed by spectral data after purification. Infrared (IR) spectra were recorded on a Shimadzu FT-IR 8300 spectrophotometer. ^{1}H NMR and ^{13}C NMR spectra were performed by Bruker Avance DPX-300 and Bruker Avance DPX-400 instruments, and tetramethylsilane (TMS) was used as the internal standard. The J and δ values in all spectra are in Hz and ppm, and the singlet, doublet, triplet, quaternary, and multiplet spectral line splittings are indicated by the symbols s, d, t, q, and m, respectively.

General procedure for the synthesis of 2-(arylamino)phenol derivatives 3a-3n from aromatic amines

In a 10 mL round bottom flask, a mixture of manganese nitrate tetrahydrate (5 mol%) with 3,5-di-*tert*-butylbenzene-1,2-diol (1.0 mmol), aniline derivatives (1.0 mmol) in ethanol solvent (5 mL) was stirred at 70 ^{o}C for 6 h. The progress of the reaction was monitored by thin-layer chromatography (TLC). After extraction with chloroform (5 ml) and water (10 ml), the crude products are purified by column chromatography with ethyl acetate/petroleum ether solvent.

General procedure for the synthesis of dihydrobenzo[*d*]oxazoles 5a-5i via aromatic amines and acetone

In a 50 mL round bottom flask equipped with a magnetic stirrer, a mixture of manganese nitrate tetrahydrate catalyst (5 mol%), 3,5-di-*tert*-butylbenzene-1,2-diol (1.0 mmol), aniline derivatives (1.0 mmol), acetone (5 mL), and ethanol solvent (5 mL) at 70 ^{o}C was stirred, and the progress of the reaction was monitored by thin-layer chromatography (TLC). After 12 h, the solvent was evaporated under reduced pressure. The residual material was extracted with chloroform (10 mL) and water (20 mL) three times. The corresponding organic phase was separated and purified using column chromatography and ethyl acetate/petroleum ether solvents.

General synthesis of benzoxazole derivatives 7a-7r using aliphatic amines

In a 10 mL round-bottom flask equipped with a magnetic stirrer, 3,5-di-*tert*-butylbenzene-1,2-diol (1.0 mmol), aliphatic amine (1.0 mmol), and manganese nitrate tetrahydrate (2 mol%) were dissolved in ethanol (2 mL) at room temperature. After 3h of monitoring the reaction progress using thin-layer chromatography (TLC), the crude benzoxazole product was synthesized. After extracting the obtained material with chloroform (10 mL) and water (20 mL), it was purified by column chromatography with ethyl acetate/petroleum ether solvent to purify the synthetic product.

General synthesis of benzoxazole compounds 7s-7v through amino acids

In a 25 mL round-bottom flask, a mixture of manganese nitrate tetrahydrate (2 mol%), 3,5-di-*tert*-butylbenzene-1,2-diol (1.0 mmol), and amino acid (1.0 mmol) in 4 mL of ethanol and water (3:1) was stirred at 80 ^{o}C for 6 h. The resulting material was then cooled to room temperature, and after extraction with chloroform (10 mL) and water (20 mL), purified by column chromatography with ethyl acetate/petroleum ether.

Table S1. Optimization of the reaction conditions for the synthesis of dihydrobenzo[*d*]oxazoles.[a]

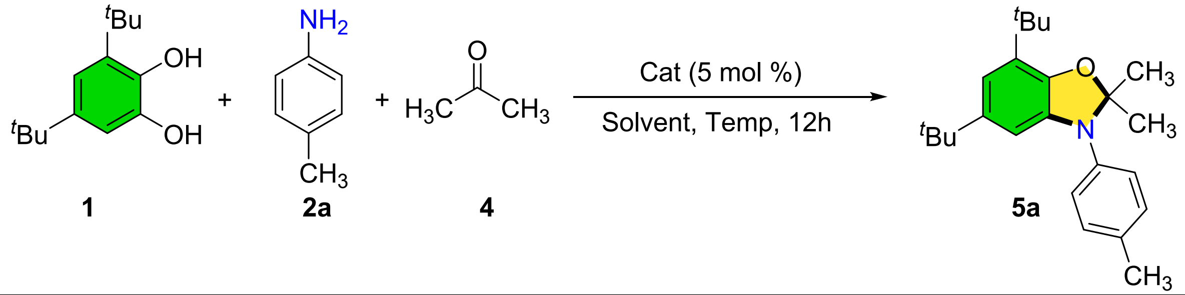

Entry	Cat (mol %)	Solvent	Temp (°C)	Yield (%)[b]
1	-	EtOH	70	-
**2**	**$Mn(NO_3)_2.4H_2O$ (5 mol%)**	**EtOH**	**70**	**93**
3	$Bi(NO_3)_3.6H_2O$ (5 mol%)	EtOH	70	10
4	$Cd(NO_3)_2.4H_2O$ (5 mol%)	EtOH	70	50
5	$La(NO_3)_3.6H_2O$ (5 mol%)	EtOH	70	85
6	$AgNO_3$ (5 mol%)	EtOH	70	20
7	$Cu(NO_3)_2.3H_2O$ (5 mol%)	EtOH	70	40
8	$Ce(NO_3)_3.6H_2O$ (5 mol%)	EtOH	70	45
9	$Cr(NO_3)_3.9H_2O$ (5 mol%)	EtOH	70	15
10	$Zn(NO_3)_2.6H_2O$ (5 mol%)	EtOH	70	62
11	$Mg(NO_3)_2.6H_2O$ (5 mol%)	EtOH	70	5
12	$Mn(NO_3)_2.4H_2O$ (5 mol%)	THF	70	30
13	$Mn(NO_3)_2.4H_2O$ (5 mol%)	Isopropanol	70	80
14	$Mn(NO_3)_2.4H_2O$ (5 mol%)	CH_3CN	70	25
15	$Mn(NO_3)_2.4H_2O$ (5 mol%)	DCE	70	40
16	$Mn(NO_3)_2.4H_2O$ (5 mol%)	Dioxane	70	30
17	$Mn(NO_3)_2.4H_2O$ (5 mol%)	MeOH	70	55
18	$Mn(NO_3)_2.4H_2O$ (5 mol%)	-	70	45
19	$Mn(NO_3)_2.4H_2O$ (10 mol%)	EtOH	70	85
20	$Mn(NO_3)_2.4H_2O$ (2 mol%)	EtOH	70	80
21	$Mn(NO_3)_2.4H_2O$ (5 mol%)	EtOH	25	10
22	$Mn(NO_3)_2.4H_2O$ (5 mol%)	EtOH	50	55
[c]23	$Mn(NO_3)_2.4H_2O$ (5 mol%)	EtOH	70	60
[d]24	$Mn(NO_3)_2.4H_2O$ (5 mol%)	EtOH	70	42
[e]25	$Mn(NO_3)_2.4H_2O$ (5 mol%)	EtOH	70	-

[a]Reaction conditions: 3,5-di-*tert*-butylbenzene-1,2-diol (1.0 mmol), 4-methylaniline (1.0 mmol), acetone (5.0 mL), solvent (5.0 mL), 12 h. [b]Yield of isolated product. [c]EtOH (2.0 mL). [d]Acetone (2.0 mL). [e]Acetone (1.0 mmol).

Table S2. Investigating of nitrate salts for the synthesis of benzoxazole via primary amine in the presence of various solvents.[a]

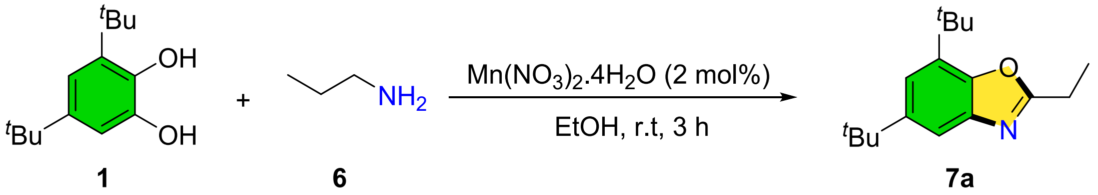

Entry	Cat (mol %)	Solvent	Yield (%)[b]
1	-	EtOH	-
**2**	**$Mn(NO_3)_2.4H_2O$ (2 mol%)**	**EtOH**	**96**
3	$Cr(NO_3)_3.9H_2O$ (2 mol%)	EtOH	5
4	$Mg(NO_3)_2.6H_2O$ (2 mol%)	EtOH	15
5	$Bi(NO_3)_3.6H_2O$ (2 mol%)	EtOH	20
6	$AgNO_3$ (2 mol%)	EtOH	10
7	$Cu(NO_3)_2.3H_2O$ (2 mol%)	EtOH	30
8	$Ce(NO_3)_3.6H_2O$ (2 mol%)	EtOH	35
9	$Cd(NO_3)_2.4H_2O$ (2 mol%)	EtOH	38
10	$Zn(NO_3)_2.6H_2O$ (2 mol%)	EtOH	78
11	$La(NO_3)_3.6H_2O$ (2 mol%)	EtOH	82
12	$Mn(NO_3)_2.4H_2O$ (2 mol%)	EtOAc	65
13	$Mn(NO_3)_2.4H_2O$ (2 mol%)	CH_3CN	50
14	$Mn(NO_3)_2.4H_2O$ (2 mol%)	DCE	70
15	$Mn(NO_3)_2.4H_2O$ (2 mol%)	MeOH	80
16	$Mn(NO_3)_2.4H_2O$ (1 mol%)	EtOH	90
17	$Mn(NO_3)_2.4H_2O$ (5 mol%)	EtOH	96

[a]Reaction conditions: **1** (1.0 mmol), **6** (1.0 mmol), $Mn(NO_3)_2.4H_2O$ (2 mol%), solvent (5.0 mL).
[b]Isolated yield.

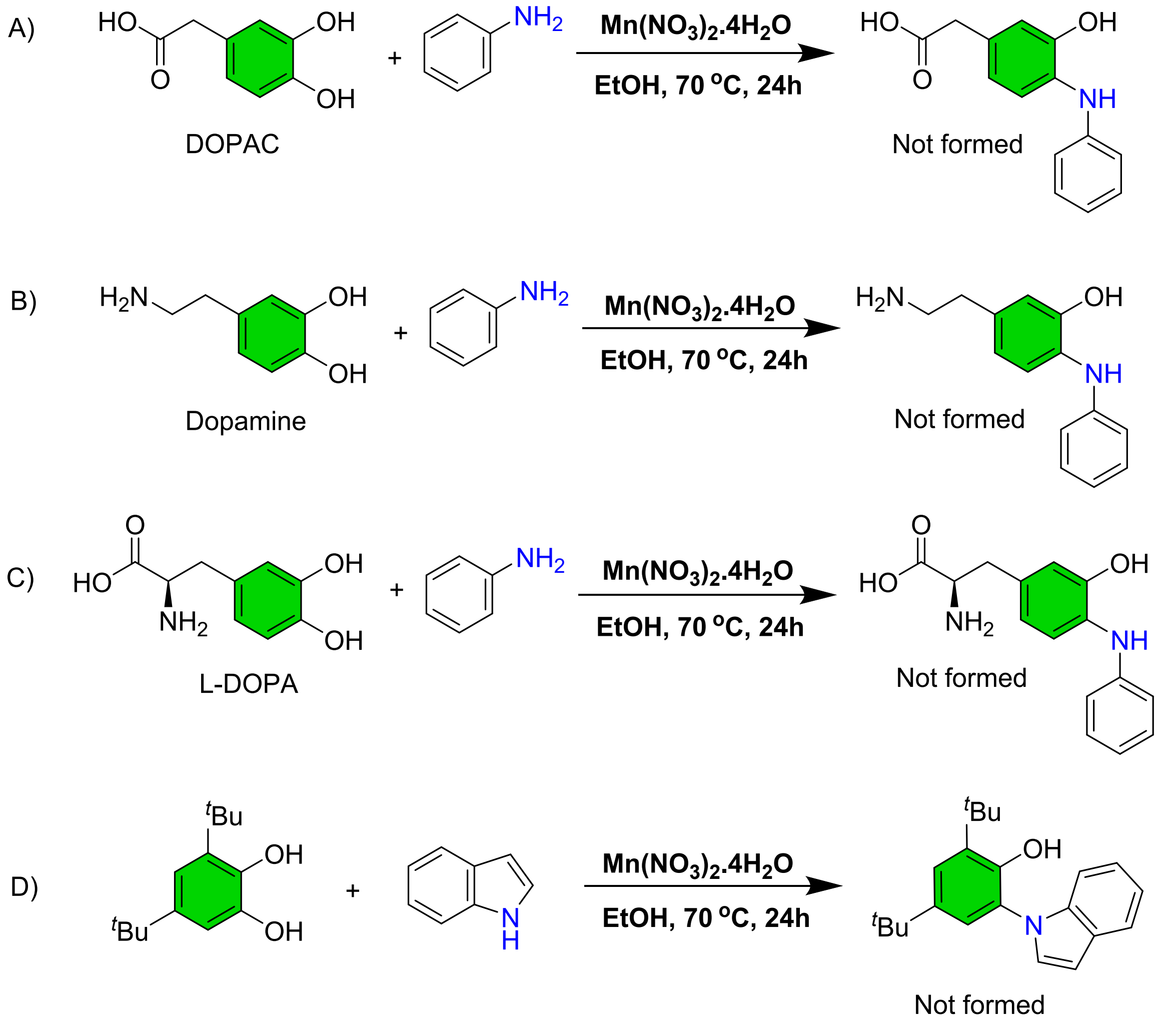

Scheme S1. The reaction of amine (1.0 mmol) and catechol derivatives (1.0 mmol) in the presence of $Mn(NO_3)_2$ (5 mol%) in EtOH (5 mL) at 70 °C.

Product characterization data:

2,4-di-*tert*-butyl-6-(*p*-tolylamino)phenol (3a).[1]
Purified by column chromatography on silica gel and eluted with ethyl acetate/petroleum ether (5:100). Isolated yield: 296 mg, 95%. White solid (m.p: 146-148 °C); ^{1}H NMR (400 MHz, $CDCl_3$) δ 1.29 (s, 9H), 1.48 (s, 9H), 2.30 (s, 3H), 4.95 (br, 1H), 6.50 (br, 1H), 6.63 (d, *J* = 7.7 Hz, 2H), 7.02 – 7.08 (m, 3H), 7.26 (d, *J* = 2.5 Hz, 1H); ^{13}C{^{1}H} NMR (100 MHz, $CDCl_3$) δ 20.5, 29.5, 31.6, 34.4, 35.0, 115.3, 121.3, 121.8, 128.2, 128.9, 129.8, 134.9, 142.1, 144.4, 149.4. Spectral data were found to match those reported for this product.[1]

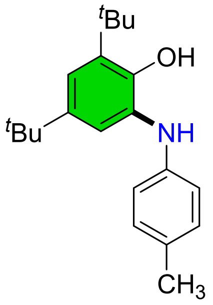

2,4-di-*tert*-butyl-6-((4-ethylphenyl)amino)phenol (3b).
Purified by column chromatography on silica gel and eluted with ethyl acetate/petroleum ether (5:100). Isolated yield: 306 mg, 94%. Yellow liquid; FTIR (KBr) υ: 3358 (OH), 3328 (NH) cm^{-1}; ^{1}H NMR (400 MHz, $CDCl_3$) δ 1.13 (t, *J* = 7.6 Hz, 3H), 1.18 (s, 9H), 1.36 (s, 9H), 2.49 (q, *J* = 8.0 Hz, 2H), 4.99 (br, 1H), 6.43 (br, 1H), 6.55 (d, *J* = 7.2 Hz, 2H), 6.96-6.99 (m, 3H), 7.14 (d, *J* = 3.2 Hz, 1H); ^{13}C{^{1}H} NMR (100 MHz, $CDCl_3$) δ 15.9, 28.0, 29.5, 31.6, 34.4, 35.0, 115.4, 121.4, 121.9, 128.1, 128.7, 135.3, 135.9, 142.2, 144.4, 149.3. Anal. Calcd for $C_{22}H_{31}NO$: C, 81.18; H, 9.60; N, 4.30; Found: C, 81.11; H, 9.51; N, 4.24.

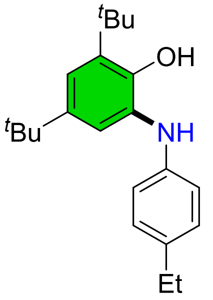

2,4-di-*tert*-butyl-6-((4-chlorophenyl)amino)phenol (3c).[1]
Purified by column chromatography on silica gel and eluted with ethyl acetate/petroleum ether (5:100). Isolated yield: 309 mg, 93%. White solid (m.p: 154-155 °C); ^{1}H NMR (400 MHz, $CDCl_3$) δ 1.31 (s, 9H), 1.48 (s, 9H), 5.06 (br, 1H), 6.36 (br, 1H), 6.64 (d, *J* = 8.3 Hz, 2H), 7.04 (d, *J* = 3.4 Hz, 1H), 7.19 (d, *J* = 8.3 Hz, 2H), 7.28 (d, *J* = 3.2 Hz, 1H). ^{13}C{^{1}H} NMR (100 MHz, $CDCl_3$) δ 29.5, 31.6, 34.4, 35.0, 116.3, 121.3, 122.3, 124.6, 127.4, 129.2, 135.5, 142.4, 145.4, 149.2. Spectral data were found to match those reported for this product.[1]

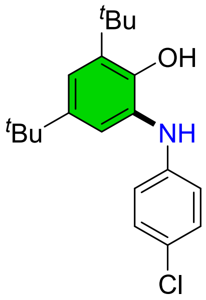

2-((4-bromophenyl)amino)-4,6-di-*tert*-butylphenol (3d).[2]
Purified by column chromatography on silica gel and eluted with ethyl acetate/petroleum ether (5:100). Isolated yield: 342 mg, 91%. White solid (m.p: 161-163 °C); ^{1}H NMR (400 MHz, $CDCl_3$) δ 1.26 (s, 9H), 1.43 (s, 9H), 5.01 (br, 1H), 6.30 (br, 1H), 6.55 (d, J = 8.8 Hz, 2H), 7.00 (d, J = 2.7 Hz, 1H), 7.23 (d, J = 3.0 Hz, 1H), 7.29 (d, J = 8.8 Hz, 2H); ^{13}C{^{1}H} NMR (100 MHz, $CDCl_3$) δ 29.5, 31.6, 34.4, 35.0, 111.8, 116.8, 121.4, 122.4, 127.2, 132.1, 135.5, 142.4, 145.9, 149.3. Spectral data were found to match those reported for this product.[2]

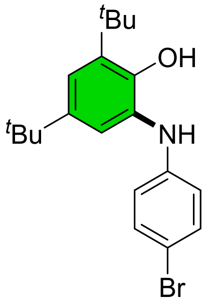

4-((3,5-di-*tert*-butyl-2-hydroxyphenyl)amino)benzonitrile (3e).
Purified by column chromatography on silica gel and eluted with ethyl acetate/petroleum ether (5:100). Isolated yield: 297 mg, 92%. Yellow liquid; FTIR (KBr) υ: 3415 (OH), 3347 (NH), 2223 (CN) cm-1; ^{1}H NMR (400 MHz, $CDCl_3$) δ 1.27 (s, 9H), 1.43 (s, 9H), 5.55 (br, 1H), 5.99 (br, 1H), 6.67 (d, J = 8.8 Hz, 2H), 7.01 (d, J = 3.1 Hz, 1H), 7.28 (d, J = 2.4 Hz, 1H), 7.44 (d, J = 8.7 Hz, 2H); ^{13}C{^{1}H} NMR (100 MHz, $CDCl_3$) δ 29.5, 31.6, 34.4, 35.1, 101.6, 114.7, 119.9, 121.7, 123.1, 125.6, 133.8, 136.1, 142.9, 149.1, 150.6. Anal. Calcd for $C_{21}H_{26}N_2O$: C, 78.22; H, 8.13; N, 8.69; Found: C, 78.16; H, 8.07; N, 8.58.

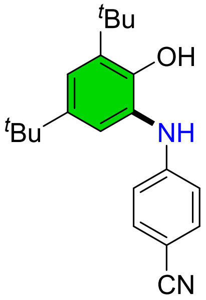

2,4-di-*tert*-butyl-6-(phenylamino)phenol (3f).[1]
Purified by column chromatography on silica gel and eluted with ethyl acetate/petroleum ether (5:100). Isolated yield: 262 mg, 88%. White solid (m.p: 149-151 °C); ^{1}H NMR (400 MHz, $CDCl_3$) δ 1.19 (s, 9H), 1.37 (s, 9H), 4.91 (br, 1H), 6.35 (br, 1H), 6.60 (d, J = 8.7 Hz, 2H), 6.78 (t, J = 7.3 Hz, 1H), 6.97 (d, J = 2.4 Hz, 1H), 7.13 (d, J = 8.5 Hz, 2H), 7.15 (d, J = 2.9 Hz, 1H); ^{13}C{^{1}H} NMR (100 MHz, $CDCl_3$) δ 29.5, 31.6, 34.4, 35.0, 115.1, 119.8, 121.6, 122.1, 127.7, 129.4, 135.3, 142.2, 146.8, 149.4. Spectral data were found to match those reported for this product.[1]

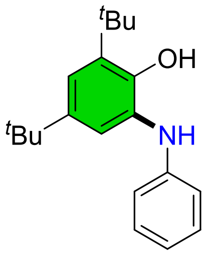

2,4-di-*tert*-butyl-6-((3-chlorophenyl)amino)phenol (3g).

Purified by column chromatography on silica gel and eluted with ethyl acetate/petroleum ether (5:100). Isolated yield: 295 mg, 89%. White solid (m.p: 121-123 ^{o}C); FTIR (KBr) υ: 3384 (OH), 3328 (NH) cm^{-1}; ^{1}H NMR (400 MHz, $CDCl_3$) δ 1.27 (s, 9H), 1.44 (s, 9H), 5.05 (br, 1H), 6.26 (br, 1H), 6.54 (dd, *J* = 8.0, 3.4 Hz, 1H), 6.66 (t, *J* = 2.4 Hz, 1H), 6.82 (dd, *J* = 7.9, 2.9 Hz, 1H), 7.01 (d, *J* = 2.8 Hz, 1H), 7.11 (t, *J* = 8.0 Hz, 1H), 7.24 (d, *J* = 2.9 Hz, 1H); ^{13}C{^{1}H} NMR (100 MHz, $CDCl_3$) δ 29.5, 31.6, 34.4, 35.0, 113.3, 115.0, 119.8, 121.6, 122.5, 126.8, 130.4, 135.1, 135.6, 142.5, 148.1, 149.3. Anal. Calcd for $C_{20}H_{26}ClNO$: C, 72.38; H, 7.90; N, 4.22; Found: C, 72.29; H, 7.81; N, 4.31.

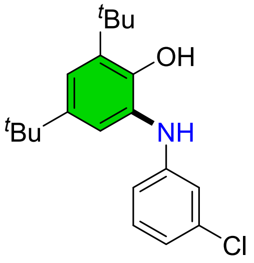

2,4-di-*tert*-butyl-6-((3-methoxyphenyl)amino)phenol (3h).

Purified by column chromatography on silica gel and eluted with ethyl acetate/petroleum ether (5:100). Isolated yield: 295 mg, 90%. White solid (m.p: 130-132 ^{o}C); FTIR (KBr) υ: 3405 (OH), 3320 (NH) cm^{-1}; ^{1}H NMR (400 MHz, $CDCl_3$) δ 1.26 (s, 9H), 1.44 (s, 9H), 3.73 (s, 3H), 5.00 (br, 1H), 6.20 (br, 1H), 6.30 (d, *J* = 8.0 Hz, 1H), 6.35 (s, 1H), 6.41 (d, *J* = 8.1 Hz, 1H), 7.03 (d, *J* = 2.4 Hz, 1H), 7.11 (t, *J* = 8.1 Hz, 1H), 7.21 (d, *J* = 2.3 Hz, 1H); ^{13}C{^{1}H} NMR (100 MHz, $CDCl_3$) δ 29.5, 31.6, 34.4, 35.0, 55.1, 101.1, 105.0, 107.9, 121.6, 122.1, 127.4, 130.1, 135.3, 142.2, 148.2, 149.3, 160.8. Anal. Calcd for $C_{21}H_{29}NO_2$: C, 77.02; H, 8.93; N, 4.28; Found: C, 76.95; H, 8.87; N, 4.36.

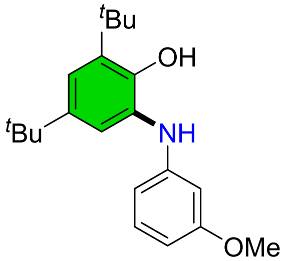

3-((3,5-di-*tert*-butyl-2-hydroxyphenyl)amino)benzonitrile (3i).

Purified by column chromatography on silica gel and eluted with ethyl acetate/petroleum ether (5:100). Isolated yield: 284 mg, 88%. Colorless liquid; FTIR (KBr) υ: 3417 (OH), 3352 (NH), 2221 (CN) cm^{-1}; ^{1}H NMR (400 MHz, $CDCl_3$) δ 1.27 (s, 9H), 1.44 (s, 9H), 5.22 (br, 1H), 6.17 (br, 1H), 6.89 (dd, *J* = 7.8, 2.8 Hz, 2H), 6.99 (d, *J* = 3.2 Hz, 1H), 7.11 (dd, *J* = 7.6, 2.6 Hz, 1H), 7.26 (d, *J* = 5.8 Hz, 1H), 7.28 (s, 1H); ^{13}C{^{1}H} NMR (100 MHz, $CDCl_3$) δ 149.3, 147.5, 142.8, 135.9, 130.2, 126.2, 123.3, 123.0, 121.5, 119.3, 119.1, 117.7, 113.2, 35.1, 34.4, 31.6, 29.5. Anal. Calcd for $C_{21}H_{26}N_2O$: C, 78.06; H, 8.05; N, 8.63; Found: C,77.98; H, 8.13; N, 8.72.

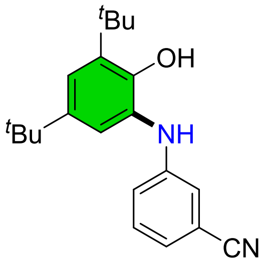

2-((3,5-di-*tert*-butyl-2-hydroxyphenyl)amino)benzonitrile (3j).[3]
Purified by column chromatography on silica gel and eluted with ethyl acetate/petroleum ether (5:100). Isolated yield: 271 mg, 84%. Colorless liquid; ^{1}H NMR (400 MHz, $CDCl_3$) δ 1.28 (s, 9H), 1.44 (s, 9H), 5.82 (br, 1H), 5.99 (br, 1H), 6.50 (d, J = 7.5 Hz, 1H), 6.85 (t, J = 7.6 Hz, 1H), 7.01 (d, J = 3.2 Hz, 1H), 7.29 (d, J = 2.4 Hz, 1H), 7.32 – 7.36 (m, 1H), 7.51 (d, J = 6.3 Hz, 1H); ^{13}C{^{1}H} NMR (100 MHz, $CDCl_3$) δ 29.5, 31.6, 34.4, 35.1, 97.8, 113.9, 117.5, 119.2, 122.1, 123.3, 125.2, 132.6, 134.4, 136.1, 142.8, 149.3, 149.7. Spectral data were found to match those reported for this product.[3]

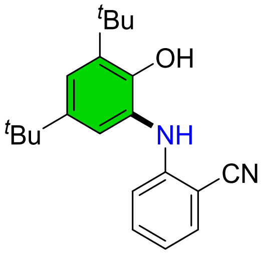

2,4-di-*tert*-butyl-6-((2-(trifluoromethyl)phenyl)amino)phenol (3k).[4]
Purified by column chromatography on silica gel and eluted with ethyl acetate/petroleum ether (5:100). Isolated yield: 314 mg, 86%. Yellow liquid; ^{1}H NMR (400 MHz, $CDCl_3$) δ 1.34 (s, 9H), 1.50 (s, 9H), 5.69 (br, 1H), 6.27 (br, 1H), 6.61 (d, J = 8.3 Hz, 1H), 6.93 (t, J = 7.6 Hz, 1H), 7.07 (d, J = 2.3 Hz, 1H), 7.32-7.37 (m, 2H), 7.59 (d, J = 7.9 Hz, 1H); ^{13}C{^{1}H} NMR (100 MHz, $CDCl_3$) δ 29.5, 31.6, 34.4, 35.1, 115.5, 118.8, 122.3, 122.9, 126.0, 126.5 (q, J = 5.5 Hz), 133.2, 135.8, 142.7, 144.9, 149.5. Spectral data were found to match those reported for this product.[4]

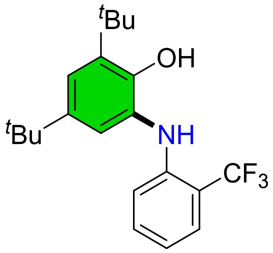

2,4-di-*tert*-butyl-6-((2-chlorophenyl)amino)phenol (3l).[5]
Purified by column chromatography on silica gel and eluted with ethyl acetate/petroleum ether (5:100). Isolated yield: 282 mg, 85%. White solid (m.p: 131-133 °C); ^{1}H NMR (400 MHz, $CDCl_3$) δ 1.27 (s, 9H), 1.44 (s, 9H), 5.60 (br, 1H), 6.28 (br, 1H), 6.47 (dd, J = 8.2, 1.5 Hz, 1H), 6.77 (td, J = 7.6, 1.5 Hz, 1H), 7.00 (d, J = 2.3 Hz, 1H), 7.04 – 7.08 (m, 1H), 7.26 (d, J = 1.3 Hz, 1H), 7.34 (dd, J = 8.0, 1.5 Hz, 1H); ^{13}C{^{1}H} NMR (100 MHz, $CDCl_3$) δ 29.5, 31.6, 34.4, 35.1, 114.7, 119.9, 120.4, 122.0, 122.6, 126.4, 127.9, 129.3, 135.6, 142.5, 143.1, 149.6. Spectral data were found to match those reported for this product.[5]

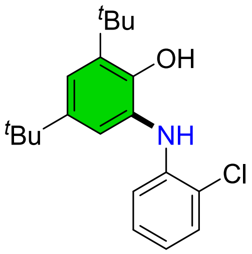

2,4-di-*tert*-butyl-6-((2-iodophenyl)amino)phenol (3m).[6]

Purified by column chromatography on silica gel and eluted with ethyl acetate/petroleum ether (5:100). Isolated yield: 368 mg, 87%. Brown liquid; ^{1}H NMR (400 MHz, $CDCl_3$) δ 1.20 (s, 9H), 1.37 (s, 9H), 5.37 (br, 1H), 6.21 (br, 1H), 6.33 (d, *J* = 8.2 Hz, 1H), 6.50 (t, *J* = 7.6 Hz, 1H), 6.92 (d, *J* = 3.7 Hz, 1H), 7.05 (t, *J* = 7.6 Hz, 1H), 7.19 (d, *J* = 2.8 Hz, 1H), 7.66 (d, *J* = 7.8 Hz, 1H); ^{13}C{^{1}H} NMR (100 MHz, $CDCl_3$) δ 29.5, 31.6, 34.4, 35.1, 86.3, 114.2, 121.2, 122.1, 122.7, 127.0, 129.5, 135.7, 139.1, 142.5, 146.6, 149.5. Spectral data were found to match those reported for this product.[6]

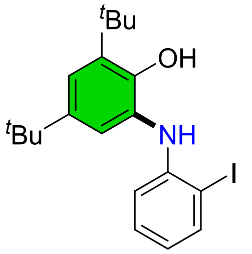

2,4-di-*tert*-butyl-6-((2,4-dichlorophenyl)amino)phenol (3n).[5]

Purified by column chromatography on silica gel and eluted with ethyl acetate/petroleum ether (5:100). Isolated yield: 311 mg, 85%. Yellow liquid; ^{1}H NMR (400 MHz, $CDCl_3$) δ 1.30 (s, 9H), 1.47 (s, 9H), 5.59 (br, 1H), 6.20 (br, 1H), 6.43 (d, *J* = 8.7 Hz, 1H), 7.00 (d, *J* = 2.9 Hz, 1H), 7.06 (dd, *J* = 8.5, 2.6 Hz, 1H), 7.29 (d, *J* = 2.4 Hz, 1H), 7.38 (d, *J* = 3.2 Hz, 1H); ^{13}C{^{1}H} NMR (100 MHz, $CDCl_3$) δ 29.5, 31.6, 34.4, 35.1, 115.4, 120.7, 121.8, 122.9, 124.1, 126.1, 128.0, 128.9, 135.8, 141.9, 142.7, 149.4. Spectral data were found to match those reported for this product.[5]

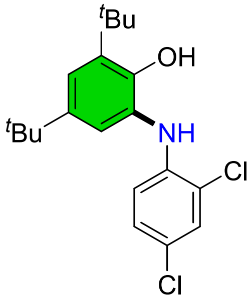

5,7-di-*tert*-butyl-2,2-dimethyl-3-(*p*-tolyl)-2,3-dihydrobenzo[*d*]oxazole (5a).

Purified by column chromatography on silica gel and eluted with ethyl acetate/petroleum ether (1:100). Isolated yield: 327 mg, 93%. Yellow liquid; FTIR (KBr) υ: 2998, 1588, 1487, 1381, 1230 cm-1; ^{1}H NMR (400 MHz, $CDCl_3$) δ 1.16 (s, 9H), 1.30 (s, 9H), 1.48 (s, 6H), 2.29 (s, 3H), 6.38 (d, *J* = 2.8 Hz, 1H), 6.58 (d, *J* = 3.1 Hz, 1H), 7.10 (d, *J* = 8.4 Hz, 2H), 7.16 (d, *J* = 8.5 Hz, 2H); ^{13}C{^{1}H} NMR (100 MHz, $CDCl_3$) δ 21.0, 26.7, 29.4, 31.8, 34.0, 34.7, 102.8, 104.4, 113.3, 125.8, 130.0, 130.5, 135.1, 137.3, 138.9, 143.1, 144.7. Anal. Calcd for $C_{24}H_{33}NO$: C, 82.00; H, 9.46; N, 3.98; Found: C, 81.91; H, 9.38; N, 4.06.

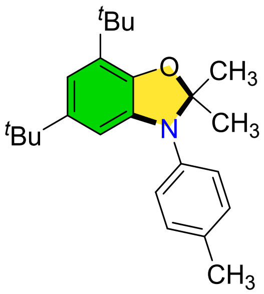

5,7-di-*tert*-butyl-3-(4-chlorophenyl)-2,2-dimethyl-2,3-dihydrobenzo[*d*]oxazole (5b).
Purified by column chromatography on silica gel and eluted with ethyl acetate/petroleum ether (1:100). Isolated yield: 335 mg, 90%. Yellow liquid; FTIR (KBr) υ: 2990, 1596, 1471, 1386, 1235 cm^{-1}; ^{1}H NMR (400 MHz, $CDCl_3$) δ 1.17 (s, 9H), 1.30 (s, 9H), 1.50 (s, 6H), 6.41 (d, J = 1.9 Hz, 1H), 6.62 (d, J = 2.5 Hz, 1H), 7.20 (d, J = 9.5 Hz, 2H), 7.26 (d, J = 8.8 Hz, 2H).; $^{13}C\{^{1}H\}$ NMR (100 MHz, $CDCl_3$) δ 26.7, 29.4, 31.8, 34.0, 34.7, 102.8, 104.7, 114.1, 126.6, 129.5, 130.2, 130.9, 136.4, 140.4, 143.2, 144.7. Anal. Calcd for $C_{23}H_{30}ClNO$: C, 74.27; H, 8.13; N, 3.77; Found: C, 74.19; H, 8.02; N, 3.65.

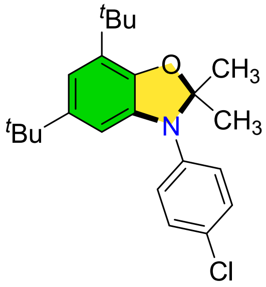

3-(4-bromophenyl)-5,7-di-*tert*-butyl-2,2-dimethyl-2,3-dihydrobenzo[*d*]oxazole (5c).
Purified by column chromatography on silica gel and eluted with ethyl acetate/petroleum ether (1:100). Isolated yield: 362 mg, 87%. Yellow liquid; FTIR (KBr) υ: 2982, 1593, 1477, 1394, 1231 cm^{-1}; ^{1}H NMR (300 MHz, $CDCl_3$) δ 1.17 (s, 9H), 1.30 (s, 9H), 1.50 (s, 6H), 6.43 (d, J = 2.0 Hz, 1H), 6.62 (d, J = 2.6 Hz, 1H), 7.14 (d, J = 8.8 Hz, 2H), 7.41 (d, J = 8.7 Hz, 2H); $^{13}C\{^{1}H\}$ NMR (75 MHz, $CDCl_3$) δ 26.8, 29.4, 31.8, 34.0, 34.7, 102.7, 104.7, 114.1, 117.8, 126.8, 130.9, 132.5, 136.3, 140.9, 143.2, 144.7. Anal. Calcd for $C_{23}H_{30}BrNO$: C, 66.34; H, 7.26; N, 3.36; Found: C, 66.27; H, 7.18; N, 3.27.

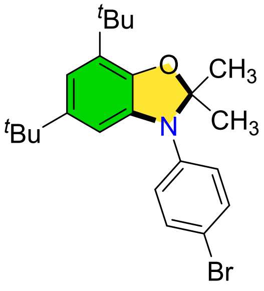

5,7-di-*tert*-butyl-3-(4-isopropylphenyl)-2,2-dimethyl-2,3-dihydrobenzo[*d*]oxazole (5d).
Purified by column chromatography on silica gel and eluted with ethyl acetate/petroleum ether (1:100). Isolated yield: 341 mg, 90%. Yellow liquid; FTIR (KBr) υ: 2998, 1588, 1479, 1392, 1241 cm^{-1}; ^{1}H NMR (400 MHz, $CDCl_3$) δ 1.17 (s, 9H), 1.20 (d, J = 6.9 Hz, 6H), 1.30 (s, 9H), 1.48 (s, 6H), 2.85 (hept, J = 6.9 Hz, 1H), 6.41 (d, J = 2.9 Hz, 1H), 6.58 (d, J = 2.3 Hz, 1H), 7.14 (d, J = 8.6 Hz, 2H), 7.18 (d, J = 8.5 Hz, 2H); $^{13}C\{^{1}H\}$ NMR (100 MHz, $CDCl_3$) δ

24.0, 26.7, 29.5, 31.8, 33.7, 34.0, 34.7, 102.8, 104.3, 113.2, 125.6, 127.3, 130.4, 137.3, 139.1, 143.1, 144.6, 145.8. Anal. Calcd for $C_{26}H_{37}NO$: C, 82.27; H, 9.83; N, 3.69; Found: C, 82.19; H, 9.74; N, 3.61.

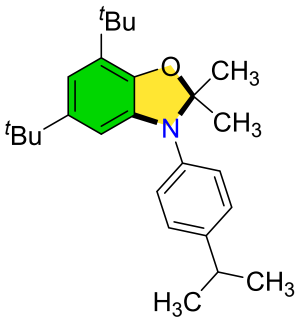

5,7-di-*tert*-butyl-2,2-dimethyl-3-phenyl-2,3-dihydrobenzo[*d*]oxazole (5e).
Purified by column chromatography on silica gel and eluted with ethyl acetate/petroleum ether (1:100). Isolated yield: 300 mg, 89%. Yellow liquid; FTIR (KBr) υ: 3010, 1583, 1476, 1370, 1244 cm^{-1}; ^{1}H NMR(400 MHz, $CDCl_3$) δ 1.17 (s, 9H), 1.30 (s, 9H), 1.51 (s, 6H), 6.45 (d, J = 2.5 Hz, 1H), 6.60 (d, J = 2.5 Hz, 1H), 7.12 (t, J = 8.5 Hz, 1H), 7.22-7.31 (m, J = 6.7 Hz, 4H); $^{13}C\{^{1}H\}$ NMR (100 MHz, $CDCl_3$) δ 26.8, 29.4, 31.8, 34.0, 34.7, 102.8, 104.6, 113.6, 125.1, 125.4, 129.4, 130.6, 136.8, 141.7, 143.1, 144.7. Anal. Calcd for $C_{23}H_{31}NO$: C, 81.85; H, 9.26; N, 4.15; Found: C, 81.77; H, 9.17; N, 4.08.

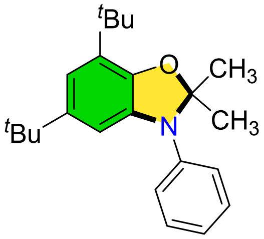

5,7-di-*tert*-butyl-3-(3-chlorophenyl)-2,2-dimethyl-2,3-dihydrobenzo[*d*]oxazole (5f).
Purified by column chromatography on silica gel and eluted with ethyl acetate/petroleum ether (1:100). Isolated yield: 316 mg, 85%. Yellow liquid; FTIR (KBr) υ: 3005, 1604, 1487, 1395, 1241 cm^{-1}; ^{1}H NMR (400 MHz, $CDCl_3$) δ 1.18 (s, 9H), 1.30 (s, 9H), 1.53 (s, 6H), 6.50 (d, J = 2.5 Hz, 1H), 6.64 (d, J = 2.4 Hz, 1H), 7.07 (dd, J = 7.8, 3.8 Hz, 1H), 7.14 (dd, J = 8.1, 3.9 Hz, 1H), 7.19 (d, J = 2.4 Hz, 1H), 7.26 – 7.28 (m, 1H); $^{13}C\{^{1}H\}$ NMR (100 MHz, $CDCl_3$) δ 26.9, 29.4, 31.8, 34.0, 34.7, 102.8, 105.0, 114.3, 122.7, 124.7, 124.8, 130.3, 130.9, 134.9, 135.9, 143.2, 143.3, 144.8. Anal. Calcd for $C_{23}H_{30}ClNO$: C, 74.27; H, 8.13; N, 3.77; Found: C, 74.18; H, 8.06; N, 3.69.

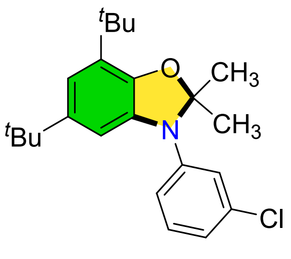

5,7-di-*tert*-butyl-3-(3-methoxyphenyl)-2,2-dimethyl-2,3-dihydrobenzo[*d*]oxazole (5g).
Purified by column chromatography on silica gel and eluted with ethyl acetate/petroleum ether (1:100). Isolated yield: 331 mg, 90%. Yellow liquid; FTIR (KBr) υ: 2993, 1590, 1474, 1388, 1230 cm^{-1}; ^{1}H NMR (400 MHz, $CDCl_3$) δ 1.18 (s, 9H), 1.31 (s, 9H), 1.53 (s, 6H), 3.72 (s, 3H), 6.52 (s, 1H), 6.61 (s, 1H), 6.80-6.85 (m, 2H), 7.21 (d, J = 8.4 Hz, 1H); $^{13}C\{^{1}H\}$ NMR (100 MHz, $CDCl_3$) δ 18.6, 29.5, 31.1, 31.6, 51.9, 55.1, 98.6, 102.3, 115.4, 122.1, 124.6,

126.0, 128.2, 130.1, 144.6, 160.2. Anal. Calcd for $C_{24}H_{33}NO_2$: C, 78.43; H, 9.05; N, 3.81; Found: C, 78.35; H, 8.96; N, 3.72.

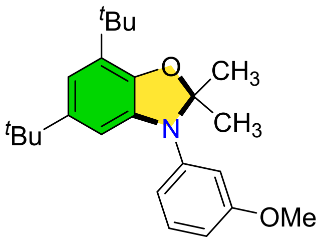

2-(5,7-di-*tert*-butyl-2,2-dimethylbenzo[*d*]oxazol-3(2*H*)-yl)benzonitrile (5h).
Purified by column chromatography on silica gel and eluted with ethyl acetate/petroleum ether (1:100). Isolated yield: 305 mg, 84%. Yellow liquid; FTIR (KBr) υ: 3008, 2228, 1597, 1479, 1380, 1240 cm-1; ^{1}H NMR (400 MHz, $CDCl_3$) δ 1.19 (s, 9H), 1.30 (s, 9H), 1.55 (s, 6H), 6.50 (d, *J* = 2.4 Hz, 1H), 6.69 (d, *J* = 1.9 Hz, 1H), 7.34 – 7.40 (m, 2H), 7.48 – 7.52 (m, 2H); $^{13}C\{^{1}H\}$ NMR (100 MHz, $CDCl_3$) δ 26.9, 29.4, 31.8, 34.1, 34.7, 102.7, 105.0, 113.5, 115.1, 118.5, 127.3, 127.7, 128.5, 130.3, 131.4, 132.4, 135.1, 143.1, 143.4. Anal. Calcd for $C_{24}H_{30}N_2O$: C, 79.52; H, 8.34; N, 7.73; Found: C, 79.44; H, 8.26; N, 7.66.

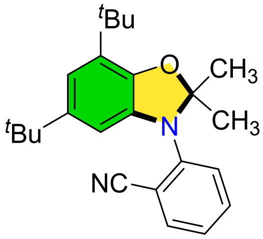

5,7-di-*tert*-butyl-3-(4-chlorophenyl)-2-ethyl-2-methyl-2,3-dihydrobenzo[*d*]oxazole (5i).
Purified by column chromatography on silica gel and eluted with ethyl acetate/petroleum ether (1:100). Isolated yield: 320 mg, 83%. pale yellow liquid; FTIR (KBr) υ: 2994, 1590, 1477, 1381, 1232 cm-1; ^{1}H NMR (400 MHz, $CDCl_3$) δ 0.94 (t, *J* = 7.4 Hz, 3H), 1.17 (s, 9H), 1.30 (s, 9H), 1.40 (s, 3H), 1.40-1.92 (m, 2H), 6.44 (d, *J* = 2.6 Hz, 1H), 6.60 (d, *J* = 1.9 Hz, 1H), 7.18 (d, *J* = 8.8 Hz, 2H), 7.25 (d, *J* = 8.9 Hz, 2H); $^{13}C\{^{1}H\}$ NMR (100 MHz, $CDCl_3$) δ 7.8, 25.0, 29.4, 31.8, 33.0, 34.0, 34.7, 104.4, 104.8, 113.8, 125.9, 129.5, 129.7, 130.4, 136.6, 140.4, 142.9, 145.1. Anal. Calcd for $C_{24}H_{32}ClNO$: C, 74.68; H, 8.36; N, 3.63; Found: C, 74.59; H, 8.28; N, 3.61.

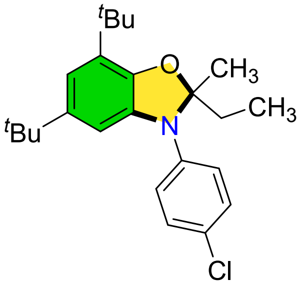

5,7-di-*tert*-butyl-2-ethylbenzo[*d*]oxazole (7a).[7]
Purified by column chromatography on silica gel and eluted with ethyl acetate/petroleum ether (1:100). Isolated yield: 249 mg, 96%. Colorless liquid; ^{1}H NMR (400 MHz, $CDCl_3$) δ 1.29 (s, 9H), 1.37 (t, *J* = 7.1 Hz, 3H), 1.40 (s, 9H), 2.89 (q, *J* = 7.6 Hz, 2H), 7.17 (d, *J* = 1.7 Hz, 1H), 7.48 (d, *J* = 3.1 Hz, 1H); $^{13}C\{^{1}H\}$ NMR (100 MHz, $CDCl_3$) δ 11.1, 22.2, 29.9, 31.9, 34.4, 35.0, 113.7, 118.7, 133.5, 141.4, 147.2, 167.6. Spectral data were found to match those reported for this product.[7]

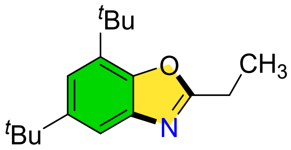

5,7-di-*tert*-butyl-2-propylbenzo[*d*]oxazole (7b).[8]
Purified by column chromatography on silica gel and eluted with ethyl acetate/petroleum ether (1:100). Isolated yield: 254 mg, 93%. Colorless liquid; ^{1}H NMR (400 MHz, $CDCl_3$) δ 0.99 (t, J = 7.4 Hz, 3H), 1.30 (s, 9H), 1.40 (s, 9H), 1.80 – 1.89 (m, 2H), 2.84 (t, J = 7.4 Hz, 2H), 7.17 (d, J = 2.1 Hz, 1H), 7.47 (d, J = 1.9 Hz, 1H); ^{13}C{^{1}H} NMR (100 MHz, $CDCl_3$) δ 13.8, 20.4, 29.9, 30.6, 31.9, 34.4, 35.0, 113.7, 118.7, 133.5, 141.4, 147.0, 147.2, 166.6. Spectral data were found to match those reported for this product.[8]

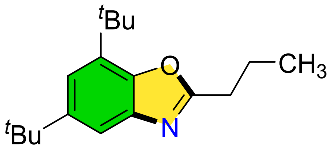

5,7-di-*tert*-butyl-2-methylbenzo[*d*]oxazole (7c).[9]
Purified by column chromatography on silica gel and eluted with ethyl acetate/petroleum ether (1:100). Isolated yield: 211 mg, 86%. Colorless liquid; ^{1}H NMR (400 MHz, $CDCl_3$) δ 1.29 (s, 9H), 1.39 (s, 9H), 2.57 (s, 3H), 7.16 (d, J = 1.8 Hz, 1H), 7.44 (d, J = 2.2 Hz, 1H); ^{13}C{^{1}H} NMR (100 MHz, $CDCl_3$) δ 14.7, 29.9, 31.9, 34.4, 35.0, 113.6, 118.8, 133.4, 141.6, 147.2, 163.2. Spectral data were found to match those reported for this product.[9]

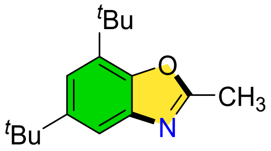

5,7-di-*tert*-butyl-2-heptylbenzo[*d*]oxazole (7d).[9]
Purified by column chromatography on silica gel and eluted with ethyl acetate/petroleum ether 1:100). Isolated yield: 297 mg, 90%. Brown liquid; ^{1}H NMR (400 MHz, $CDCl_3$) δ 0.91 (t, J = 6.7 Hz, 3H), 1.29 – 1.34 (m, 4H), 1.40 (s, 9H), 1.42 – 1.47 (m, 4H), 1.50 (s, 9H), 1.88 – 1.95 (m, 2H), 2.96 (t, J = 7.6 Hz, 2H), 7.27 (d, J = 2.1 Hz, 1H), 7.58 (d, J = 2.4 Hz, 1H); ^{13}C{^{1}H} NMR (100 MHz, $CDCl_3$) δ 14.1, 22.6, 27.0, 28.7, 29.0, 29.1, 29.9, 31.7, 31.9, 34.4, 35.0, 113.8, 118.6, 120.0, 133.4, 141.6, 147.1, 166.8. Spectral data were found to match those reported for this product.[9]

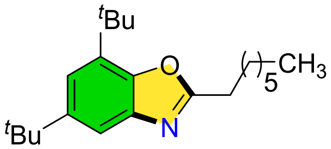

5,7-di-*tert*-butyl-2-isopropylbenzo[*d*]oxazole (7e).[7]
Purified by column chromatography on silica gel and eluted with ethyl acetate/petroleum ether (1:100). Isolated yield: 260 mg, 95%. Colorless liquid; ^{1}H NMR (400 MHz, $CDCl_3$) δ 1.39 (s, 9H), 1.49 (d, J = 6.8 Hz, 6H), 1.50 (s, 9H), 3.24-3.34 (m, 1H), 7.28 (d, J = 1.9 Hz, 1H), 7.61 (d, J = 2.7 Hz, 1H); ^{13}C{^{1}H} NMR (100 MHz, $CDCl_3$) δ 20.4, 28.8, 29.9, 31.9, 34.4, 35.0, 113.8, 118.8, 133.5, 141.0, 146.9, 147.3, 170.8. Spectral data were found to match those reported for this product.[7]

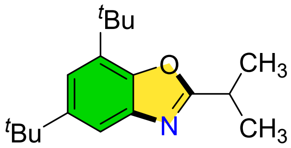

2-(5,7-di-*tert*-butylbenzo[*d*]oxazol-2-yl)ethan-1-ol (7f).[10]
Purified by column chromatography on silica gel and eluted with ethyl acetate/petroleum ether (1:100). Isolated yield: 242 mg, 88%. Colorless liquid; ^{1}H NMR (400 MHz, $CDCl_3$) δ 1.39 (s, 9H), 1.49 (s, 9H), 3.20 (t, J = 5.8 Hz, 2H), 4.17 (t, J = 5.9 Hz, 2H), 7.29 (d, J = 2.2 Hz, 1H), 7.56 (d, J = 2.8 Hz, 1H); ^{13}C{^{1}H} NMR (100 MHz, $CDCl_3$) δ 29.9, 31.6, 31.9, 34.4, 35.1, 59.1, 113.7, 119.1, 133.6, 141.0, 146.9, 147.5, 164.7. Spectral data were found to match those reported for this product.[10]

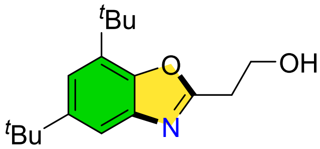

(5,7-di-*tert*-butylbenzo[*d*]oxazol-2-yl)methanol (7g).[7]
Purified by column chromatography on silica gel and eluted with ethyl acetate/petroleum ether (1:100). Isolated yield: 235 mg, 90%. Colorless liquid; ^{1}H NMR (400 MHz, $CDCl_3$) δ 1.39 (s, 9H), 1.48 (s, 9H), 4.99 (s, 2H), 5.34 (s, 1H), 7.31 (d, J = 1.8 Hz, 1H), 7.57 (d, J = 2.4 Hz, 1H); ^{13}C{^{1}H} NMR (100 MHz, $CDCl_3$) δ 29.9, 31.8, 34.4, 35.1, 57.8, 113.8, 119.6, 134.0, 140.5, 147.0, 147.9, 165.7. Spectral data were found to match those reported for this product.[7]

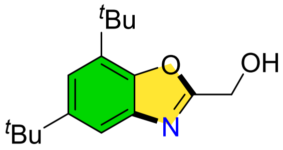

2-(5,7-di-*tert*-butylbenzo[*d*]oxazol-2-yl)-*N*,*N*-dimethylethan-1-amine (7h).[9]
Purified by column chromatography on silica gel and eluted with ethyl acetate/petroleum ether (1:100). Isolated yield: 227 mg, 75%. Colorless liquid; ^{1}H NMR (400 MHz, $CDCl_3$) δ 1.29 (s, 9H), 1.40 (s, 9H), 2.27 (s, 6H), 2.75 – 2.91 (m, 2H), 3.04 (t, J = 7.2 Hz, 2H), 7.16 (d, J = 1.6 Hz, 1H), 7.46 (d, J = 1.8 Hz, 1H); ^{13}C{^{1}H} NMR (100 MHz, $CDCl_3$) δ 164.9, 147.2, 147.1, 141.5, 133.5, 118.8, 113.8, 56.3, 45.2, 35.0, 34.4, 31.8, 29.9, 27.3. Spectral data were found to match those reported for this product.[9]

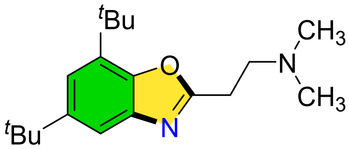

5,7-di-*tert*-butyl-2-(tetrahydrofuran-2-yl)benzo[*d*]oxazole (7i).[11]
Purified by column chromatography on silica gel and eluted with ethyl acetate/petroleum ether (1:100). Isolated yield: 259 mg, 86%. Colorless liquid; ^{1}H NMR (400 MHz, $CDCl_3$) δ 1.38 (s, 9H), 1.50 (s, 9H), 2.03 – 2.10 (m, 1H), 2.18 – 2.24 (m, 1H), 2.40 (q, J = 7.3 Hz, 2H), 4.03 (dd, J = 15.0, 6.6 Hz, 1H), 4.15 (dd, J = 14.6, 7.3 Hz, 1H), 5.25 (t, J = 6.4 Hz, 1H), 7.30 (d, J = 1.9 Hz, 1H), 7.61 (d, J = 2.8 Hz, 1H); ^{13}C{^{1}H} NMR (100 MHz, $CDCl_3$) δ 25.8, 30.0, 30.9, 31.9, 34.4, 35.0, 69.2, 73.9, 114.3, 119.4, 133.8, 141.0, 147.1, 147.5, 165.9. Spectral data were found to match those reported for this product.[11]

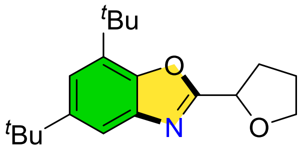

5,7-di-*tert*-butyl-2-(2-morpholinoethyl)benzo[*d*]oxazole (7j).[9]
Purified by column chromatography on silica gel and eluted with ethyl acetate/petroleum ether (1:40). Isolated yield: 293 mg, 85%. Colorless liquid; ^{1}H NMR (400 MHz, $CDCl_3$) δ 1.36 (s, 9H), 1.47 (s, 9H), 2.53 (t, J = 4.7 Hz, 4H), 2.93 (t, J = 7.5 Hz, 2H), 3.13 (t, J = 7.5 Hz, 2H), 3.69 (t, J = 4.7 Hz, 4H), 7.25 (d, J = 2.6 Hz, 1H), 7.54 (d, J = 2.1 Hz, 1H); ^{13}C{^{1}H} NMR (100 MHz, $CDCl_3$) δ 26.5, 29.9, 31.9, 34.4, 35.0, 53.4, 55.6, 66.9, 113.8, 118.8, 133.4, 141.5, 147.0, 147.2, 164.8. Spectral data were found to match those reported for this product.[9]

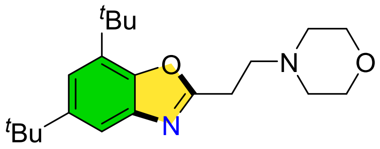

4-(5,7-di-*tert*-butylbenzo[*d*]oxazol-2-yl)cyclohexane-1-carboxylic acid (7k).[10]
Purified by column chromatography on silica gel and eluted with ethyl acetate/petroleum ether (10:100). Isolated yield: 293 mg, 82%. Colorless liquid; ^{1}H NMR (400 MHz, $CDCl_3$) δ 1.22 (s, 9H), 1.29 (s, 9H), 1.47 – 1.71 (m, 4H), 2.00 – 2.09 (m, 1H), 2.21 – 2.32 (m, 4H), 2.75 – 2.94 (m, 1H), 7.09 (d, J = 1.9 Hz, 1H), 7.18 (d, J = 1.1 Hz, 1H), 7.41 (s, 1H); ^{13}C{^{1}H} NMR (100 MHz, $CDCl_3$) δ 28.8, 29.9, 30.0, 31.9, 34.3, 35.0, 37.3, 44.5, 113.9, 118.7, 133.4, 141.3, 146.8, 147.1, 169.0. Spectral data were found to match those reported for this product.[10]

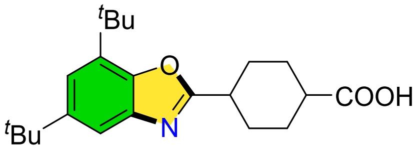

5,7-di-*tert*-butyl-2-phenylbenzo[*d*]oxazole (7l).[6]
Purified by column chromatography on silica gel and eluted with ethyl acetate/petroleum ether (1:100). Isolated yield: 286 mg, 93%. Colorless liquid; ^{1}H NMR (400 MHz, $CDCl_3$) δ 1.45 (s, 9H), 1.61 (s, 9H), 7.37 (d, J = 2.2 Hz, 1H), 7.55 – 7.59 (m, 3H), 7.73 (d, J = 1.9 Hz, 1H), 8.29 – 8.32 (m, 2H); ^{13}C{^{1}H} NMR (100 MHz, $CDCl_3$) δ 30.1, 31.9, 34.5, 35.1, 114.3, 119.6, 127.4, 127.6, 128.9, 131.2, 133.8, 142.4, 147.0, 147.8, 162.5. Spectral data were found to match those reported for this product. [6]

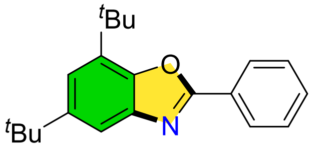

5,7-di-*tert*-butyl-2-(4-chlorophenyl)benzo[*d*]oxazole (7m).[7]
Purified by column chromatography on silica gel and eluted with ethyl acetate/petroleum ether (1:100). Isolated yield: 321 mg, 94%. Colorless liquid; ^{1}H NMR (400 MHz, $CDCl_3$) δ 1.43 (s, 9H), 1.58 (s, 9H), 7.35 (d, J = 1.8 Hz, 1H), 7.53 (d, J = 8.6 Hz, 2H), 7.68 (d, J = 1.8 Hz, 1H), 8.21 (d, J = 8.7 Hz, 2H); ^{13}C{^{1}H} NMR (100 MHz, $CDCl_3$) δ 30.1, 31.8, 34.5, 35.1, 114.3, 119.9, 126.0, 128.7, 129.3, 133.8, 137.5, 142.1, 147.0, 148.0, 161.5. Spectral data were found to match those reported for this product. [7]

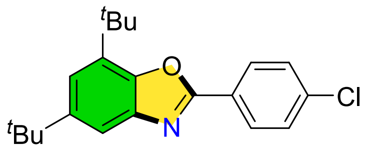

5,7-di-*tert*-butyl-2-(*p*-tolyl)benzo[*d*]oxazole (7n). [7]

Purified by column chromatography on silica gel and eluted with ethyl acetate/petroleum ether (1:100). Isolated yield: 293 mg, 91%. Colorless liquid; ^{1}H NMR (400 MHz, $CDCl_3$) δ 1.43 (s, 9H), 1.58 (s, 9H), 2.47 (s, 3H), 7.32 (d, *J* = 2.2 Hz, 1H), 7.36 (d, *J* = 8.0 Hz, 2H), 7.68 (d, *J* = 1.9 Hz, 1H), 8.17 (d, *J* = 8.2 Hz, 2H); ^{13}C{^{1}H} NMR (100 MHz, $CDCl_3$) δ 21.7, 30.1, 31.9, 34.5, 35.1, 114.1, 119.3, 124.8, 127.4, 129.6, 133.6, 141.7, 142.4, 146.9, 147.6, 162.8. Spectral data were found to match those reported for this product.[7]

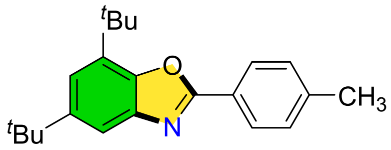

5,7-di-*tert*-butyl-2-(naphthalen-1-yl)benzo[*d*]oxazole (7o).[7]

Purified by column chromatography on silica gel and eluted with ethyl acetate/petroleum ether (1:100). Isolated yield: 297 mg, 83%. Colorless liquid; ^{1}H NMR (400 MHz, $CDCl_3$) δ 1.35 (s, 9H), 1.51 (s, 9H), 7.29 (d, *J* = 1.8 Hz, 1H), 7.50 – 7.57 (m, 2H), 7.63 (t, *J* = 8.4 Hz, 1H), 7.71 (d, *J* = 1.9 Hz, 1H), 7.86 (d, *J* = 7.3 Hz, 1H), 7.96 (d, *J* = 8.1 Hz, 1H), 8.34 (d, *J* = 7.4 Hz, 1H), 9.35 (d, *J* = 8.7 Hz, 1H); ^{13}C{^{1}H} NMR (100 MHz, $CDCl_3$) δ 30.1, 31.9, 34.6, 35.2, 114.4, 119.9, 123.9, 125.0, 126.3, 126.5, 127.9, 128.7, 129.2, 130.7, 132.1, 133.8, 134.0, 142.2, 146.4, 147.9, 162.4. Spectral data were found to match those reported for this product. [7]

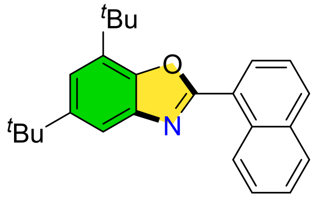

5,7-di-*tert*-butyl-2-(furan-2-yl)benzo[*d*]oxazole (7p).[9]

Purified by column chromatography on silica gel and eluted with ethyl acetate/petroleum ether (1:100). Isolated yield: 262 mg, 88%. Colorless liquid; ^{1}H NMR (400 MHz, $CDCl_3$) δ 1.41 (s, 9H), 1.56 (s, 9H), 6.63 (dd, *J* = 3.5, 1.7 Hz, 1H), 7.29 (d, *J* = 2.8 Hz, 1H), 7.34 (d, *J* = 1.8 Hz, 1H), 7.65 (dd, *J* = 1.9, 0.7 Hz, 1H), 7.68 (dd, *J* = 1.7, 0.8 Hz, 1H); ^{13}C{^{1}H} NMR (100 MHz, $CDCl_3$) δ 30.0, 31.8, 34.5, 35.1, 112.1, 113.8, 114.3, 119.8, 133.8, 141.7, 142.9, 145.5, 146.4, 148.1, 154.9. Spectral data were found to match those reported for this product.[9]

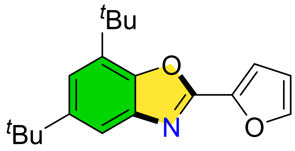

5-(*tert*-butyl)-2-(4-chlorophenyl)benzo[*d*]oxazole (7q). [11]

Purified by column chromatography on silica gel and eluted with ethyl acetate/petroleum ether (1:100). Isolated yield: 231 mg, 81%. Colorless liquid; ^{1}H NMR (300 MHz, $CDCl_3$) δ 1.33 (s, 9H), 7.36 (dd, *J* = 8.5, 1.7 Hz, 1H), 7.42 (d, *J* = 8.6 Hz, 2H), 7.53 (s, 1H), 7.61 (d, *J* = 8.4 Hz, 1H), 8.10 (d, *J* = 8.6 Hz, 2H); ^{13}C{^{1}H} NMR (75 MHz, $CDCl_3$) δ 31.7, 35.2, 107.4, 119.2, 122.5, 125.9, 128.7, 129.3, 137.5, 139.6, 149.6, 151.0, 161.9. Spectral data were found to match those reported for this product.[11]

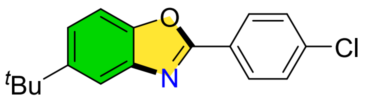

5,7-di-*tert*-butyl-2-(4-chlorophenyl)-4-methylbenzo[*d*]oxazole (7r).[12]

Purified by column chromatography on silica gel and eluted with ethyl acetate/petroleum ether (1:100). Isolated yield: 285 mg, 80%. Colorless liquid; ^{1}H NMR (400 MHz, $CDCl_3$) δ 1.52 (s, 9H), 1.54 (s, 9H), 2.86 (s, 3H), 7.34 (s, 1H), 7.51 (d, *J* = 8.6 Hz, 2H), 8.22 (d, *J* = 8.6 Hz, 2H); $^{13}C\{^1H\}$ NMR (100 MHz, $CDCl_3$) δ 16.3, 30.1, 31.4, 34.3, 36.3, 120.3, 126.2, 126.4, 128.7, 129.1, 130.4, 137.1, 143.7, 146.4, 160.5. Spectral data were found to match those reported for this product.[12]

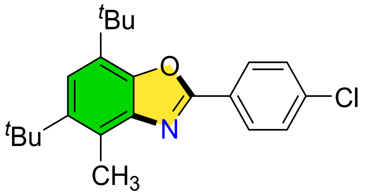

5,7-di-*tert*-butylbenzo[*d*]oxazole (7s) [*Synthesized from glycine*].[7]

Purified by column chromatography on silica gel and eluted with ethyl acetate/petroleum ether (1:100). Isolated yield: 185 mg, 80%. Colorless liquid; ^{1}H NMR (400 MHz, DMSO-d_6) δ 1.31 (s, 9H), 1.41 (s, 9H), 7.30 (d, *J* = 2.2 Hz, 1H), 7.57 (d, *J* = 2.6 Hz, 1H), 8.62 (s, 1H); $^{13}C\{^1H\}$ NMR (100 MHz, DMSO-d_6) δ 30.0, 32.0, 34.4, 35.1, 114.5, 119.9, 134.2, 140.3, 146.0, 147.9, 154.1. Spectral data were found to match those reported for this product.[7]

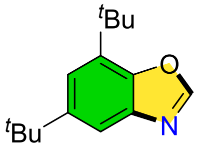

(*R*)-2-(*sec*-butyl)-5,7-di-*tert*-butylbenzo[*d*]oxazole (7t) [*Synthesized from L-isoleucine*].[11]

Purified by column chromatography on silica gel and eluted with ethyl acetate/petroleum ether (1:100). Isolated yield: 273 mg, 95%. Colorless liquid; ^{1}H NMR (400 MHz, $CDCl_3$) δ 1.00 (t, *J* = 7.4 Hz, 3H), 1.40 (s, 9H), 1.46 (d, *J* = 7.0 Hz, 3H), 1.50 (s, 9H), 1.76 – 1.84 (m, 1H), 1.93 – 2.00 (m, 1H), 3.04 – 3.13 (m, 1H), 7.27 (d, *J* = 2.8 Hz, 1H), 7.60 (d, *J* = 2.1 Hz, 1H); $^{13}C\{^1H\}$ NMR (100 MHz, $CDCl_3$) δ 11.7, 18.0, 28.2, 29.9, 31.9, 34.4, 35.0, 35.7, 113.9, 118.6, 133.5, 141.4, 146.9, 147.1, 170.1. Spectral data were found to match those reported for this product.[11]

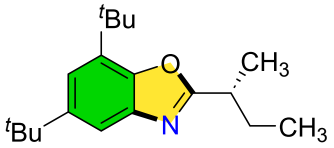

5,7-di-*tert*-butyl-2-isobutylbenzo[*d*]oxazole (7u) [*Synthesized from L-leucine*].[11]

Purified by column chromatography on silica gel and eluted with ethyl acetate/petroleum ether (1:100). Isolated yield: 267 mg, 93%. Colorless liquid; ^{1}H NMR (400 MHz, $CDCl_3$) δ 1.40 (s, 9H), 1.50 (s, 9H), 2.25-7.38 (m, 1H), 2.85 (d, *J* = 7.0 Hz, 2H), 7.27 (d, *J* = 2.2 Hz, 1H), 7.58 (d, *J* = 3.0 Hz, 1H); $^{13}C\{^1H\}$ NMR (100 MHz, $CDCl_3$) δ 22.5, 27.6, 29.9, 31.9, 34.4, 35.0, 37.6, 113.8, 118.7, 133.5, 141.6, 147.0, 147.1, 166.0. Spectral data were found to match those reported for this product.[11]

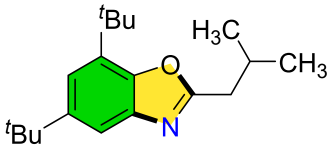

1,2-bis(5,7-di-*tert*-butylbenzo[*d*]oxazol-2-yl)ethane (7v) [*Synthesized from L-arginine*].[11]

Purified by column chromatography on silica gel and eluted with ethyl acetate/petroleum ether (1:100). Isolated yield: 362 mg, 74%. Colorless liquid; ^{1}H NMR (400 MHz, $CDCl_3$) δ 1.39 (s, 18H), 1.43 (s, 18H), 3.59 (s, 4H), 7.27 (d, J = 1.7 Hz, 2H), 7.57 (d, J = 1.2 Hz, 2H); ^{13}C{^{1}H} NMR (100 MHz, $CDCl_3$) δ 26.0, 29.9, 31.9, 34.4, 35.0, 113.9, 119.1, 133.6, 141.4, 147.2, 147.4, 164.3. Spectral data were found to match those reported for this product.[11]

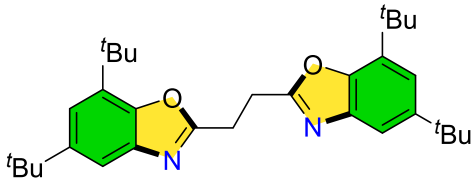

References

[1] J. Jacquet, P. Chaumont, G. Gontard, M. Orio, H. Vezin, S. Blanchard, M. Desage-El Murr, L. Fensterbank, “C−N Bond Formation from a Masked High-Valent Copper Complex Stabilized by Redox Non-Innocent Ligands” *Angew. Chemie* **2016**, *128*, 10870–10874.

[2] S. Fuse, H. Tago, M. M. Maitani, Y. Wada, T. Takahashi, “Sequential coupling approach to the synthesis of nickel(II) complexes with N-aryl-2-amino phenolates” *ACS Comb. Sci.* **2012**, *14*, 545–550.

[3] M. Nasibipour, E. Safaei, G. Wrzeszcz, A. Wojtczak, “Tuning of the redox potential and catalytic activity of a new Cu(ii) complex by: O -iminobenzosemiquinone as an electron-reservoir ligand” *New J. Chem.* **2020**, *44*, 4426–4439.

[4] E. Bill, E. Bothe, P. Chaudhuri, K. Chlopek, D. Herebian, S. Kokatam, K. Ray, T. Weyhermüller, F. Neese, K. Wieghardt, “Molecular and electronic structure of four- and five-coordinate cobalt complexes containing two o-phenylenediamine- or two o-aminophenol-type ligands at various oxidation levels: An experimental, density functional, and correlated ab initio study” *Chem. - A Eur. J.* **2005**, *11*, 204–224.

[5] M. Hedoyatullah, F. Thevenet, “Oxydation d’Amines Aromatiques Primaires en Présence du Radical Tritertiobutyl-2,4,6 Phénoxyle” *Bull. des Sociétés Chim. Belges* **1987**, *96*, 311–323.

[6] D. L. J. Broere, L. L. Metz, B. De Bruin, J. N. H. Reek, M. A. Siegler, J. I. Van Der Vlugt, “Redox-active ligand-induced homolytic bond activation” *Angew. Chemie - Int. Ed.* **2015**, *54*, 1516–1520.

[7] X. Chen, F. Ji, Y. Zhao, Y. Liu, Y. Zhou, T. Chen, S. F. Yin, “Copper-Catalyzed Aerobic Oxidative C(aryl)-OH Bond Functionalization of Catechols with Amines Affording Benzoxazoles” *Adv. Synth. Catal.* **2015**, *357*, 2924–2930.

[8] L. Liu, L. W. Qian, S. Wu, J. Dong, Q. Xu, Y. Zhou, S. F. Yin, “Selective Aerobic C-H Amination of Phenols with Primary Amines over Copper toward Benzoxazoles” *Org. Lett.* **2017**, *19*, 2849–2852.

[9] X. Meng, Y. Wang, Y. Wang, B. Chen, Z. Jing, G. Chen, P. Zhao, “OMS-2-Supported Cu Hydroxide-Catalyzed Benzoxazoles Synthesis from Catechols and Amines via Domino Oxidation Process at Room Temperature” *J. Org. Chem.* **2017**, *82*, 6922–6931.

[10] H. Sharghi, M. Aali Hosseini, J. Aboonajmi, M. Aberi, “Use of Vitamin B12as a Nontoxic and Natural Catalyst for the Synthesis of Benzoxazoles via Catechols and Primary Amines in Water under Aerobic Oxidation” *ACS Sustain. Chem. Eng.* **2021**, *9*, 11163–11170.

[11] H. Sharghi, J. Aboonajmi, M. Aberi, M. Shekouhy, “Amino Acids: Nontoxic and Cheap Alternatives for Amines for the Synthesis of Benzoxazoles through the Oxidative Functionalization of Catechols” *Adv. Synth. Catal.* **2020**, *362*, 1064–1083.

[12] M. Mohammadi, J. Aboonajmi, F. Panahi, M. Sasanipour, H. Sharghi, “Zirconium-catalyzed one-pot synthesis of benzoxazoles using reaction of catechols, aldehydes and ammonium acetate” *Sci. Rep.* **2024**, *14*, 25973.

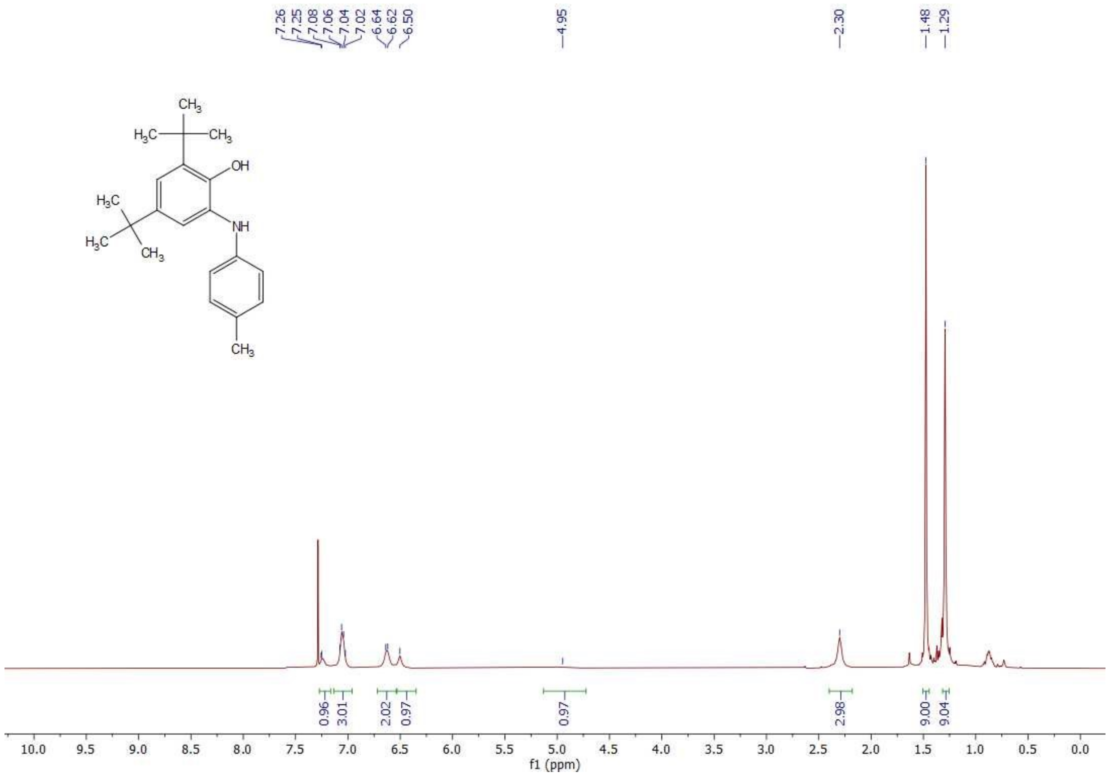

Figure S1. ^{1}H (400 MHz, $CDCl_3$), compound **3a**

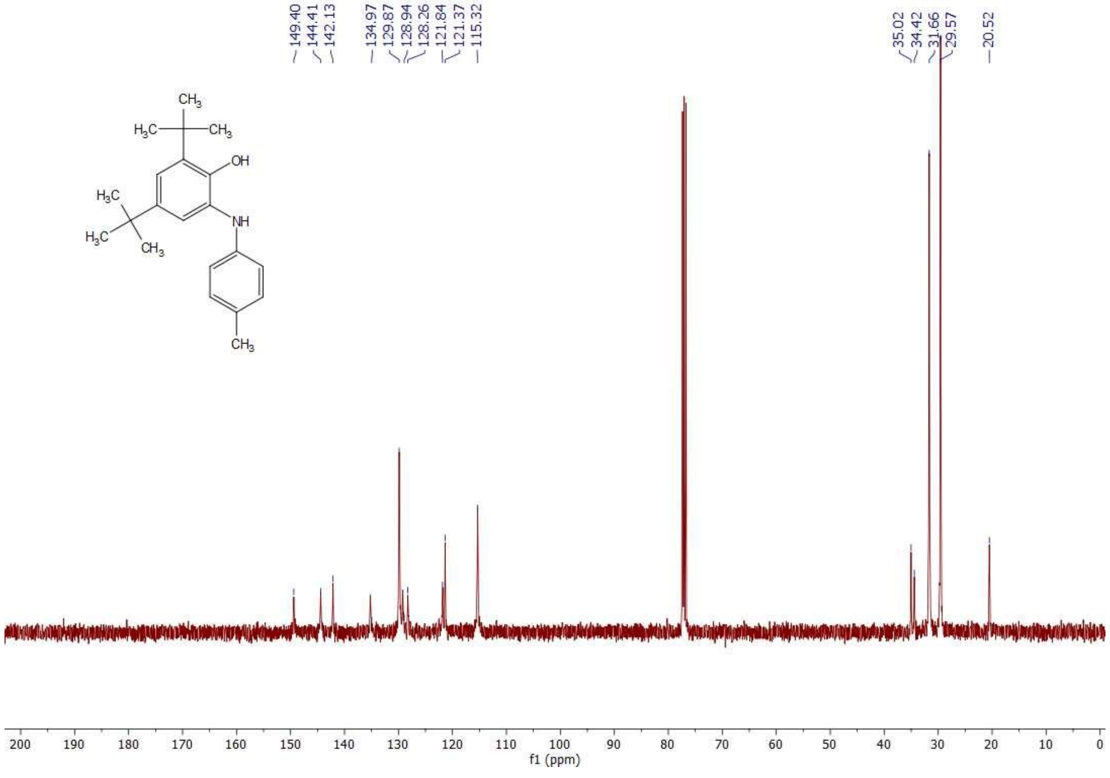

Figure S2. ^{13}C (100 MHz, $CDCl_3$), compound **3a**

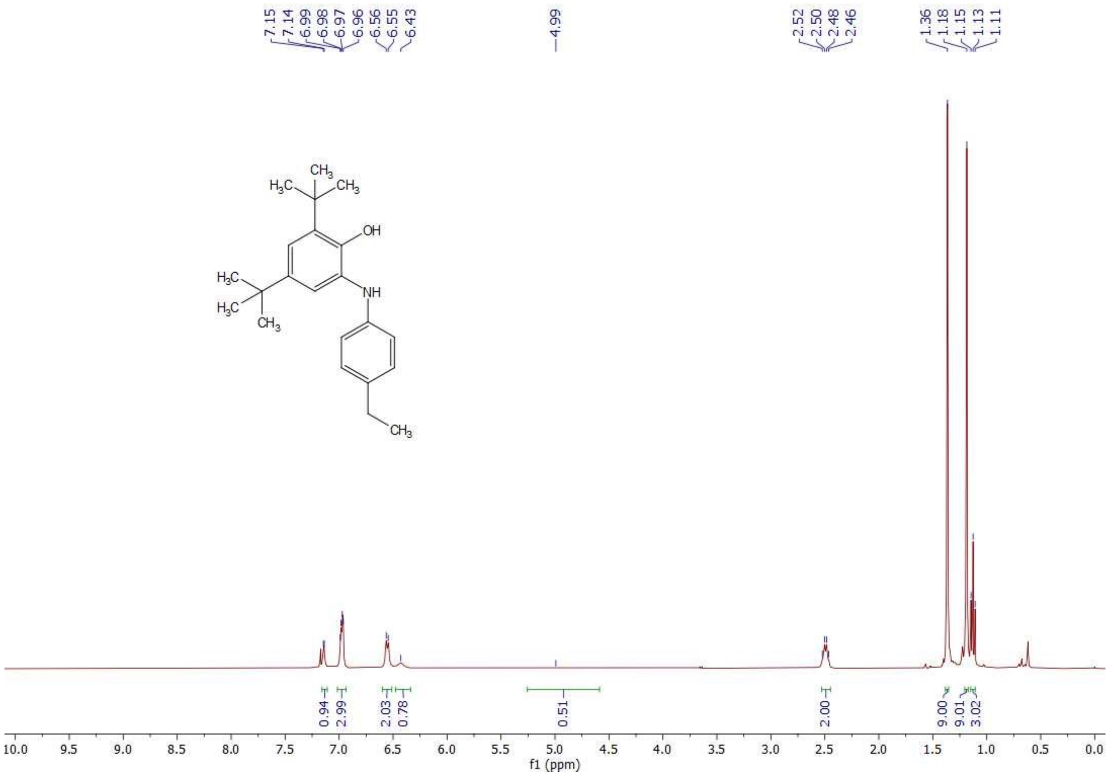

Figure S3. ^{1}H (400 MHz, $CDCl_3$), compound **3b**

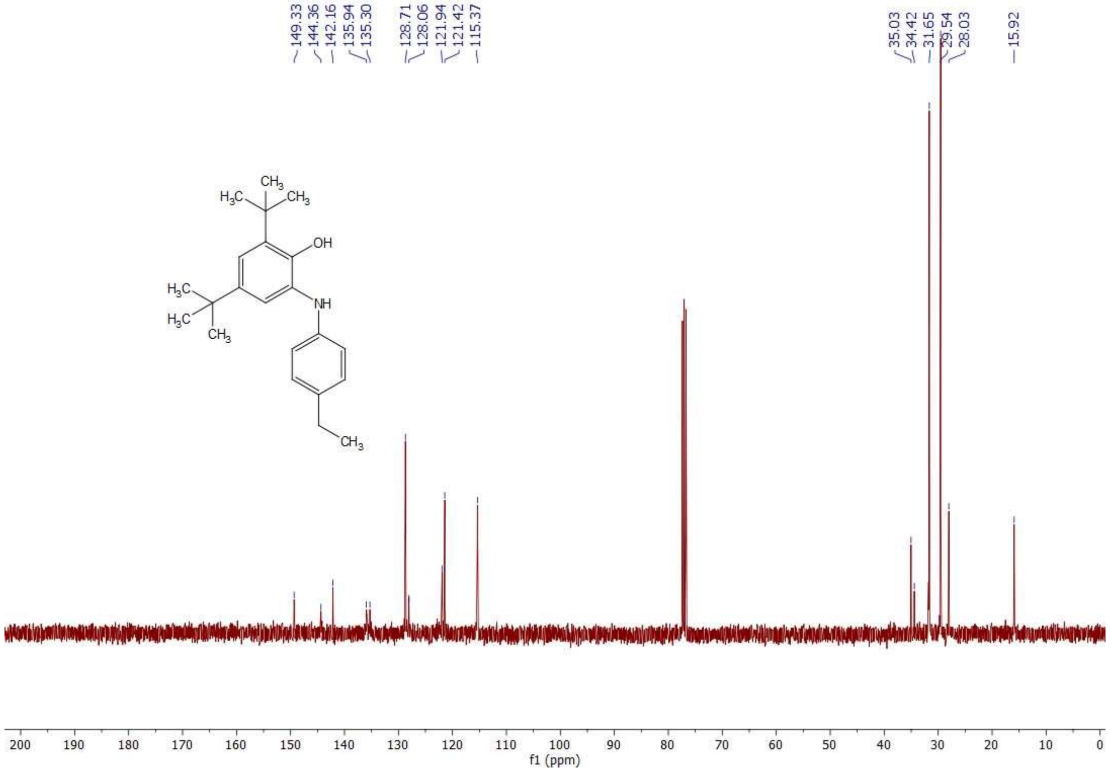

Figure S4. ^{13}C (100 MHz, $CDCl_3$), compound **3b**

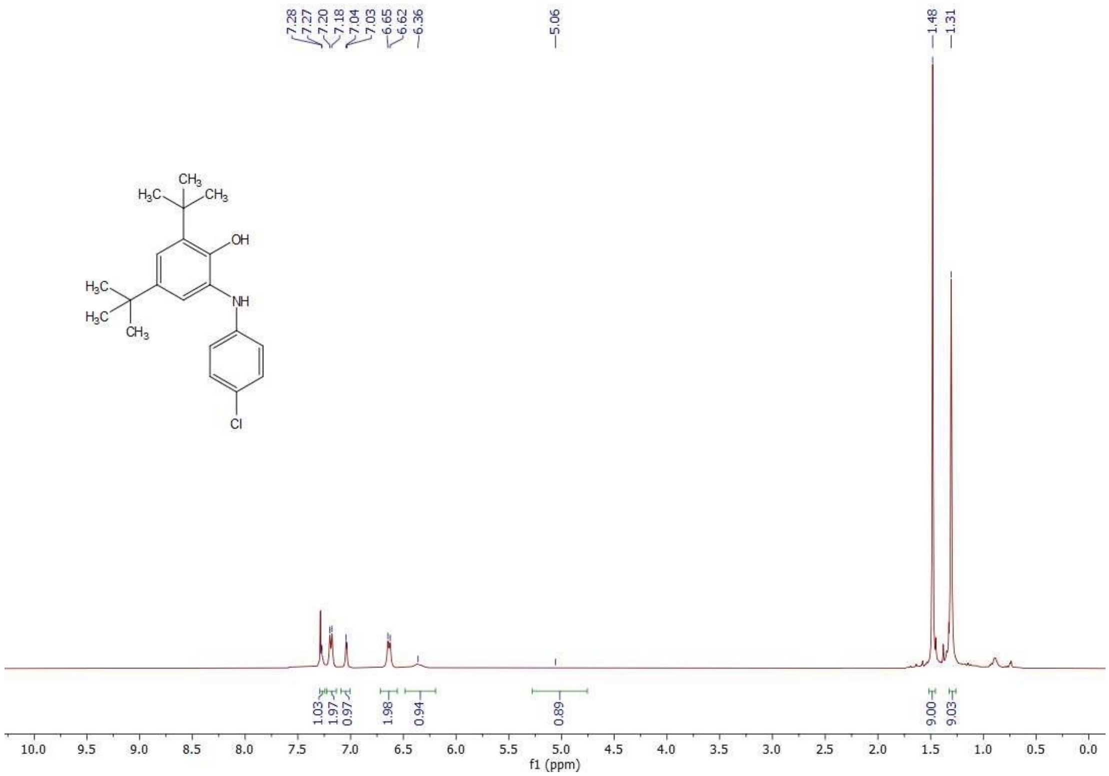

Figure S5. ^{1}H (400 MHz, $CDCl_3$), compound **3c**

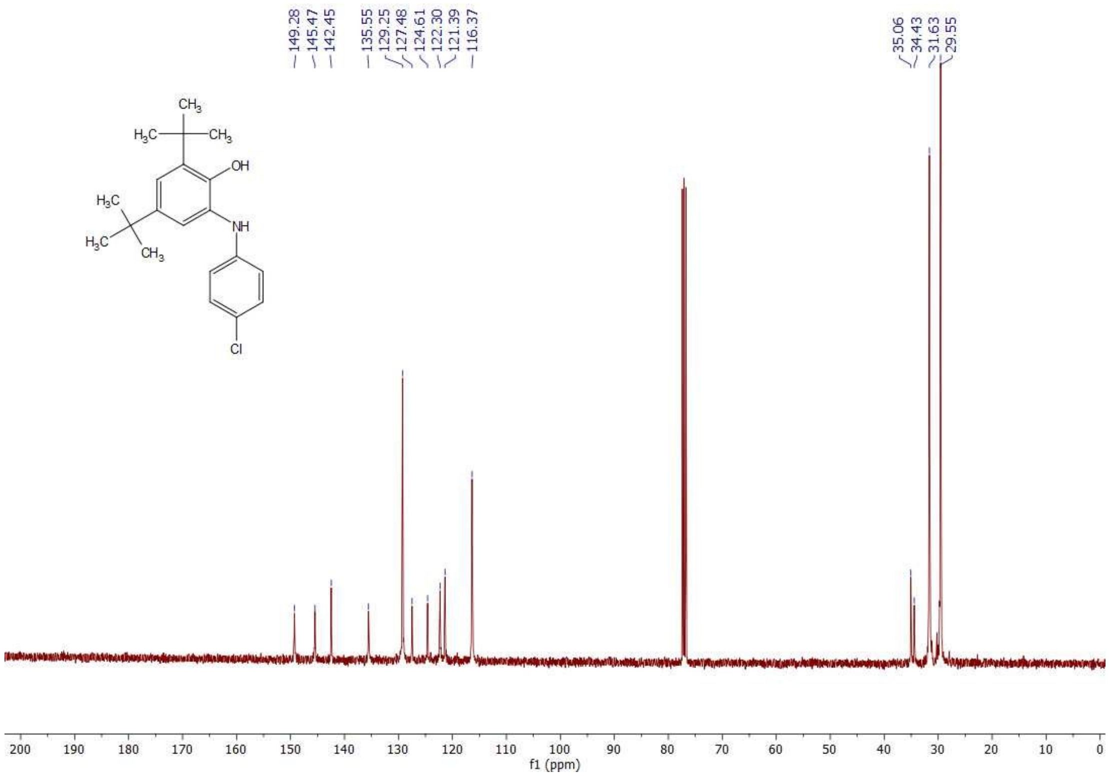

Figure S6. ^{13}C (100 MHz, $CDCl_3$), compound **3c**

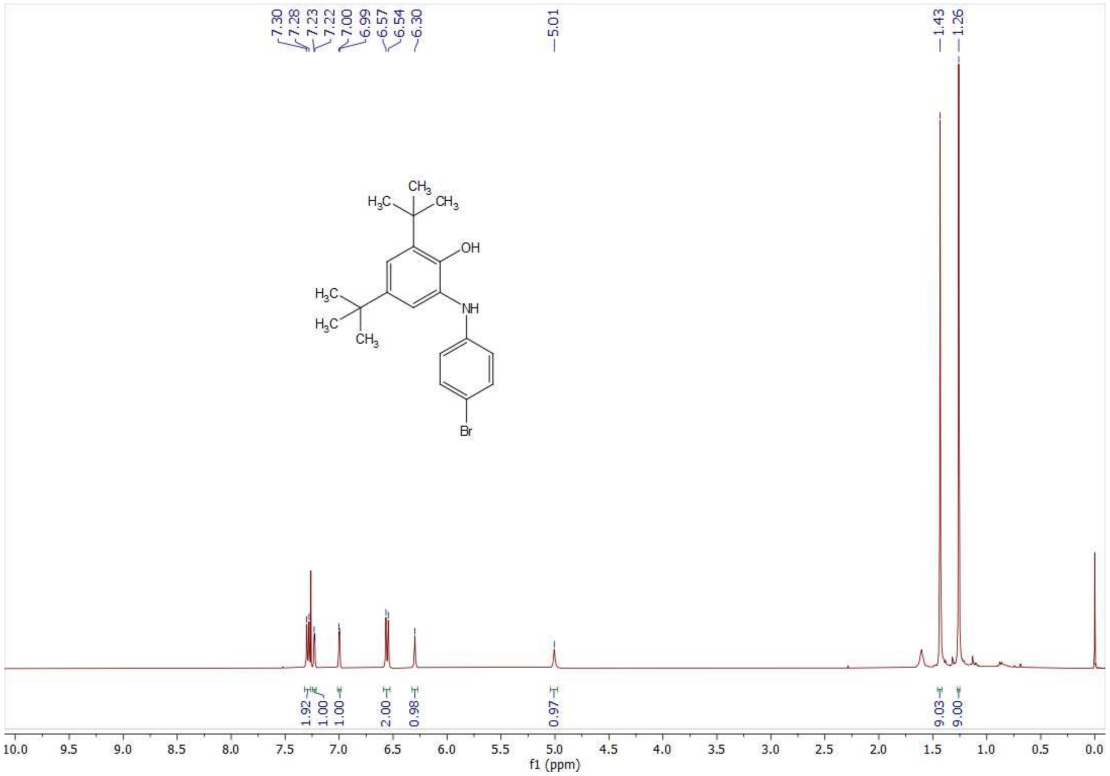

Figure S7. ^{1}H (400 MHz, $CDCl_3$), compound **3d**

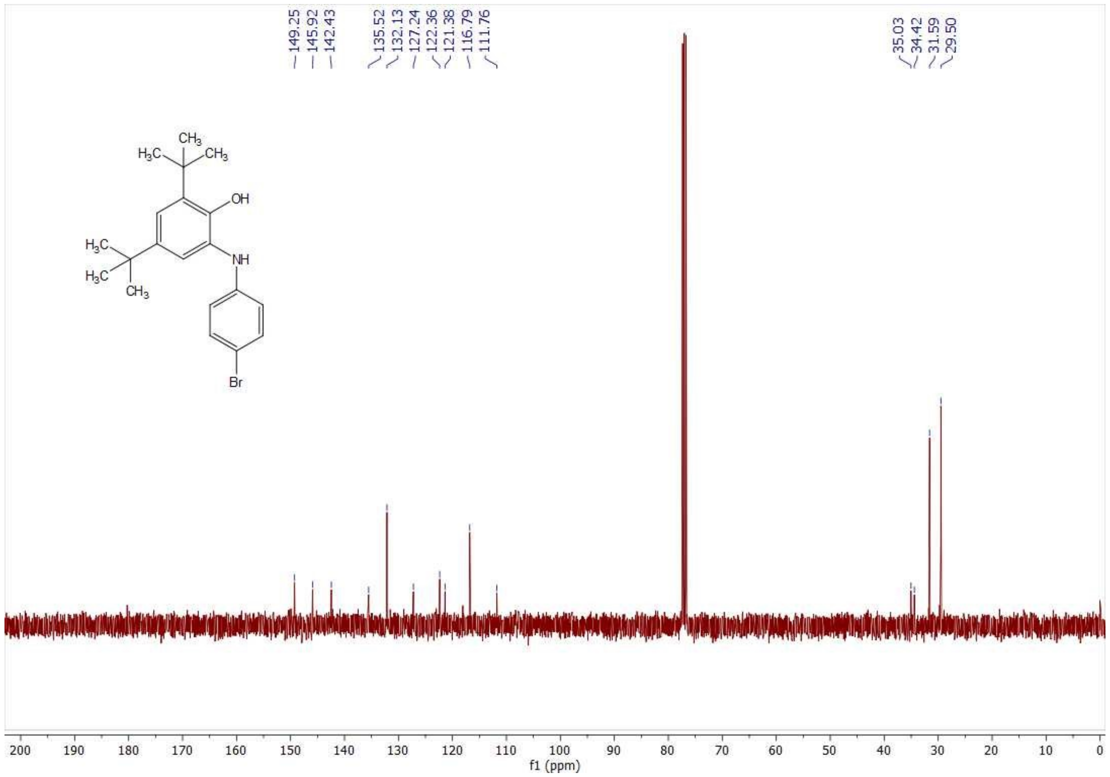

Figure S8. ^{13}C (100 MHz, $CDCl_3$), compound **3d**

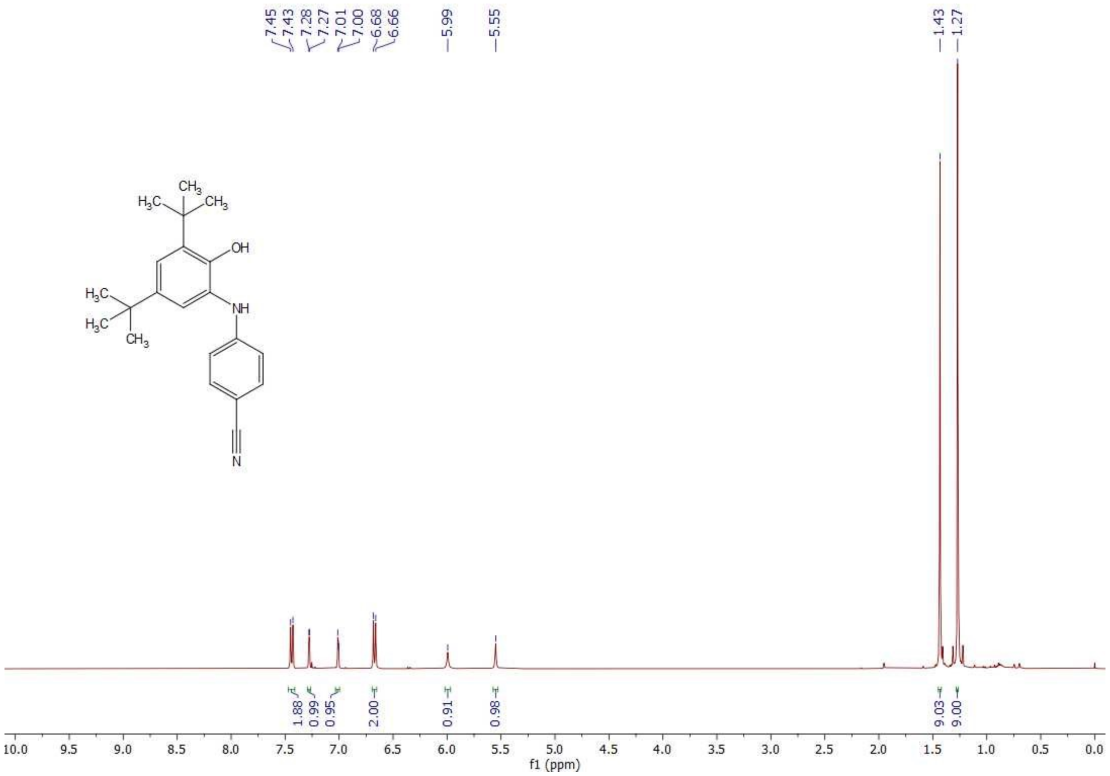

Figure S9. ^{1}H (400 MHz, $CDCl_3$), compound **3e**

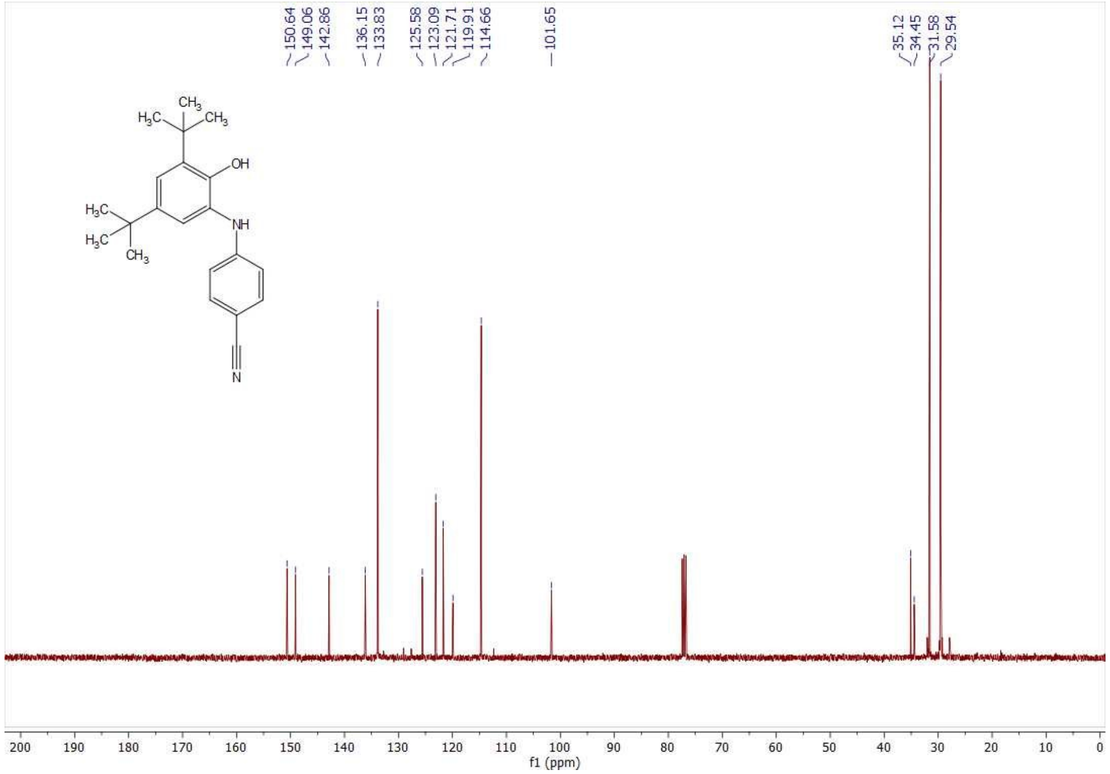

Figure S10. ^{13}C (100 MHz, $CDCl_3$), compound **3e**

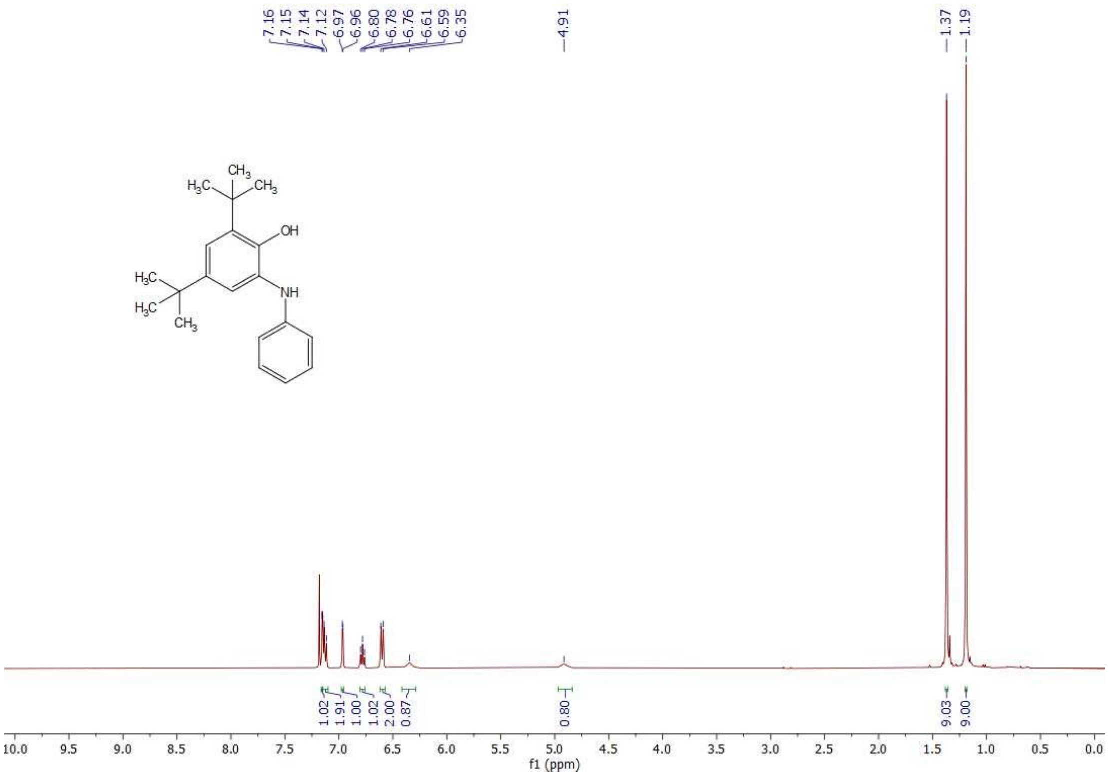

Figure S11. ^{1}H (400 MHz, $CDCl_3$), compound **3f**

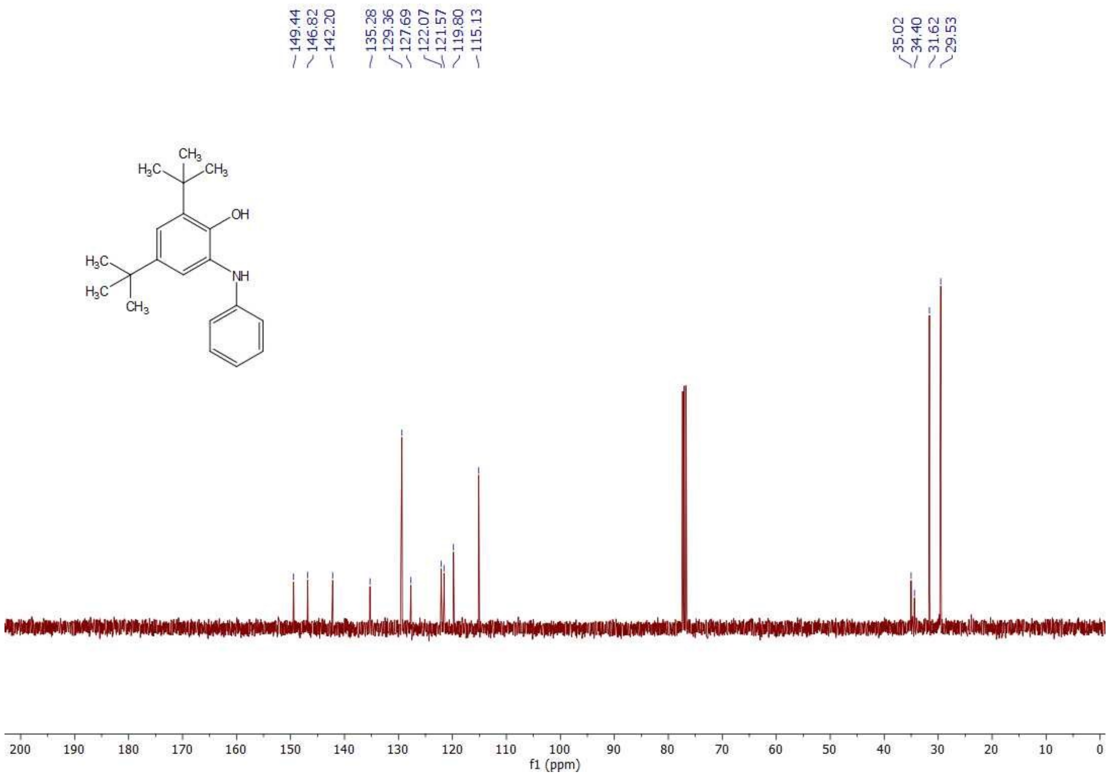

Figure S12. ^{13}C (100 MHz, $CDCl_3$), compound **3f**

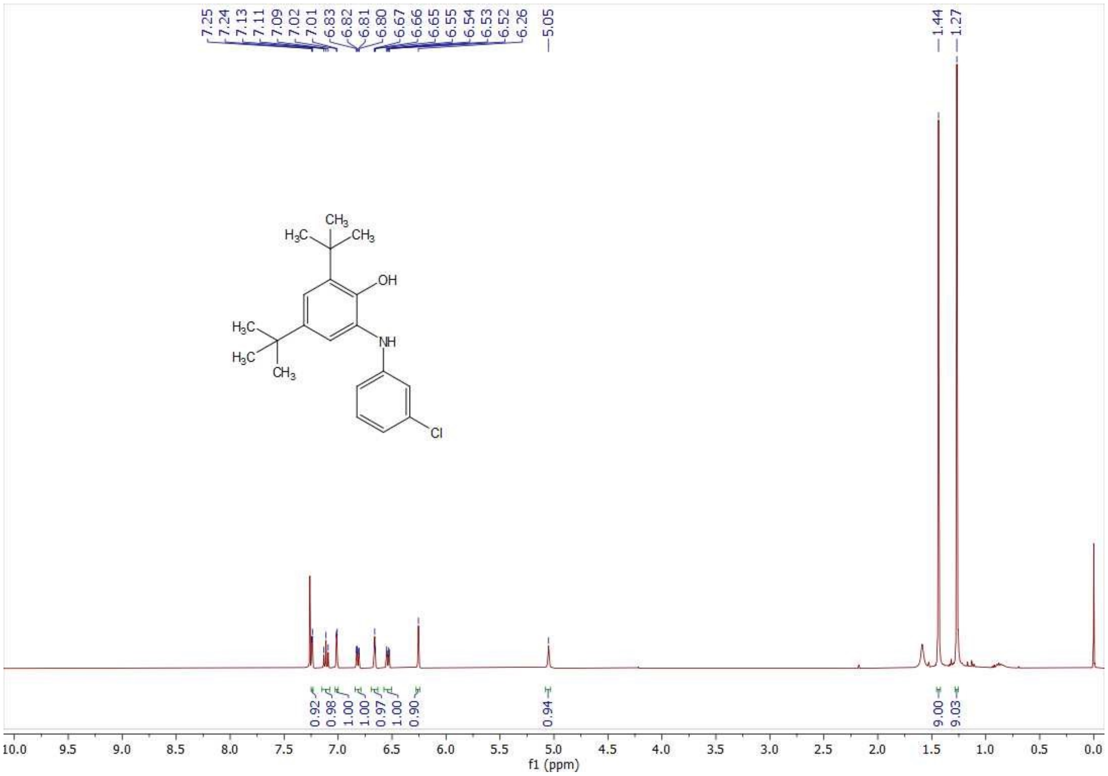

Figure S13. ^{1}H (400 MHz, $CDCl_3$), compound **3g**

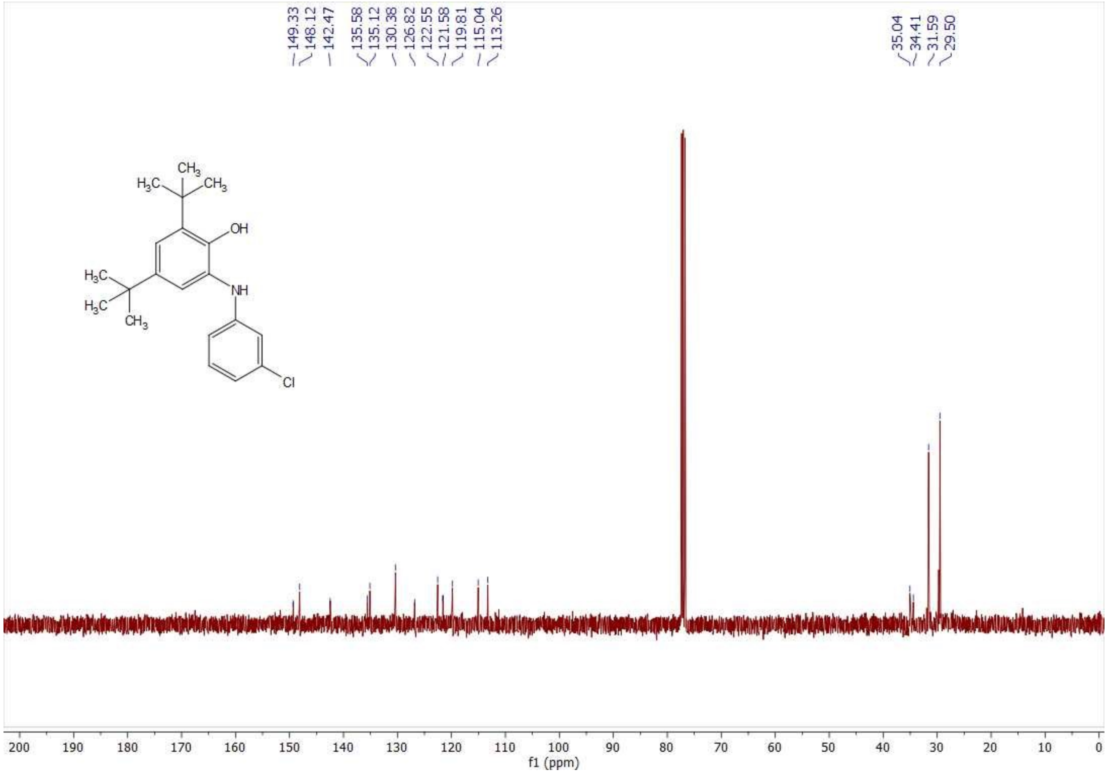

Figure S14. ^{13}C (100 MHz, $CDCl_3$), compound **3g**

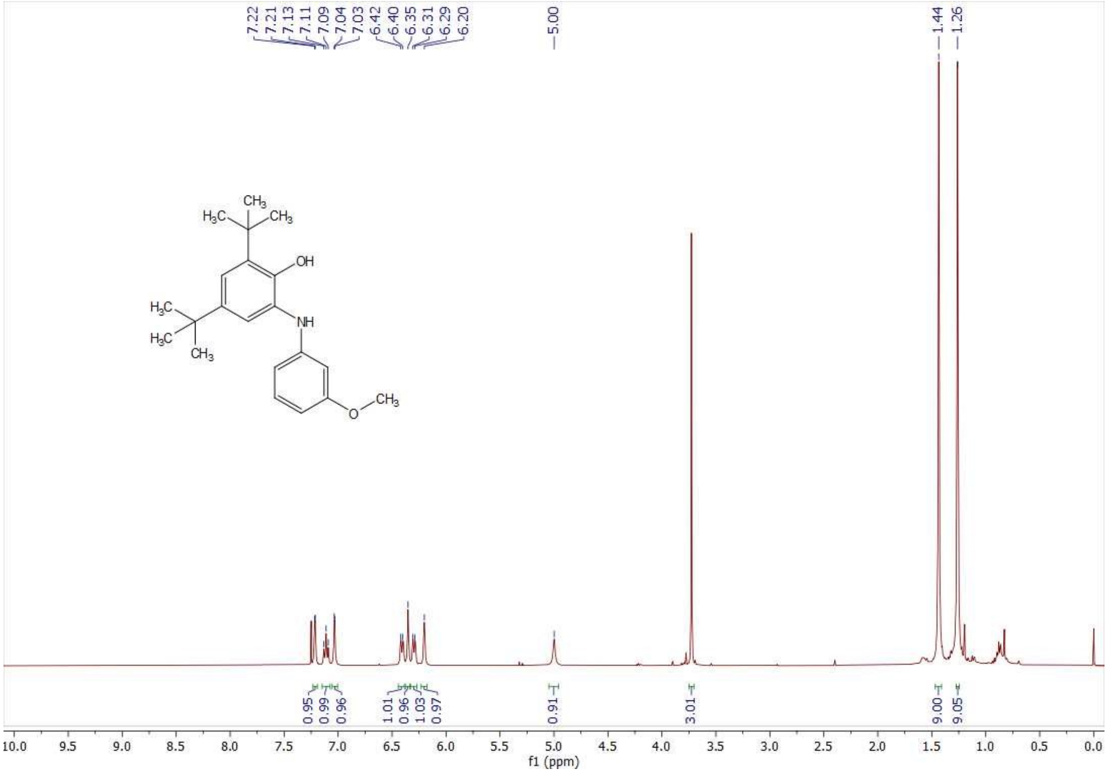

Figure S15. ^{1}H (400 MHz, $CDCl_3$), compound **3h**

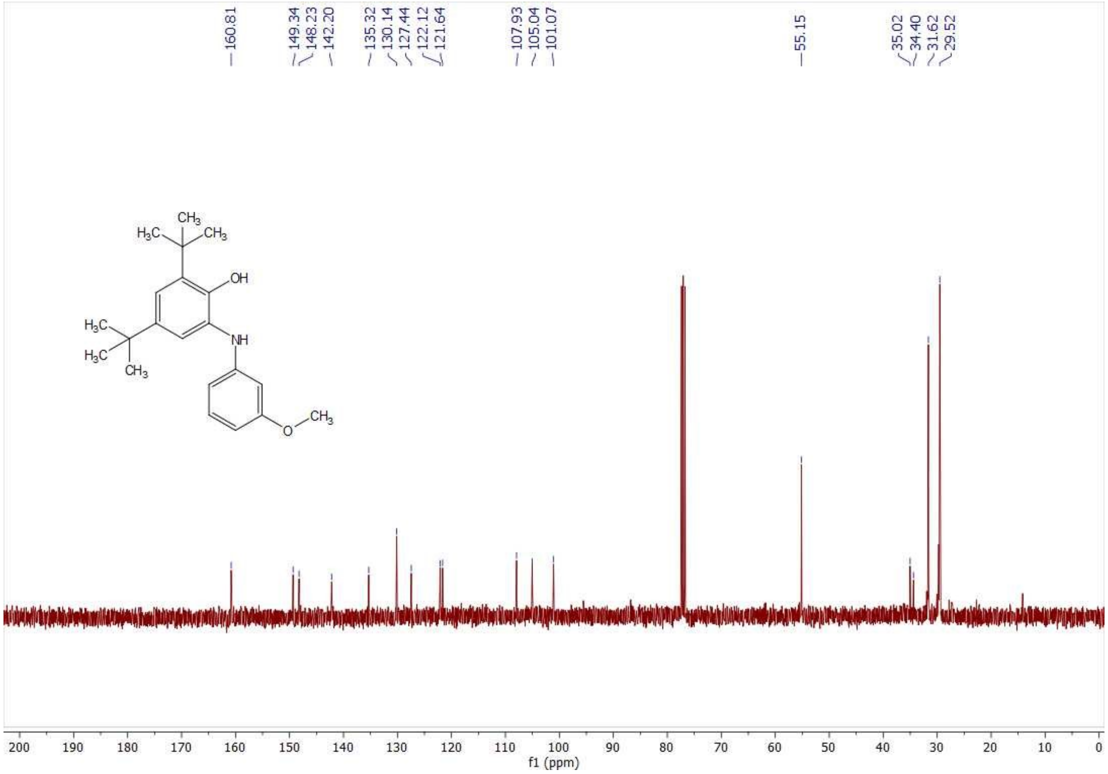

Figure S16. ^{13}C (100 MHz, $CDCl_3$), compound **3h**

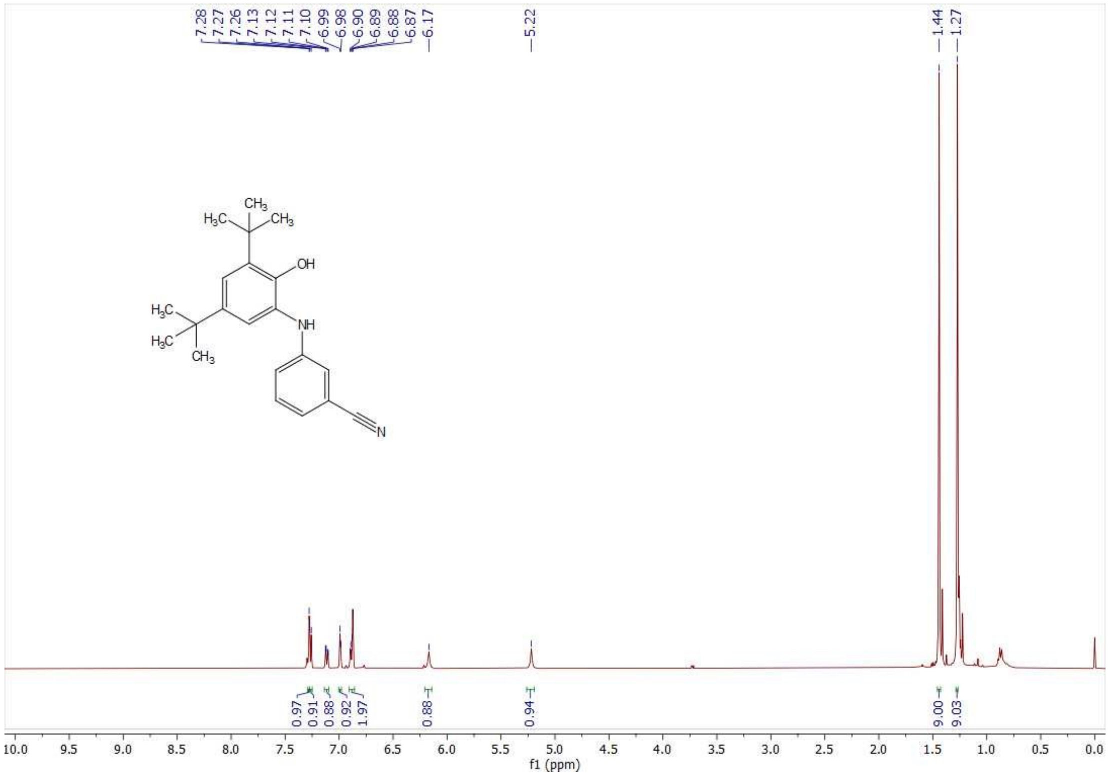

Figure S17. ^{1}H (400 MHz, $CDCl_3$), compound **3i**

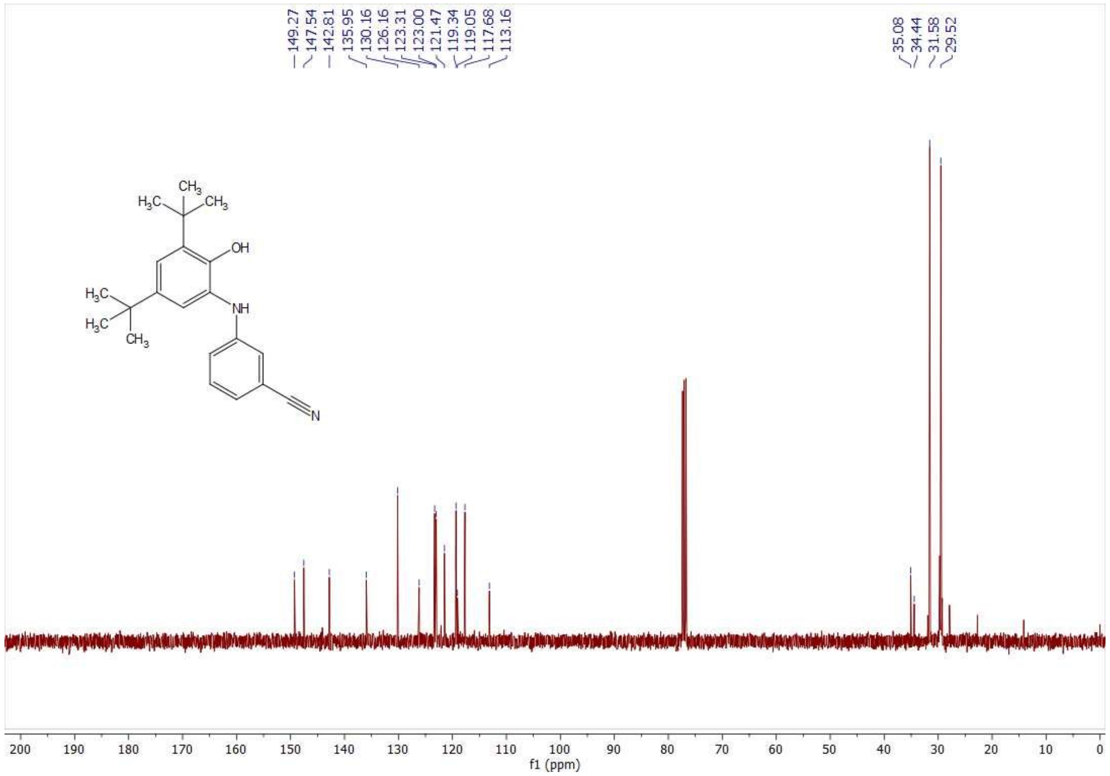

Figure S18. ^{13}C (100 MHz, $CDCl_3$), compound **3i**

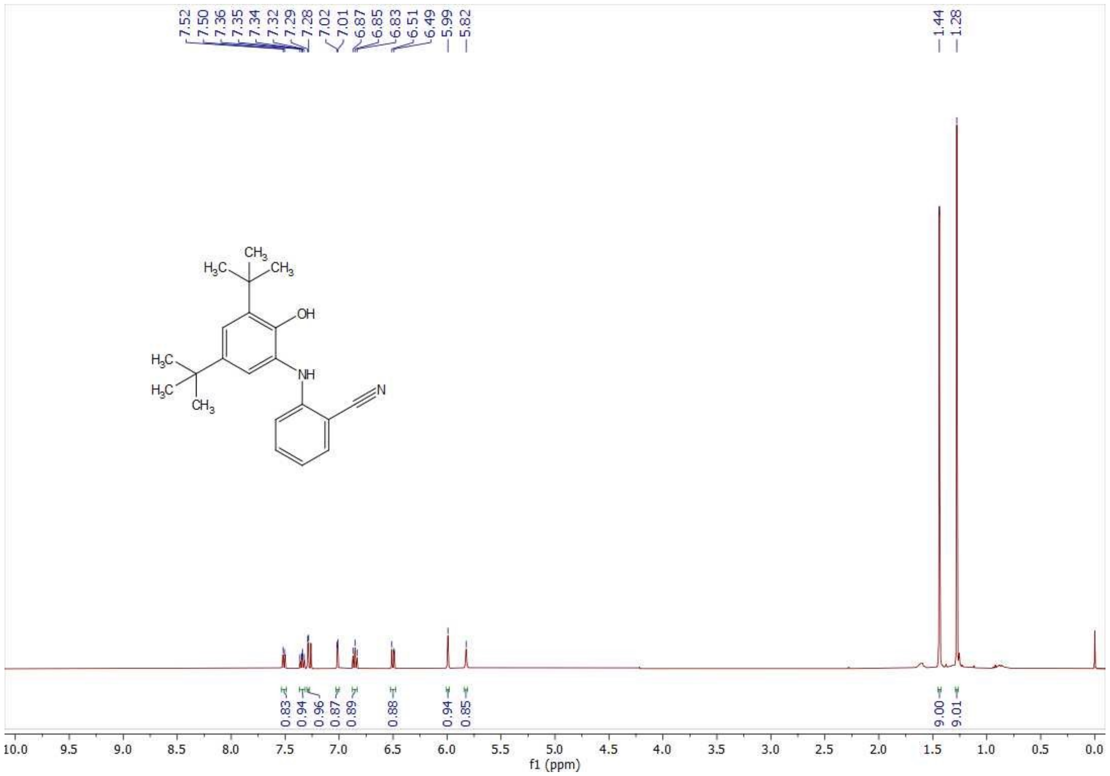

Figure S19. ^{1}H (400 MHz, $CDCl_3$), compound **3j**

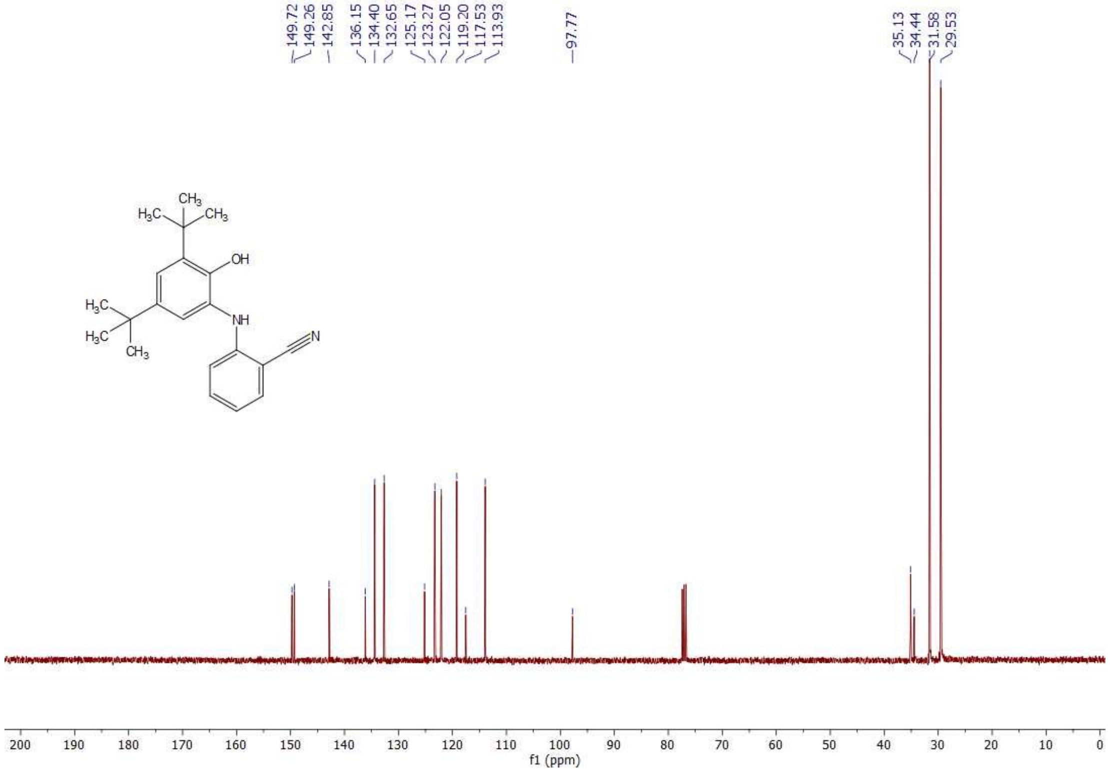

Figure S20. ^{13}C (100 MHz, $CDCl_3$), compound **3j**

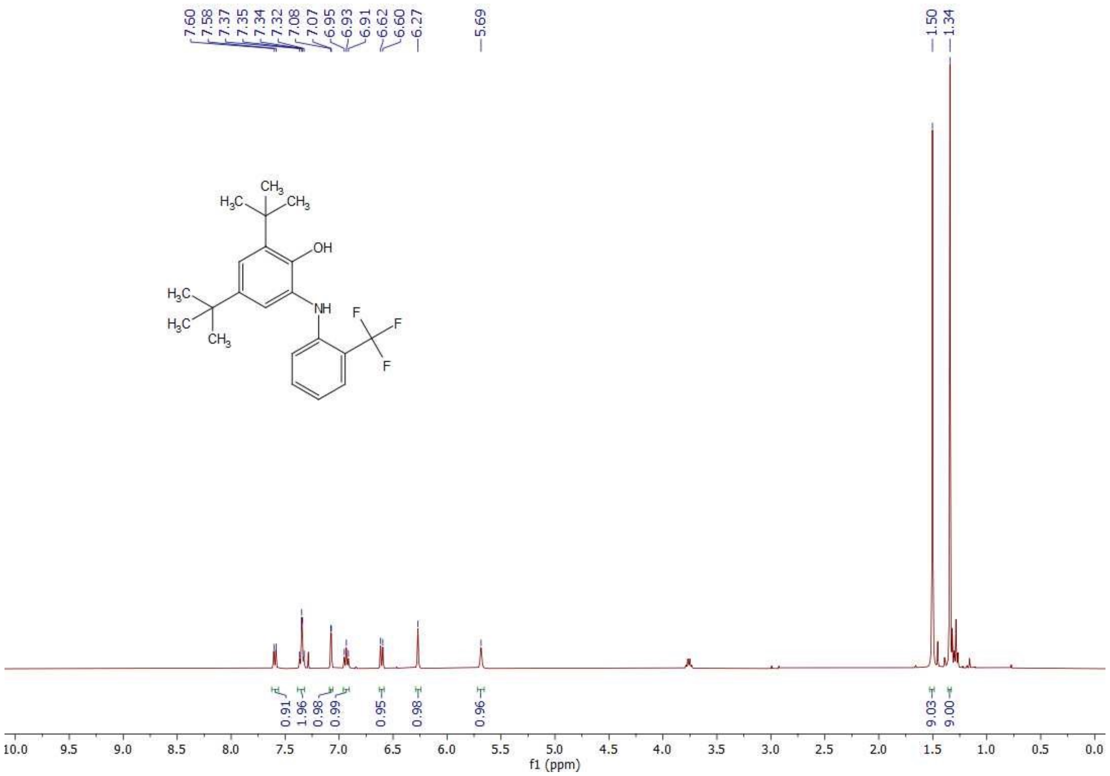

Figure S21. ^{1}H (400 MHz, $CDCl_3$), compound **3k**

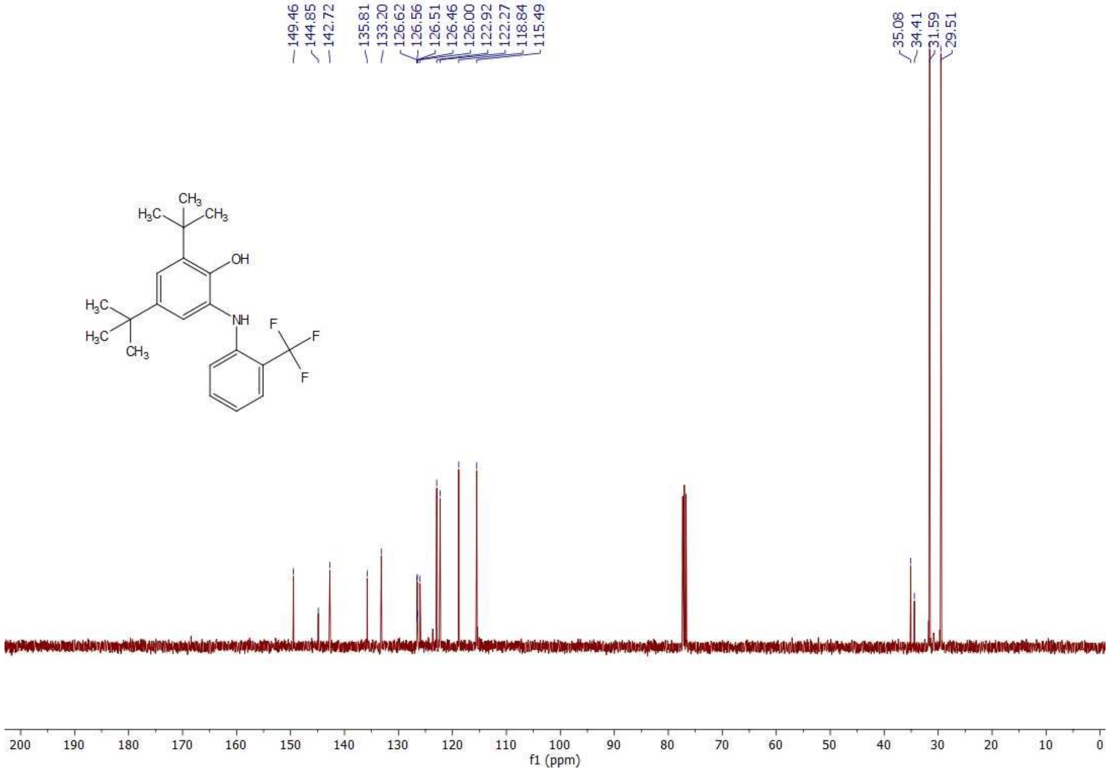

Figure S22. ^{13}C (100 MHz, $CDCl_3$), compound **3k**

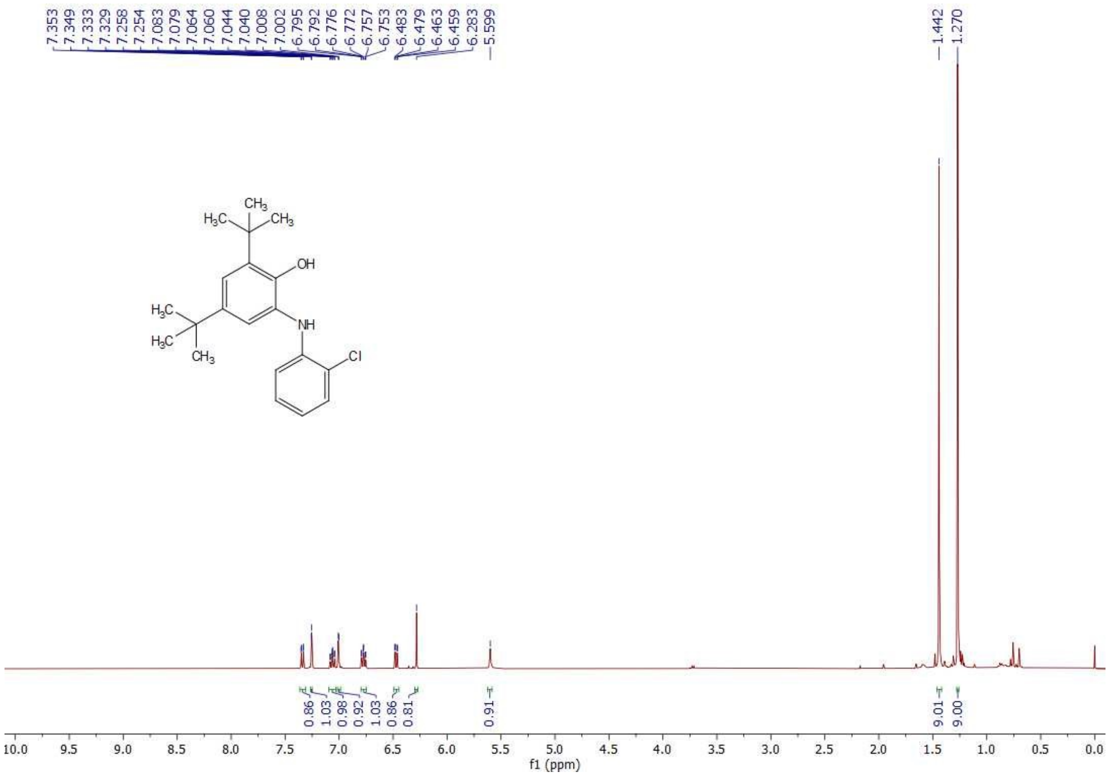

Figure S23. ^{1}H (400 MHz, $CDCl_3$), compound **3l**

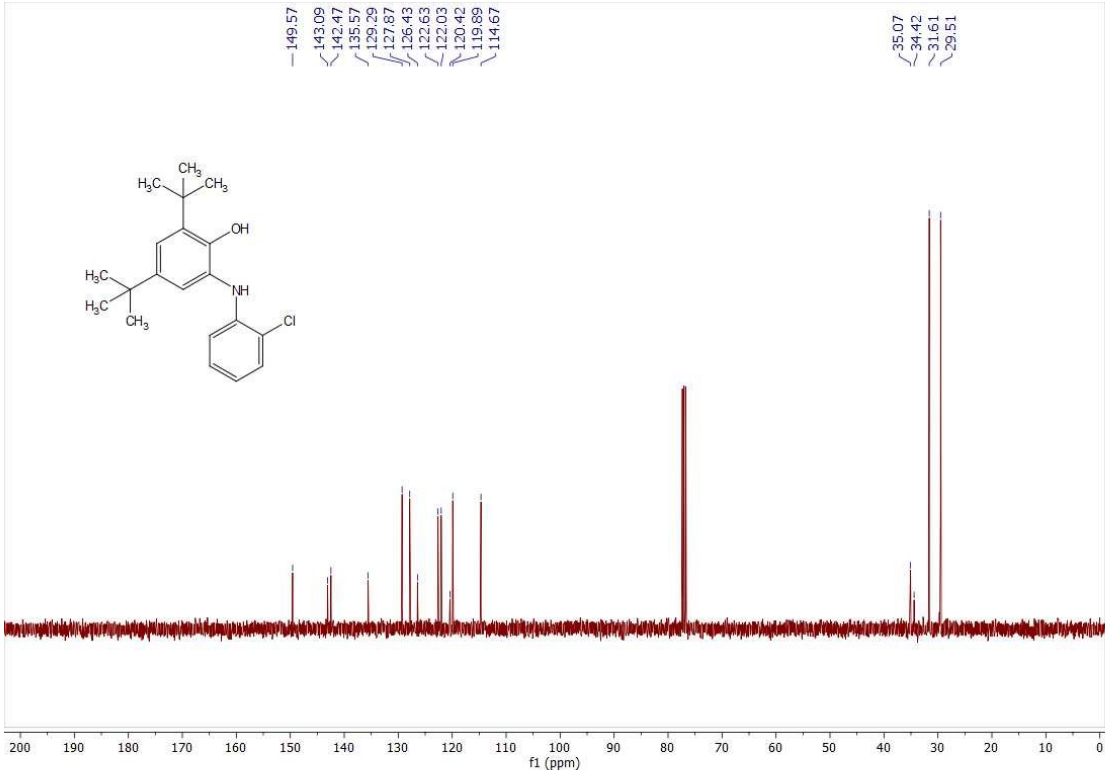

Figure S24. ^{13}C (100 MHz, $CDCl_3$), compound **3l**

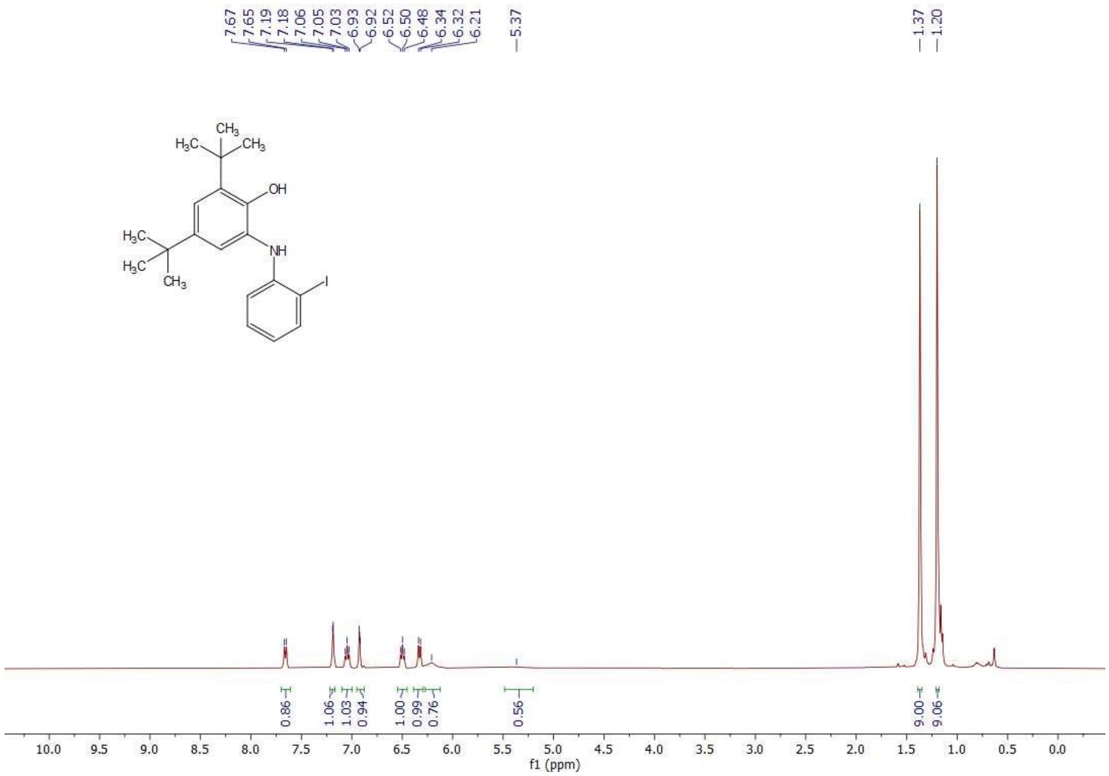

Figure S25. ^{1}H (400 MHz, $CDCl_3$), compound **3m**

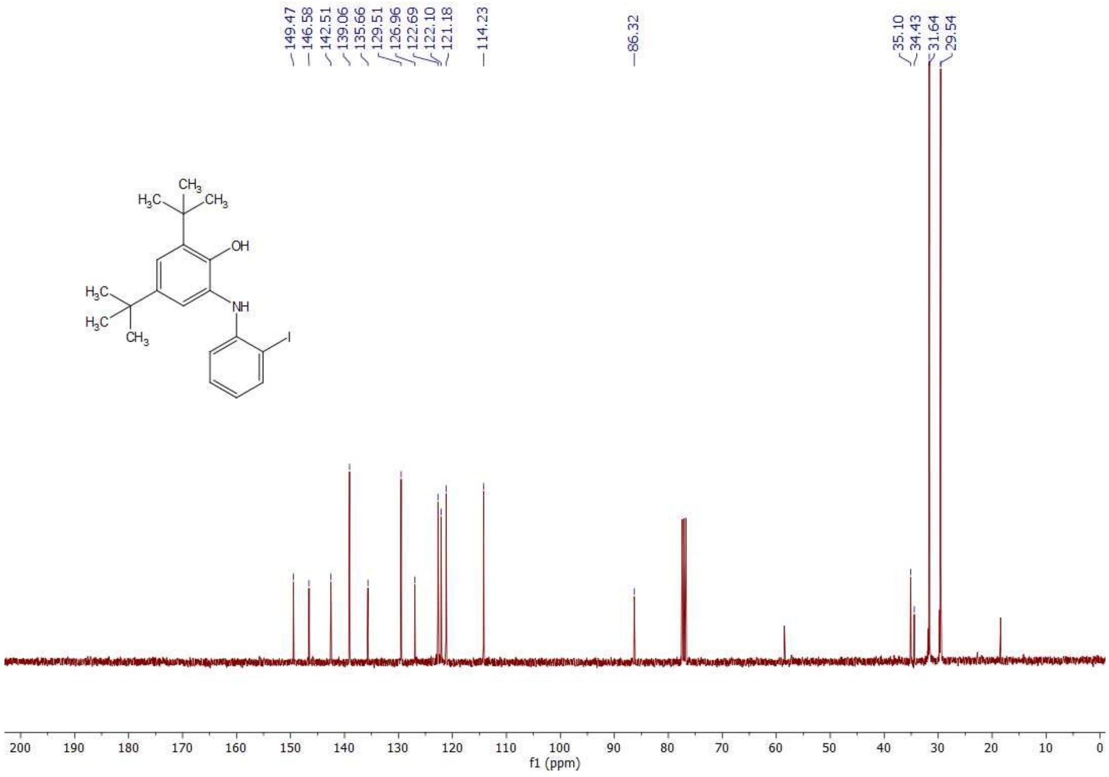

Figure S26. ^{13}C (100 MHz, $CDCl_3$), compound **3m**

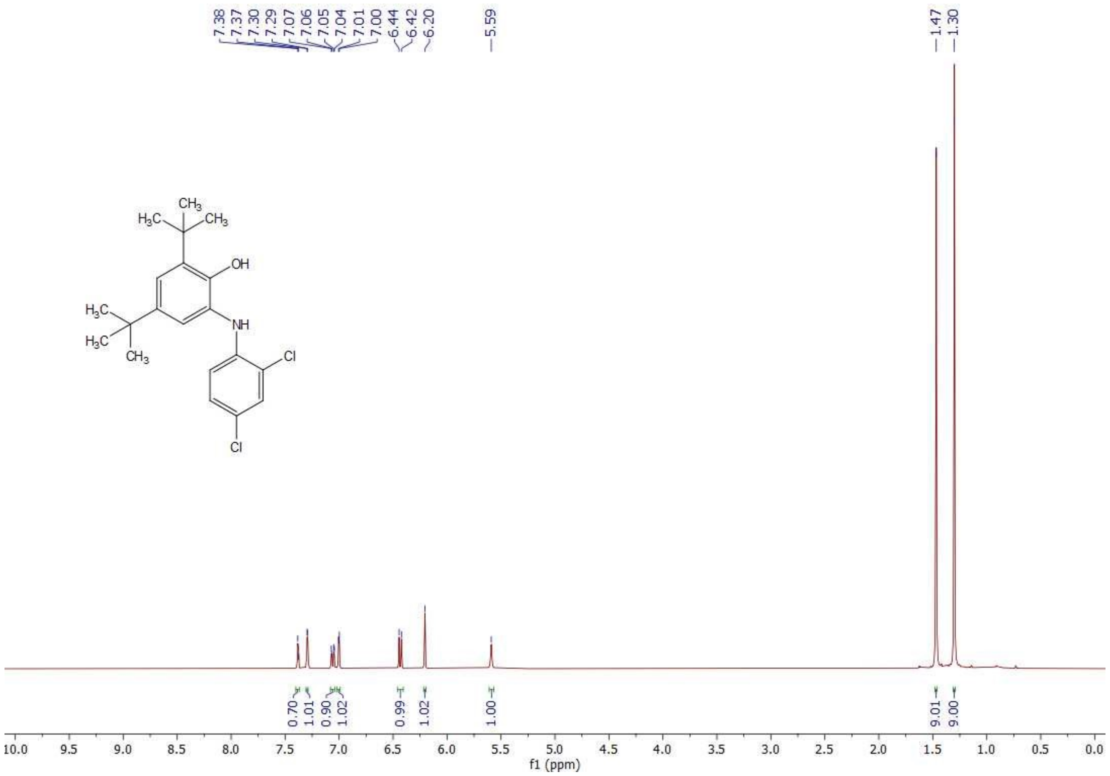

Figure S27. ^{1}H (400 MHz, $CDCl_3$), compound **3n**

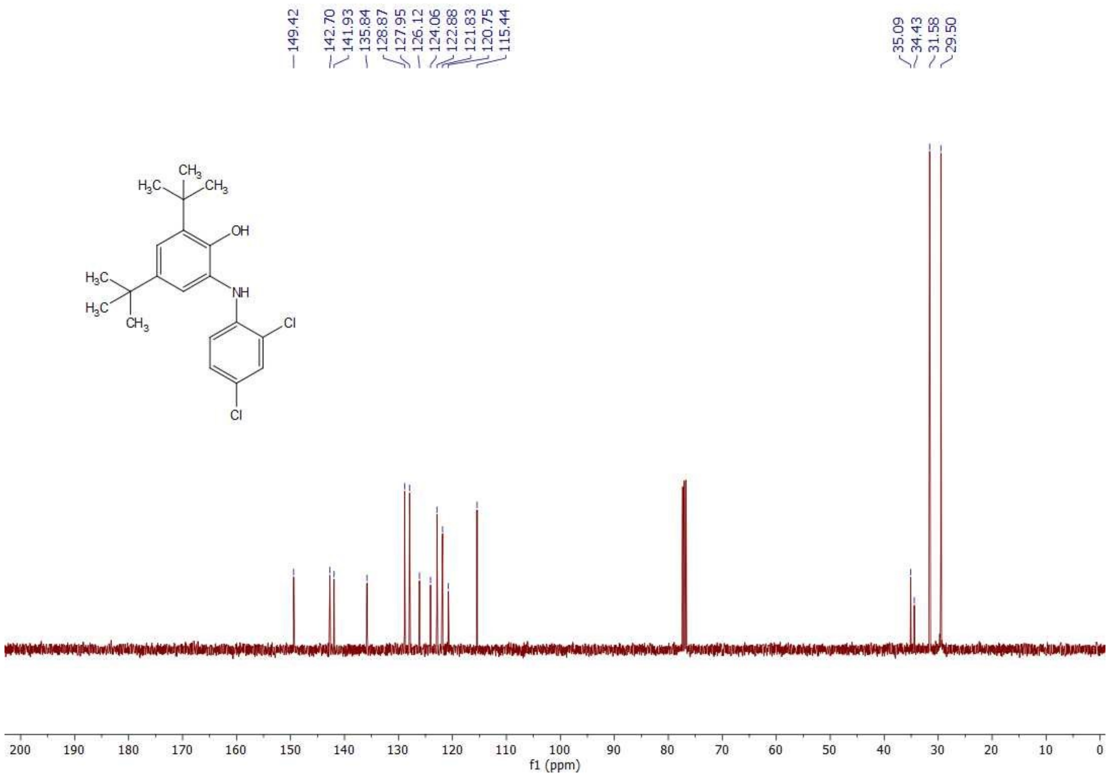

Figure S28. ^{13}C (100 MHz, $CDCl_3$), compound **3n**

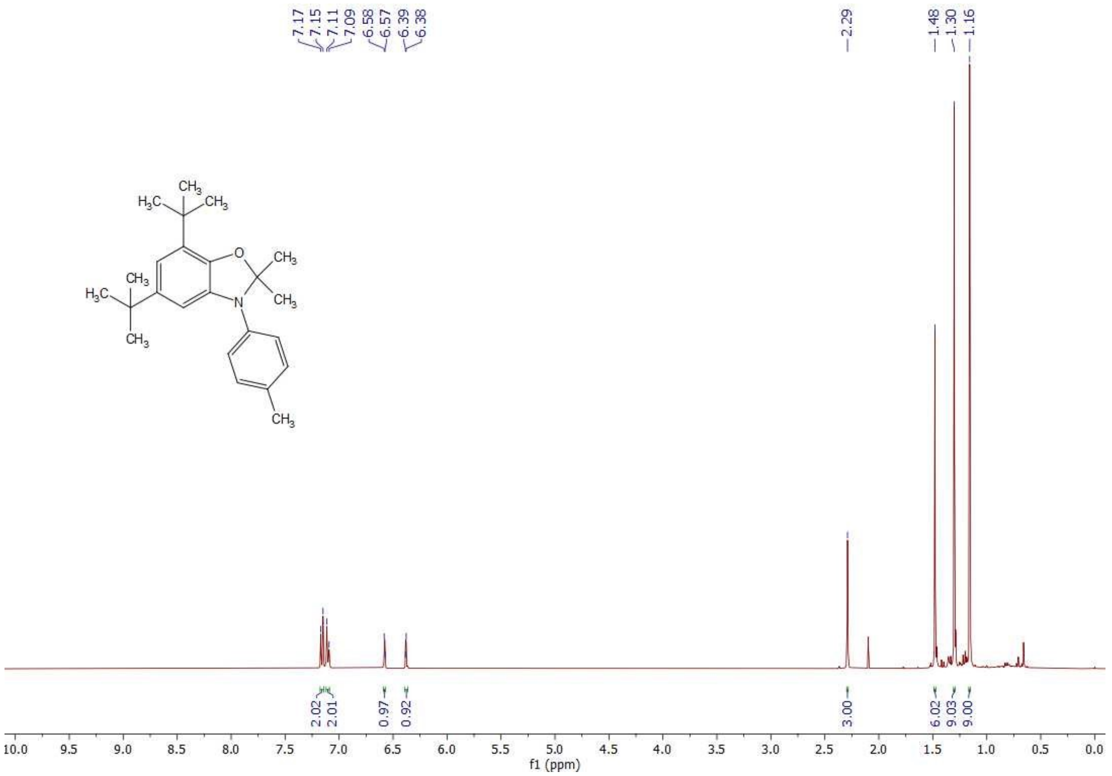

Figure S29. ^{1}H (400 MHz, $CDCl_3$), compound **5a**

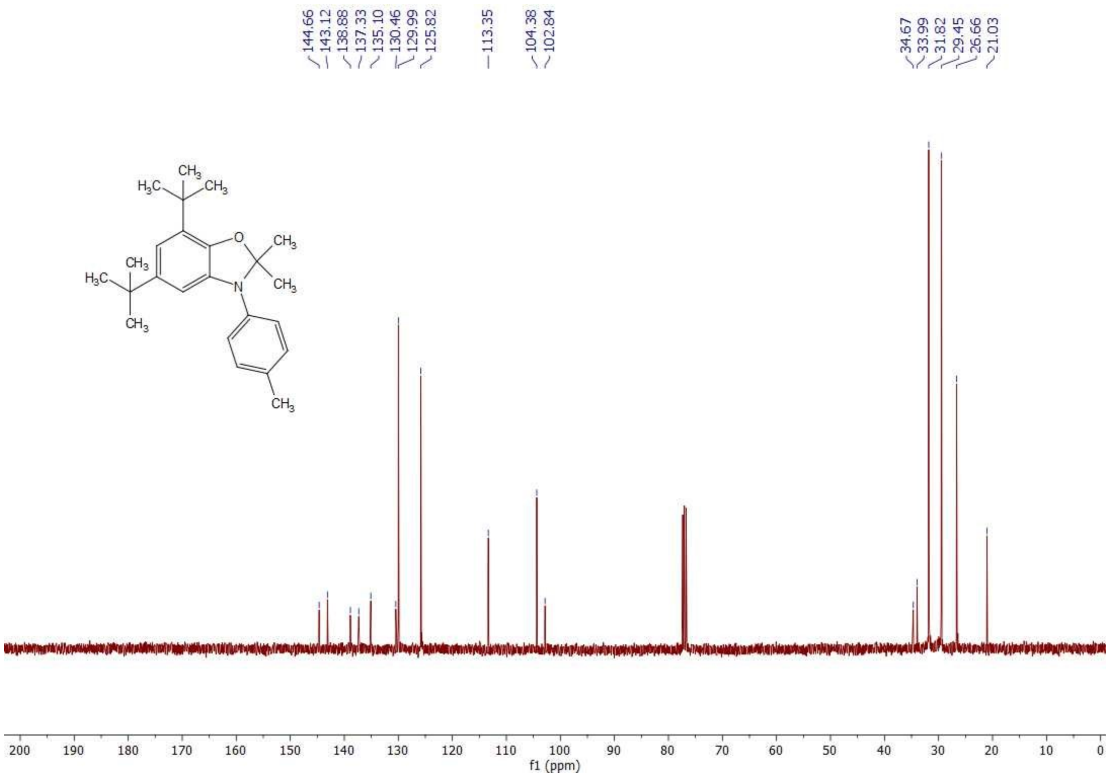

Figure S30. ^{13}C (100 MHz, $CDCl_3$), compound **5a**

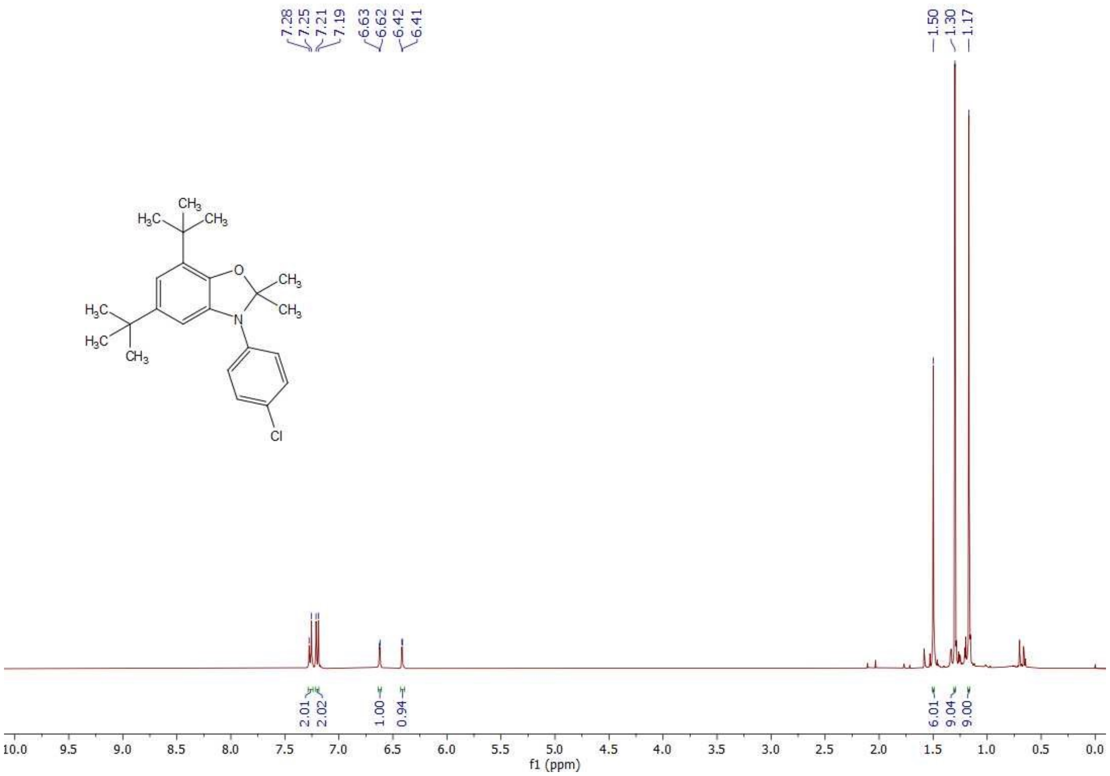

Figure S31. ^{1}H (400 MHz, $CDCl_3$), compound **5b**

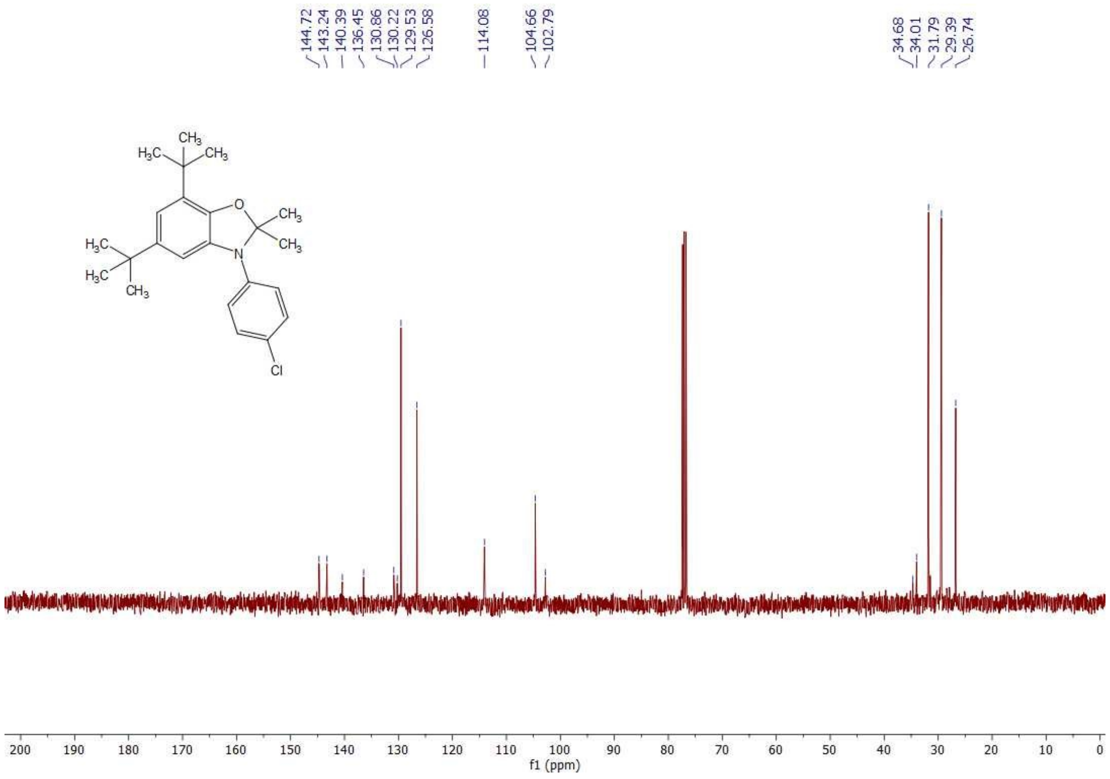

Figure S32. ^{13}C (100 MHz, $CDCl_3$), compound **5b**

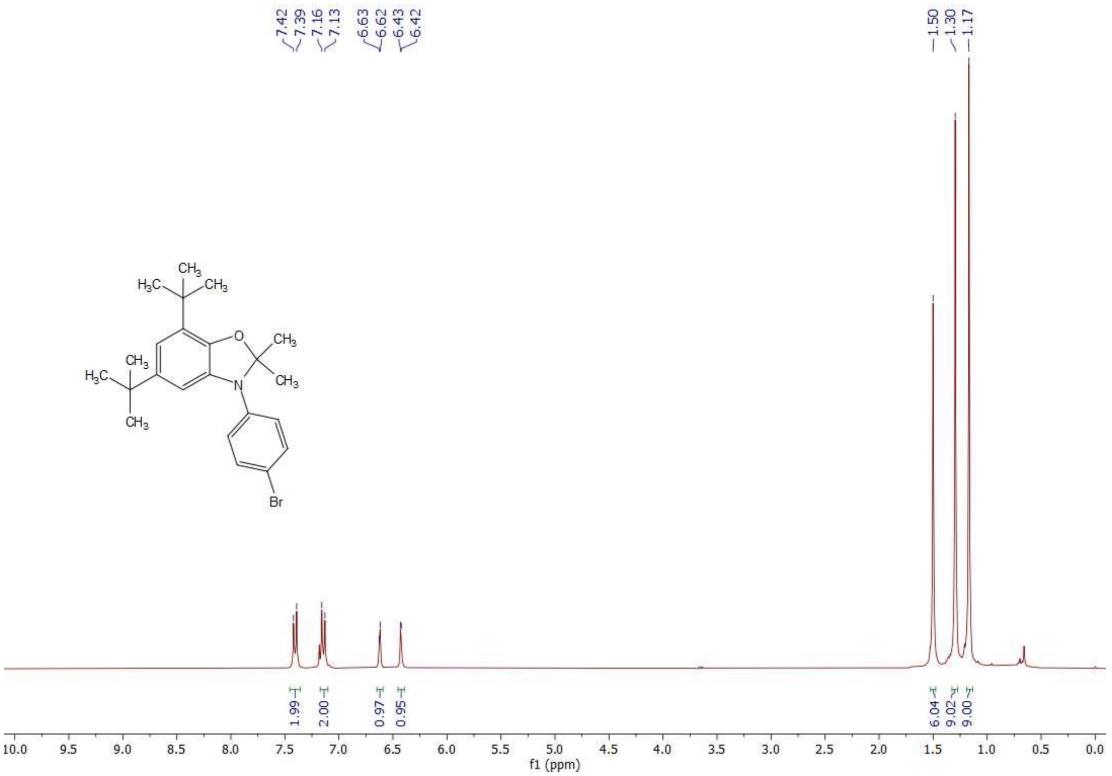

Figure S33. ^{1}H (300 MHz, $CDCl_3$), compound **5c**

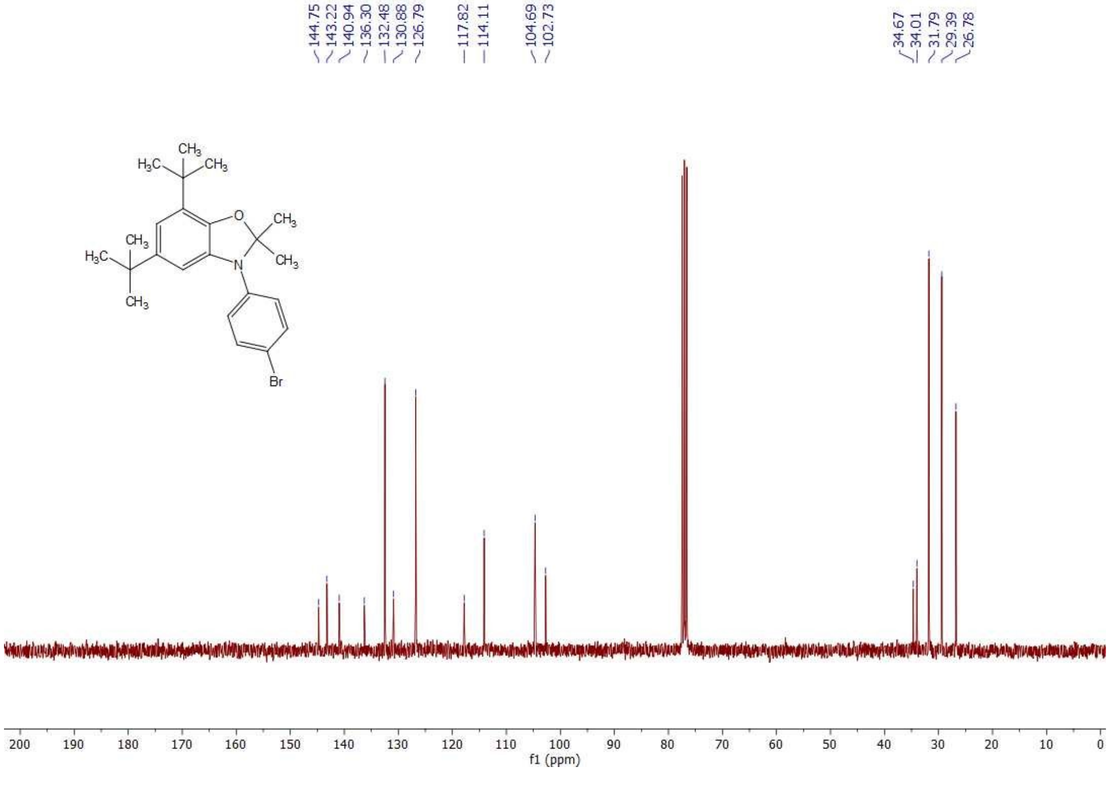

Figure S34. ^{13}C (75 MHz, $CDCl_3$), compound **5c**

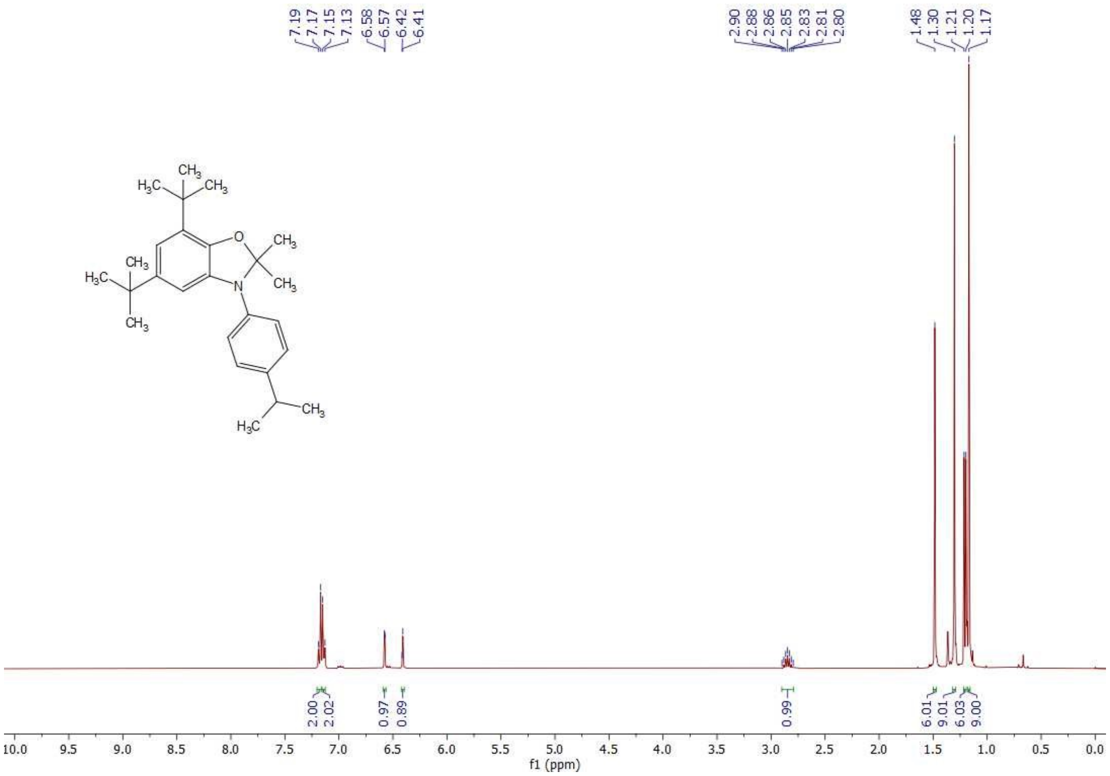

Figure S35. ^{1}H (400 MHz, $CDCl_3$), compound **5d**

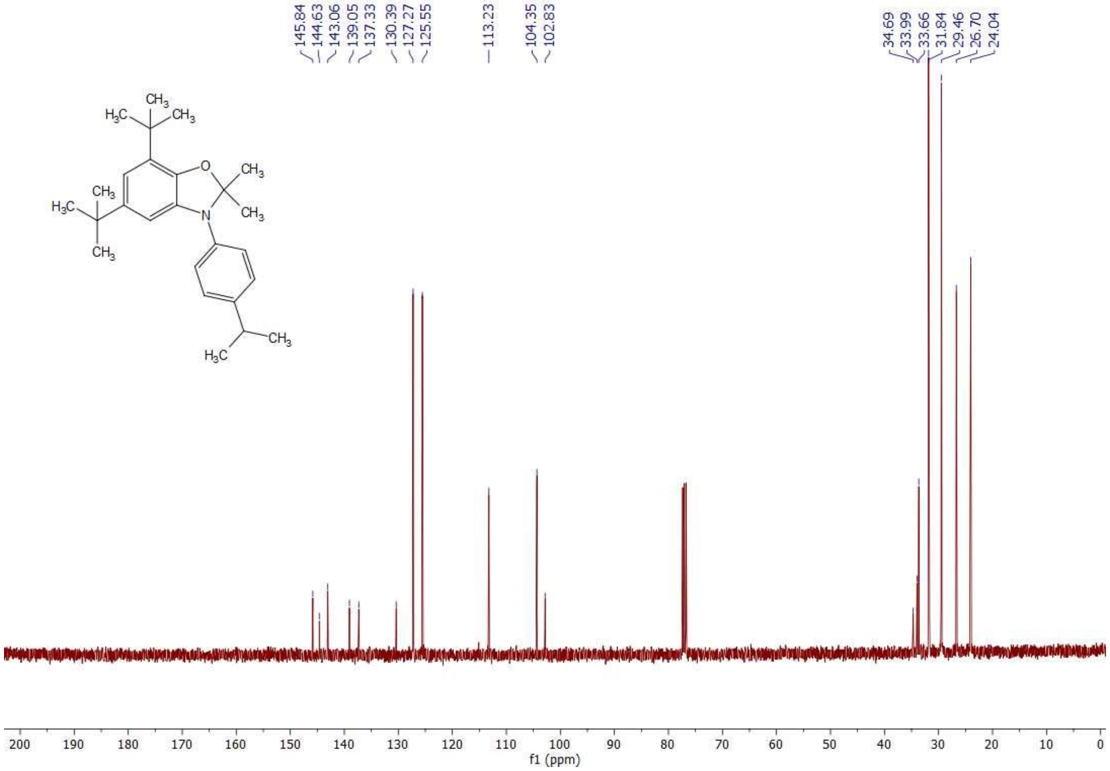

Figure S36. ^{13}C (100 MHz, $CDCl_3$), compound **5d**

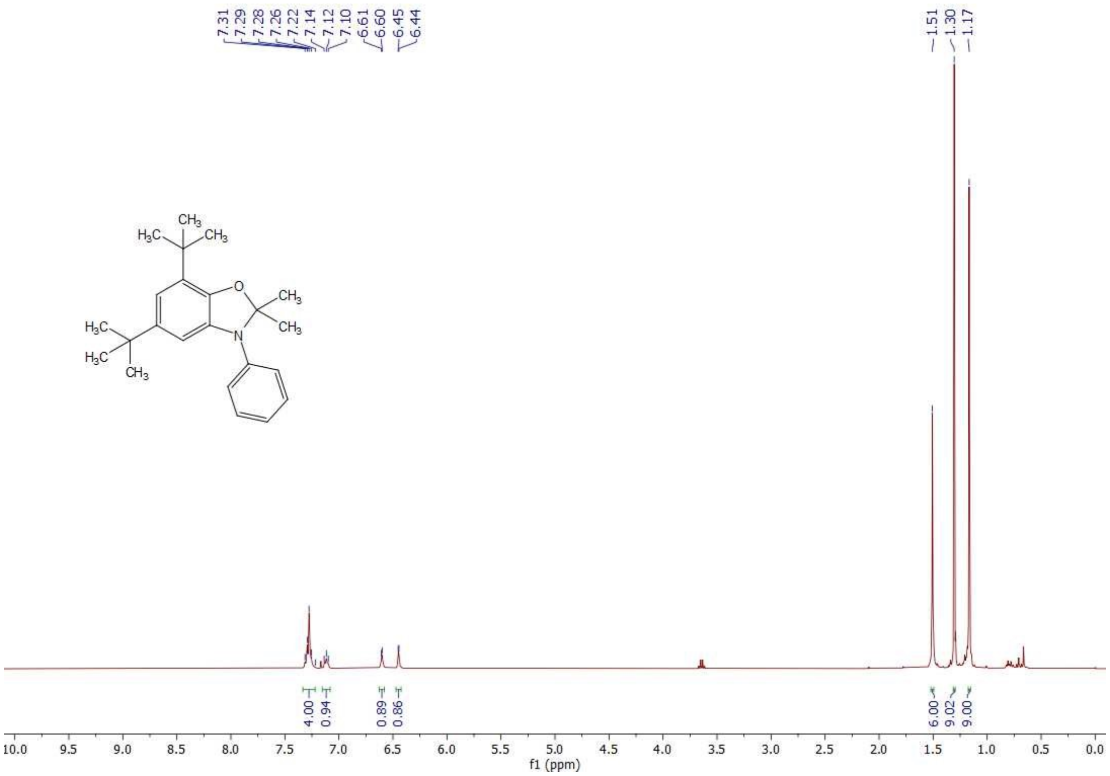

Figure S37. ^{1}H (400 MHz, $CDCl_3$), compound **5e**

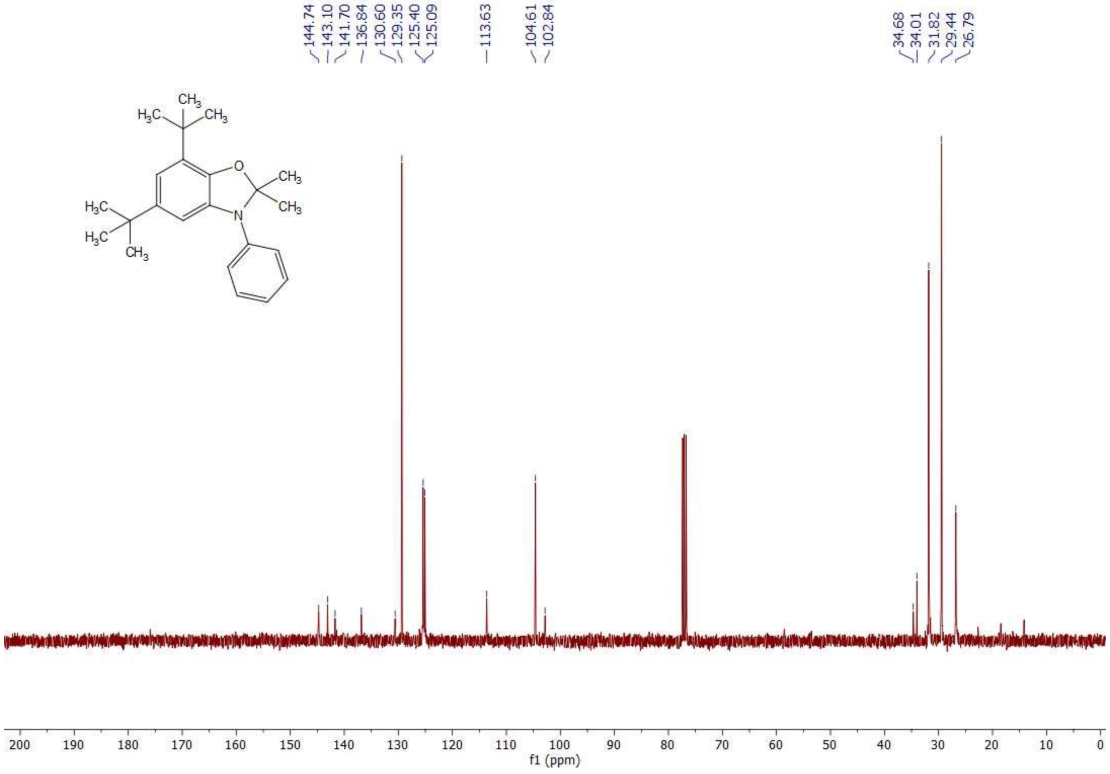

Figure S38. ^{13}C (100 MHz, $CDCl_3$), compound **5e**

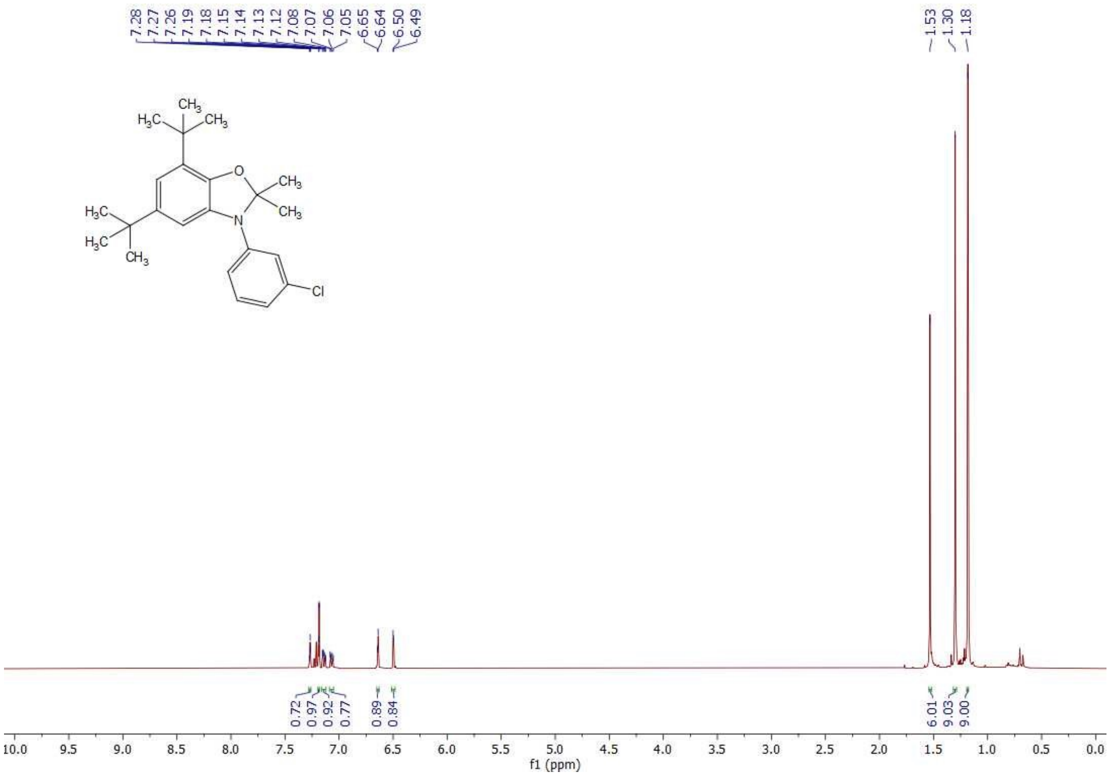

Figure S39. ^{1}H (400 MHz, $CDCl_3$), compound **5f**

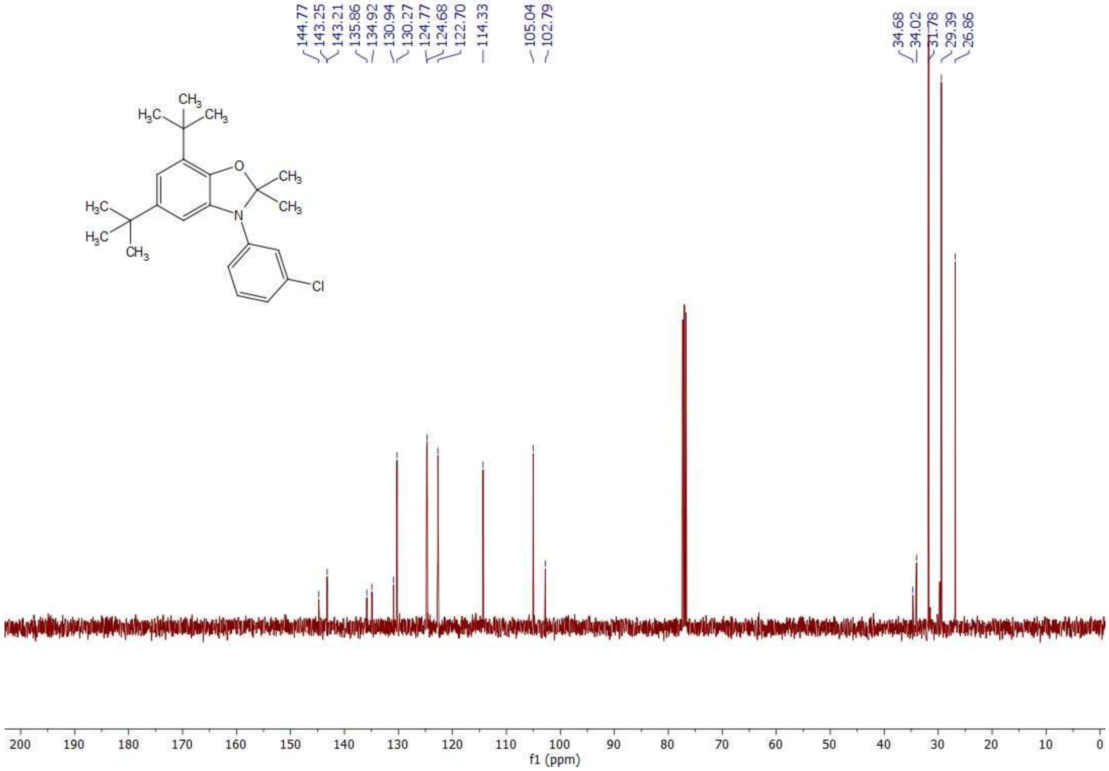

Figure S40. ^{13}C (100 MHz, $CDCl_3$), compound **5f**

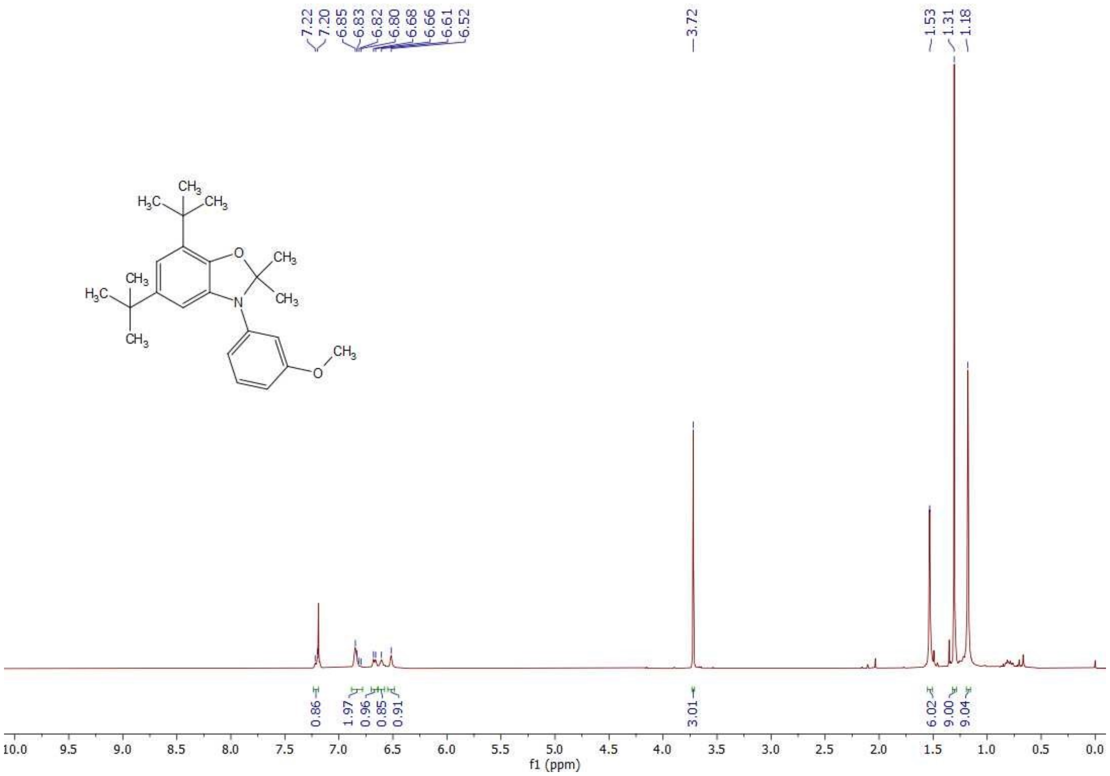

Figure S41. ^{1}H (400 MHz, $CDCl_3$), compound **5g**

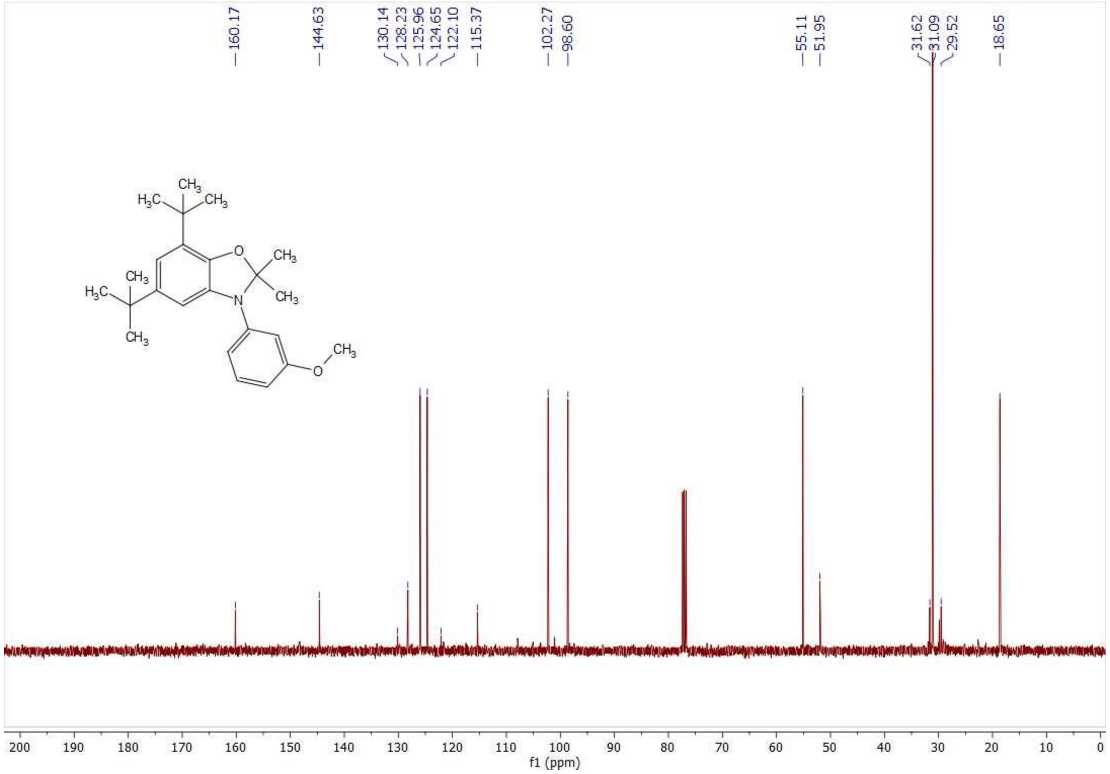

Figure S42. ^{13}C (100 MHz, $CDCl_3$), compound **5g**

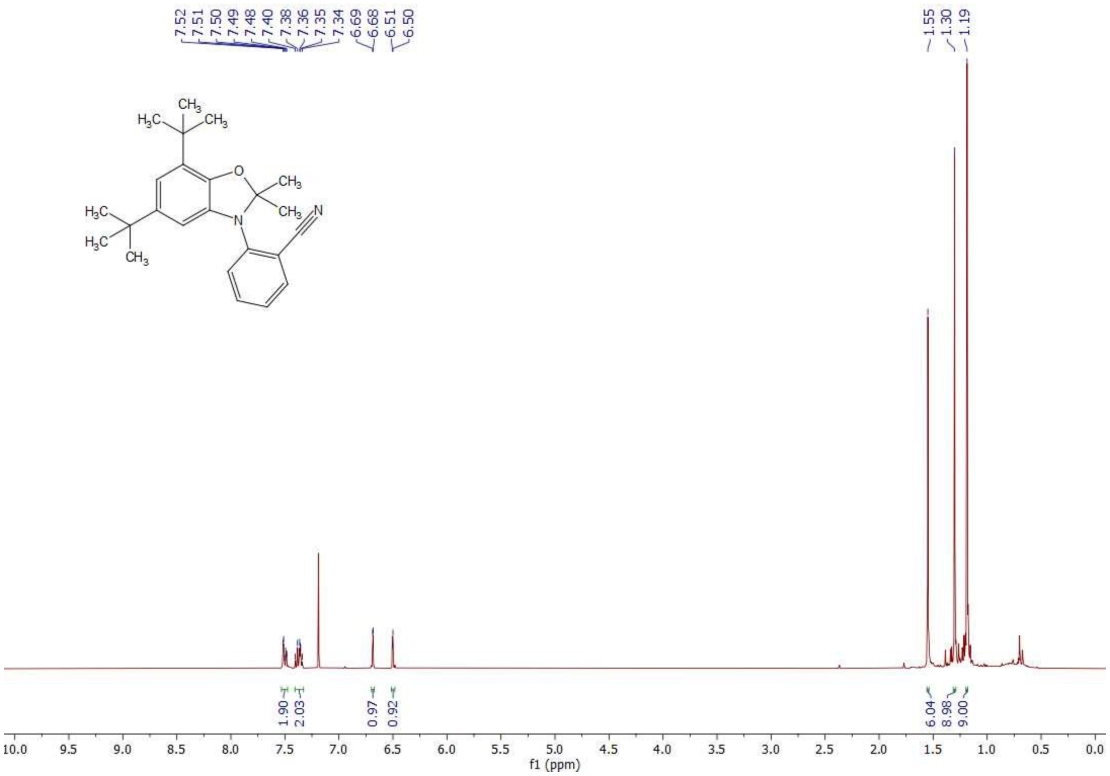

Figure S43. ^{1}H (400 MHz, $CDCl_3$), compound **5h**

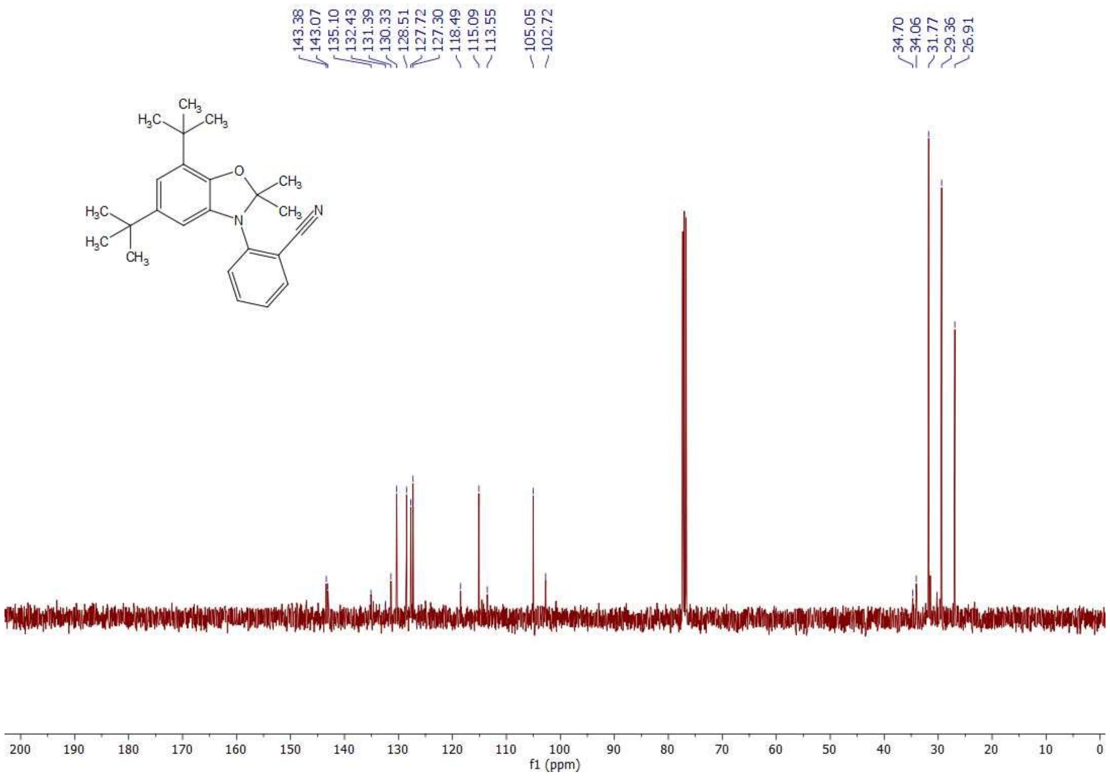

Figure S44. ^{13}C (100 MHz, $CDCl_3$), compound **5h**

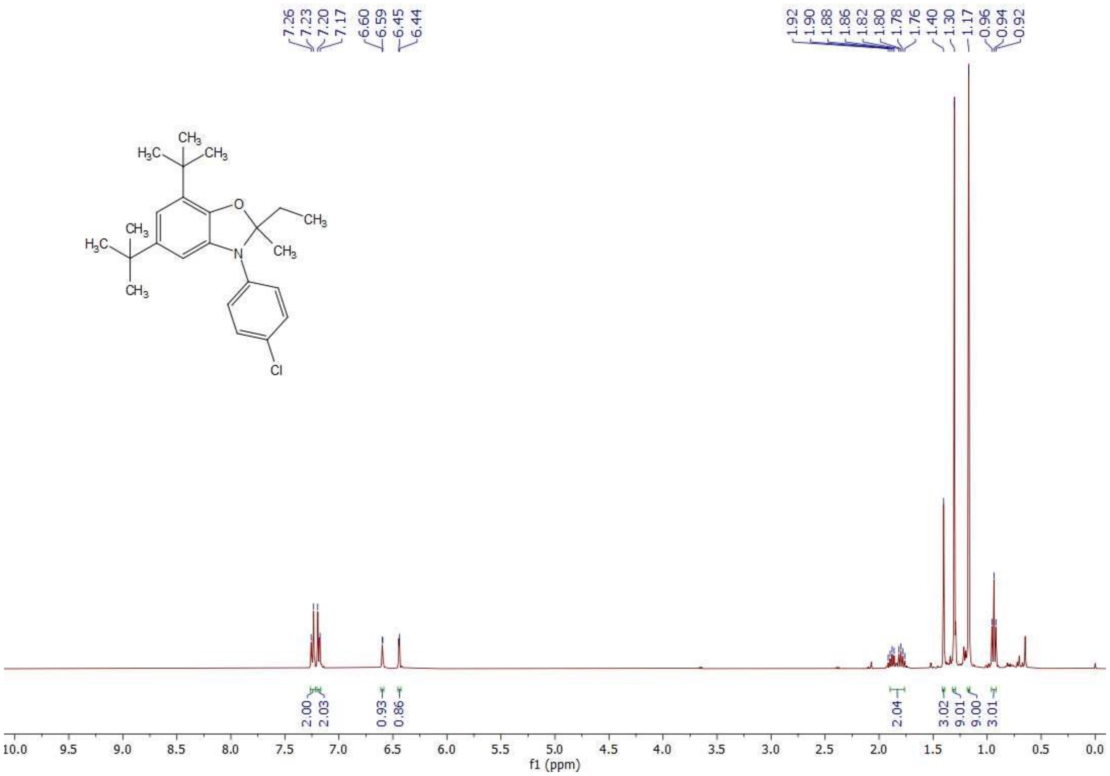

Figure S45. ^{1}H (400 MHz, $CDCl_3$), compound **5i**

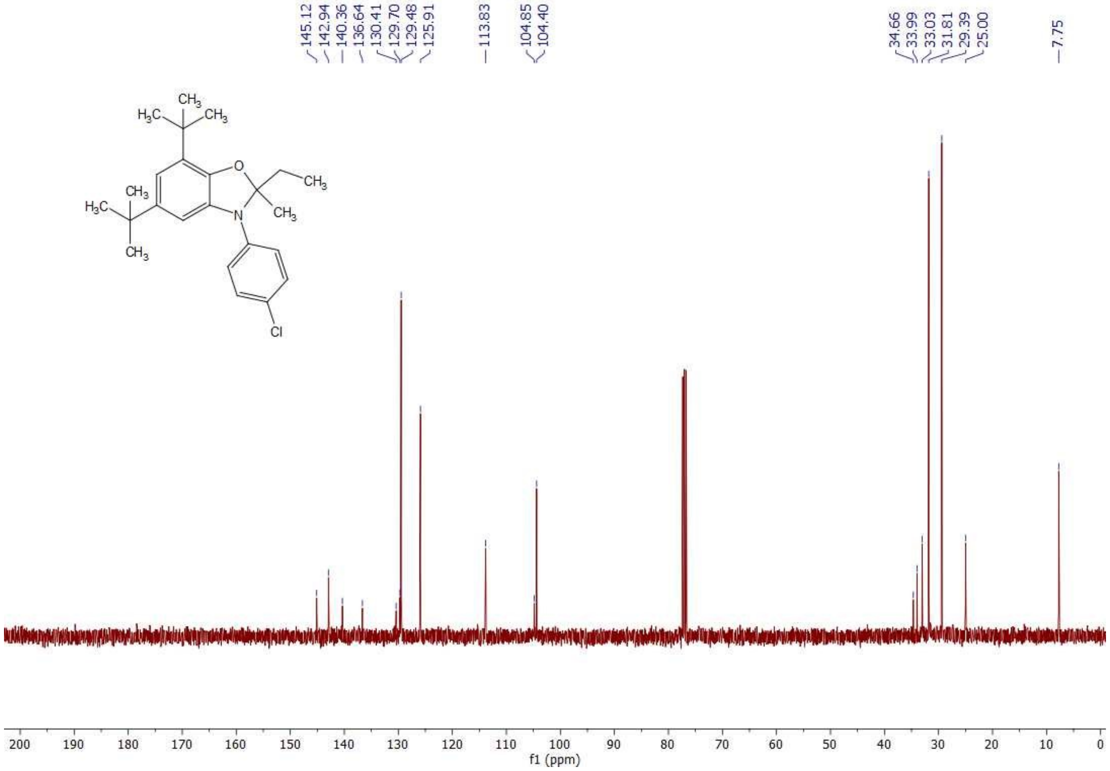

Figure S46. ^{13}C (100 MHz, $CDCl_3$), compound **5i**

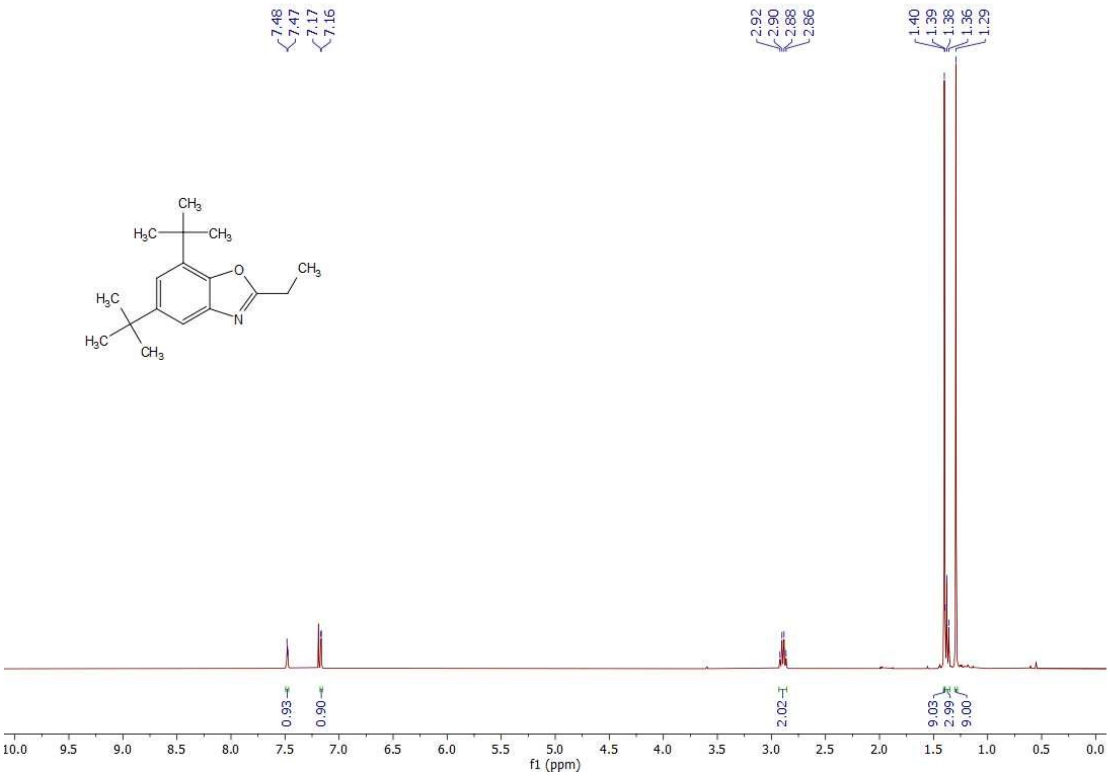

Figure S47. ^{1}H (400 MHz, $CDCl_3$), compound **7a**

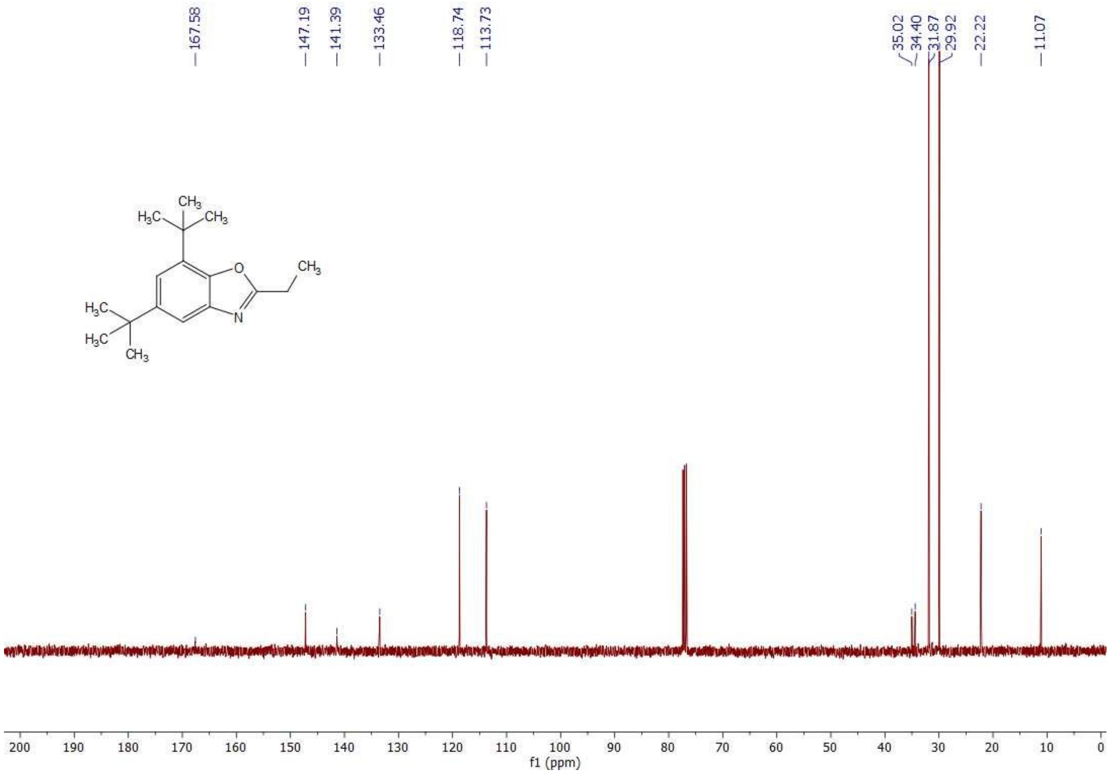

Figure S48. ^{13}C (100 MHz, $CDCl_3$), compound **7a**

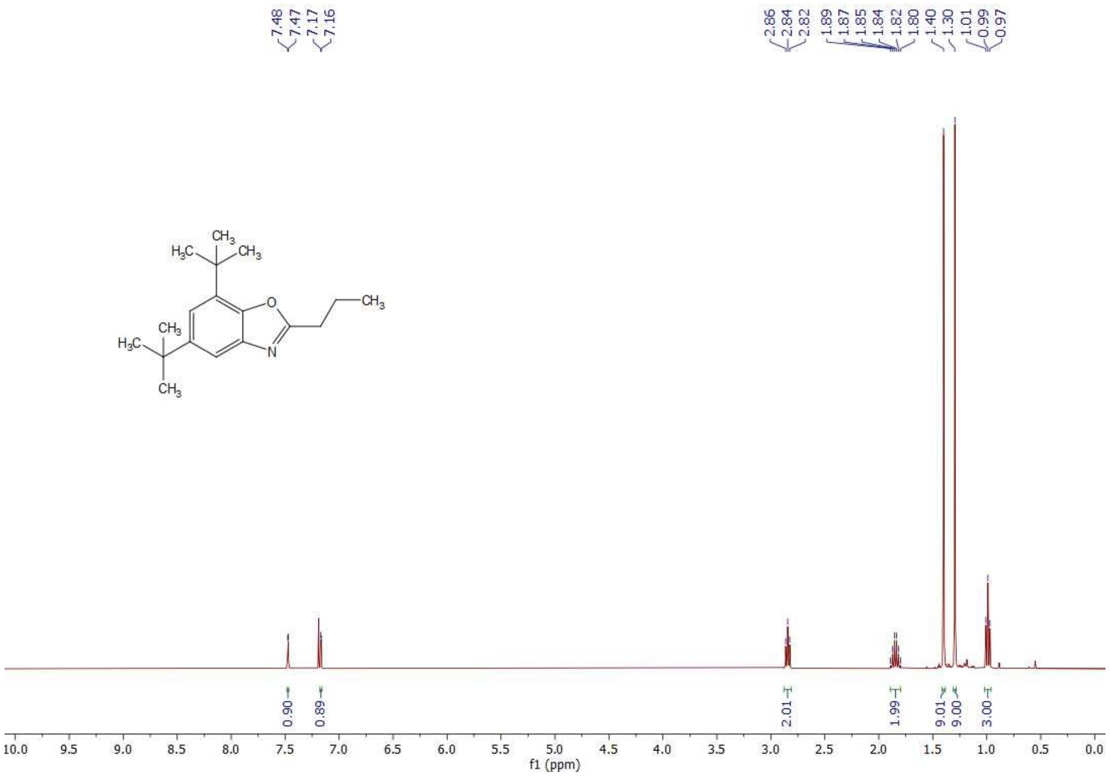

Figure S49. ^{1}H (400 MHz, $CDCl_3$), compound **7b**

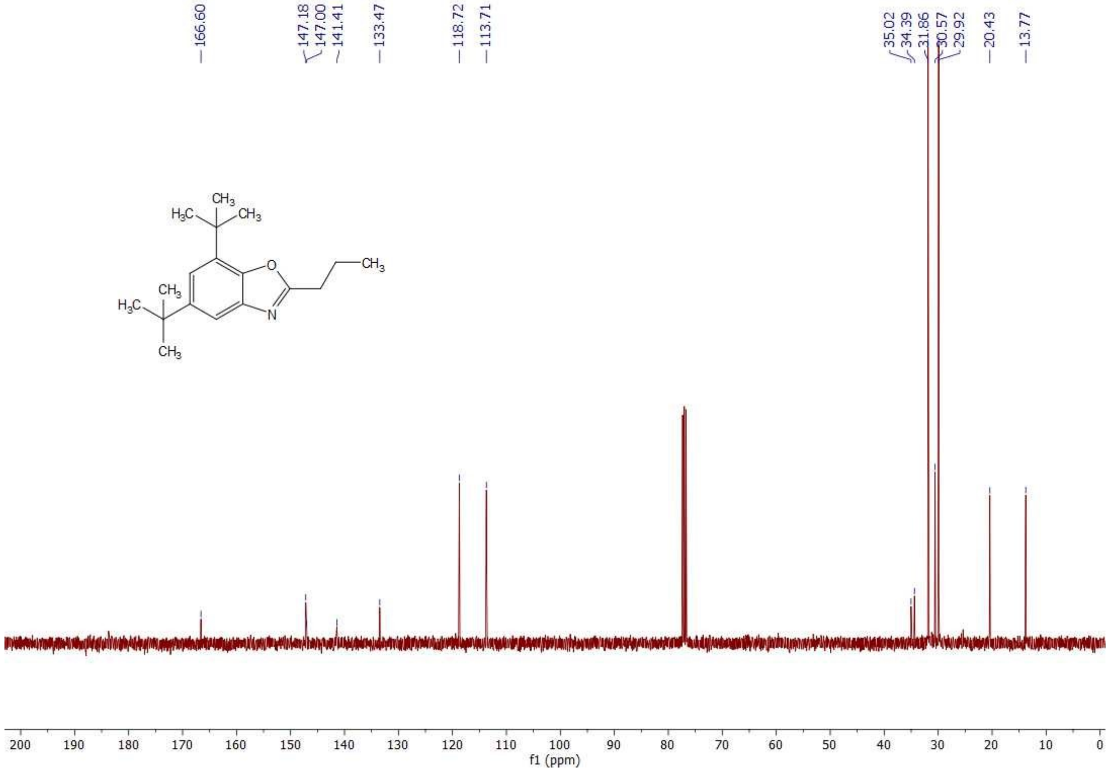

Figure S50. ^{13}C (100 MHz, $CDCl_3$), compound **7b**

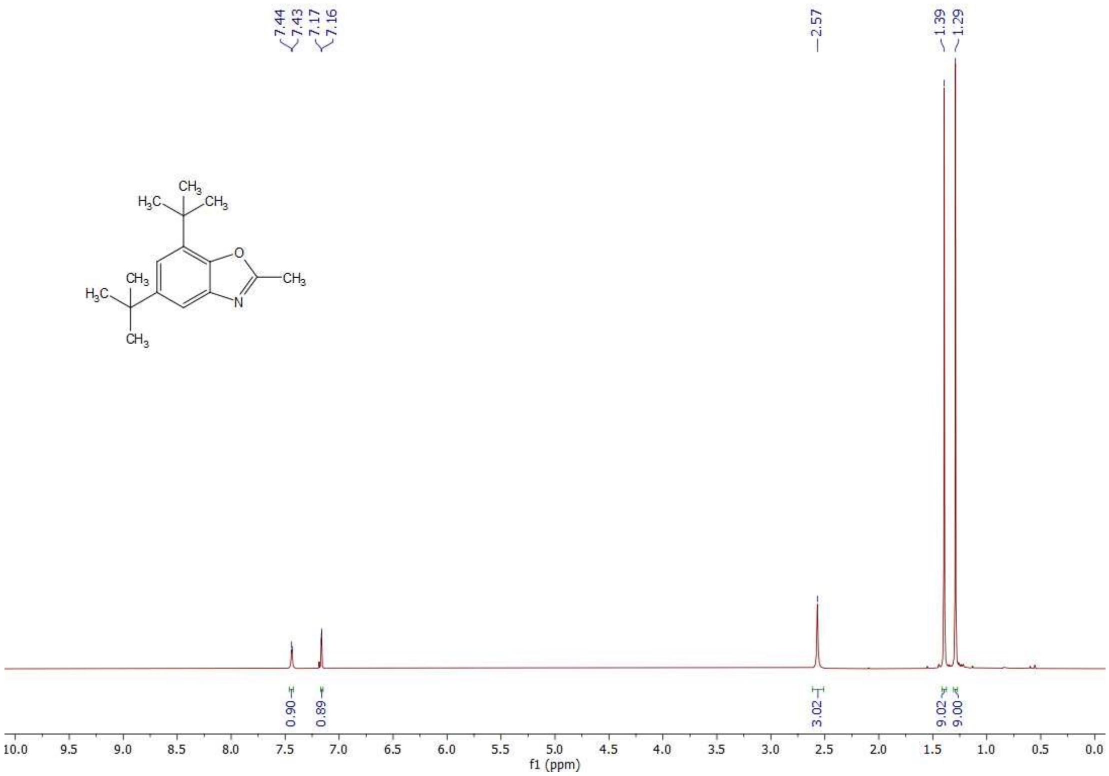

Figure S51. ^{1}H (400 MHz, $CDCl_3$), compound **7c**

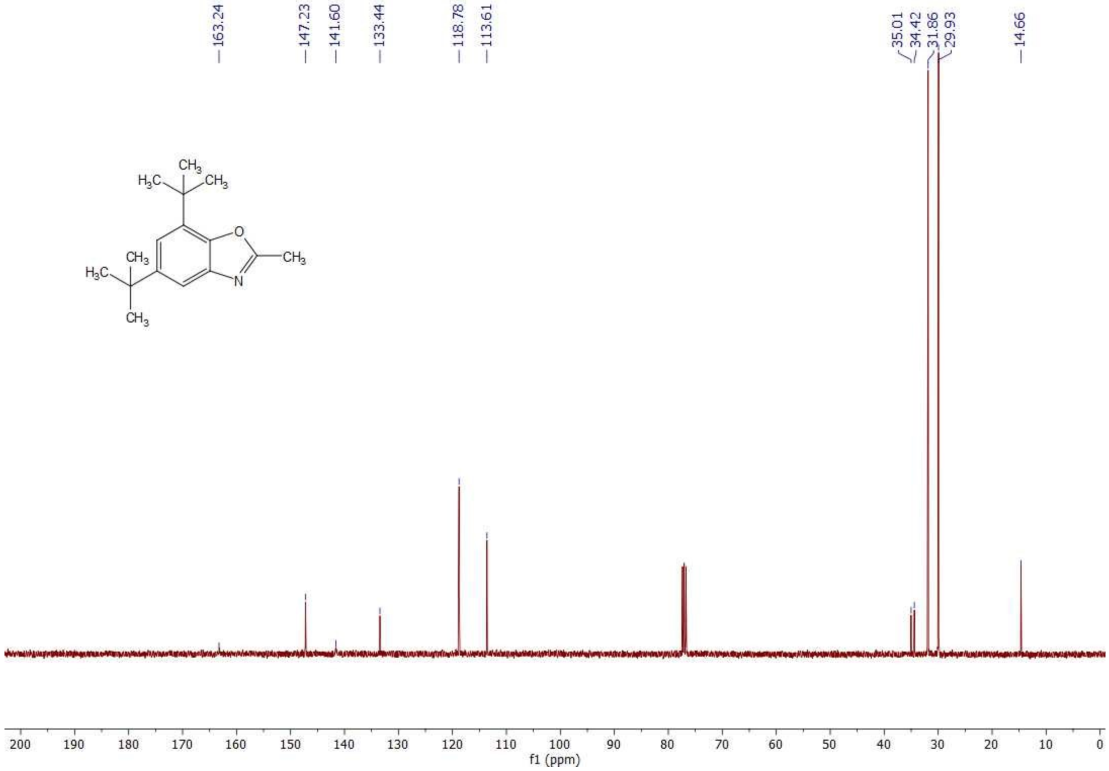

Figure S52. ^{13}C (100 MHz, $CDCl_3$), compound **7c**

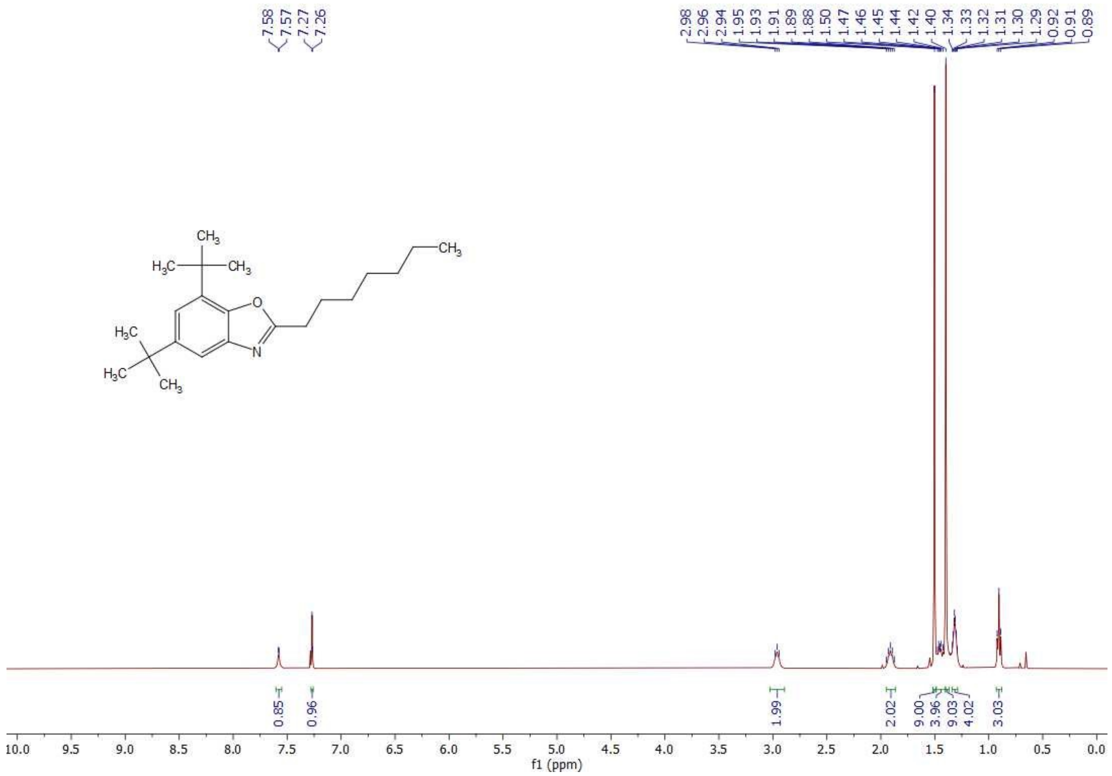

Figure S53. ^{1}H (400 MHz, $CDCl_3$), compound **7d**

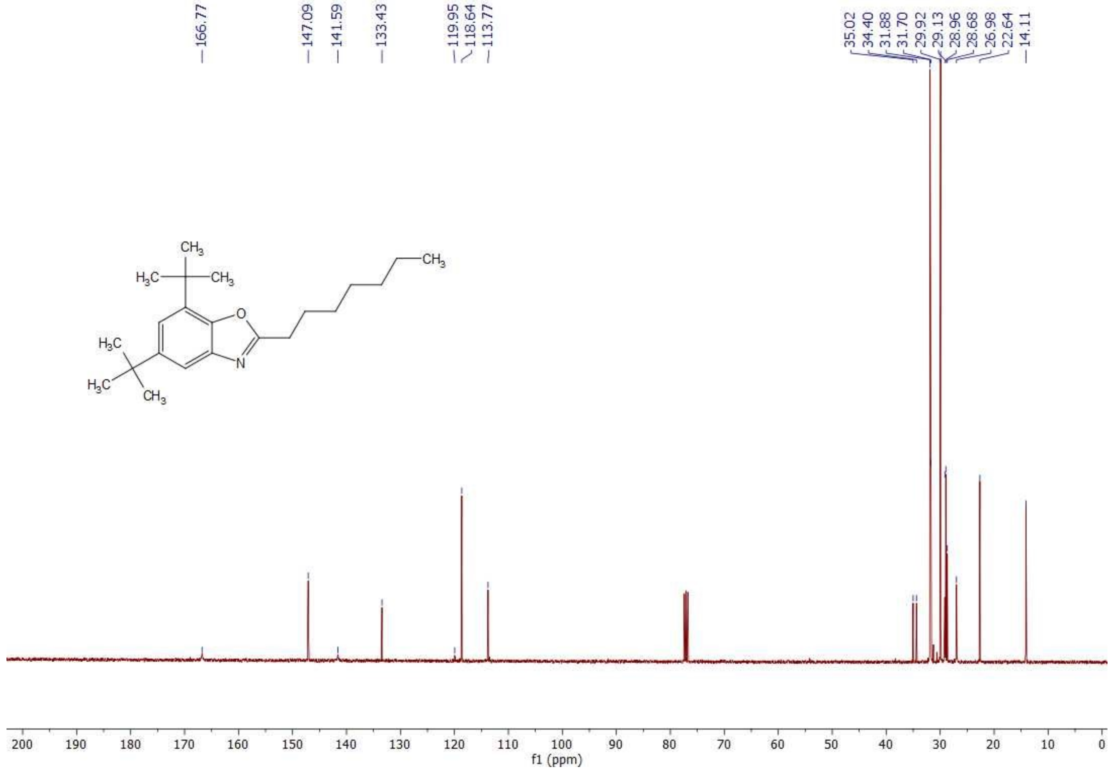

Figure S54. ^{13}C (100 MHz, $CDCl_3$), compound **7d**

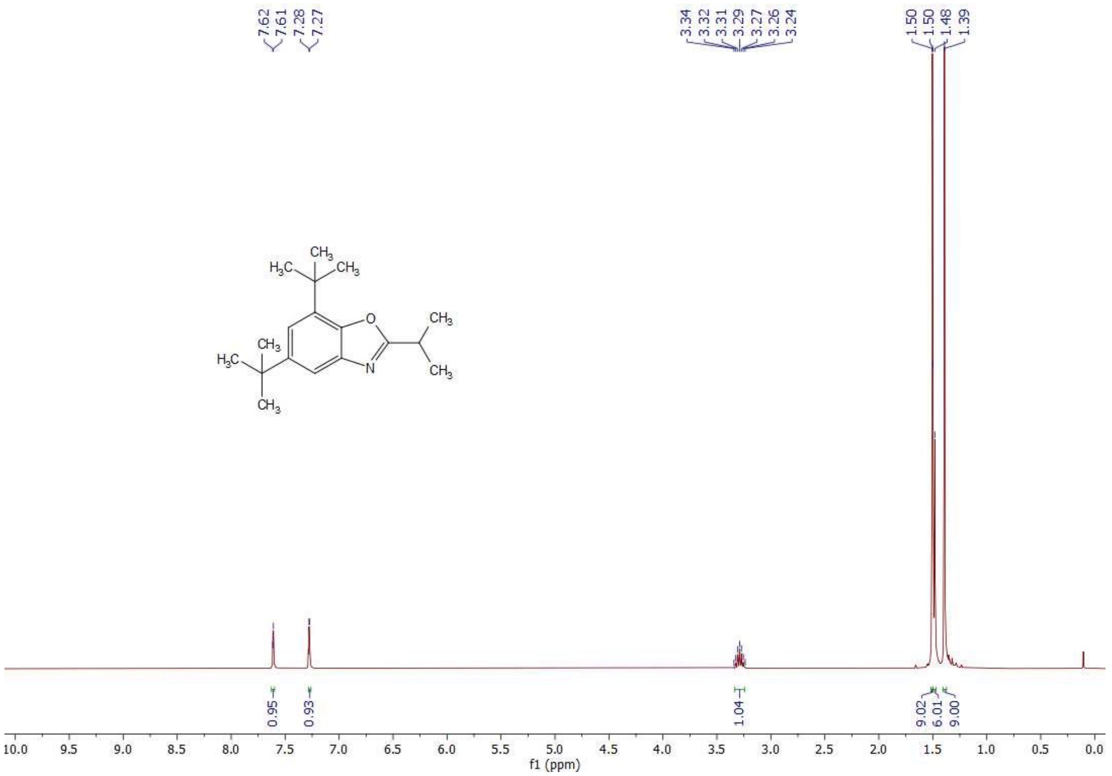

Figure S55. ^{1}H (400 MHz, $CDCl_3$), compound **7e**

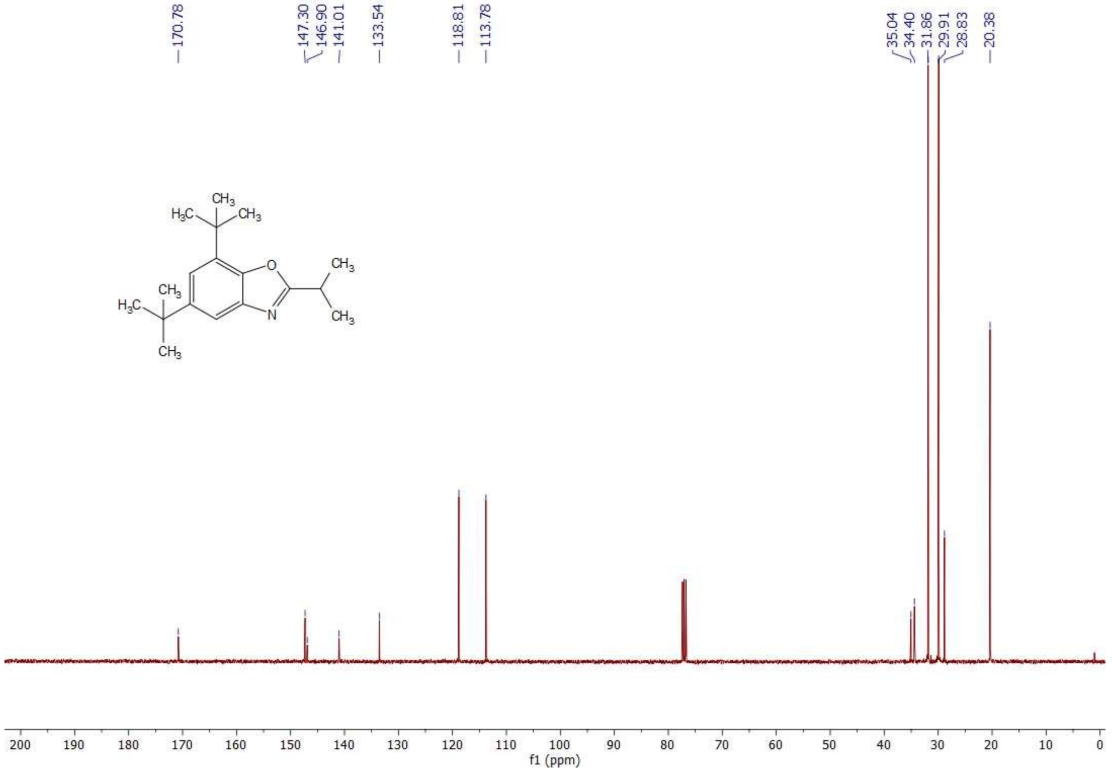

Figure S56. ^{13}C (100 MHz, $CDCl_3$), compound **7e**

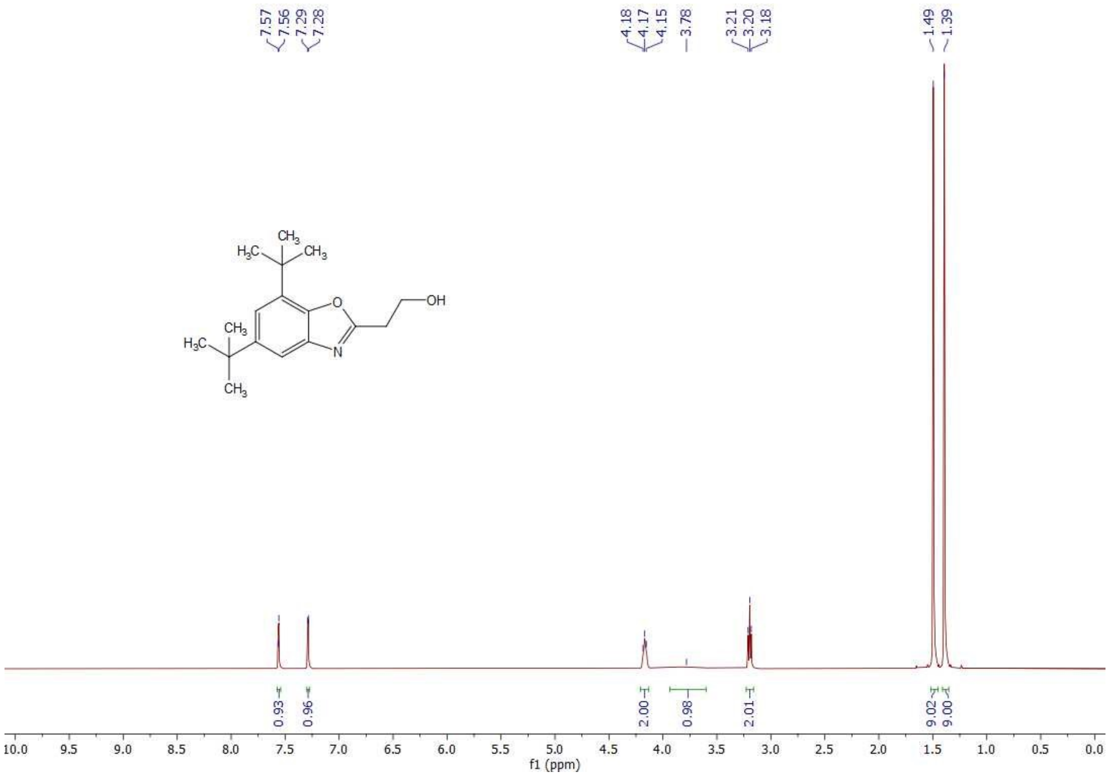

Figure S57. ^{1}H (400 MHz, $CDCl_3$), compound **7f**

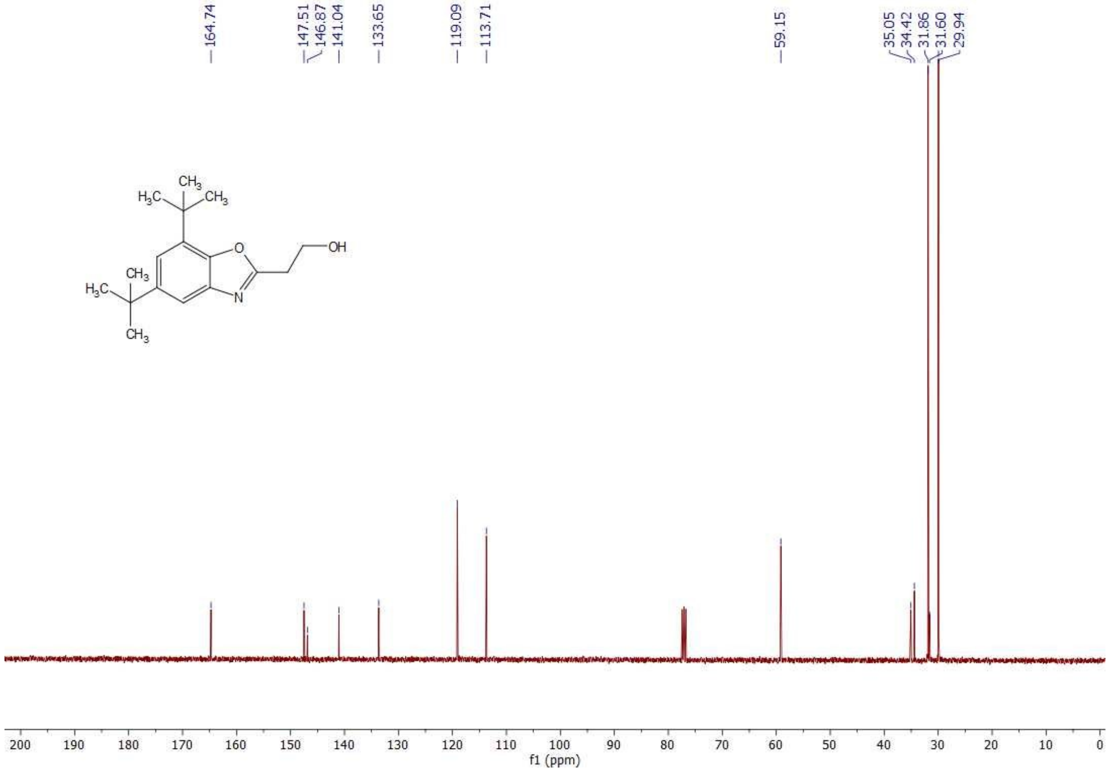

Figure S58. ^{13}C (100 MHz, $CDCl_3$), compound **7f**

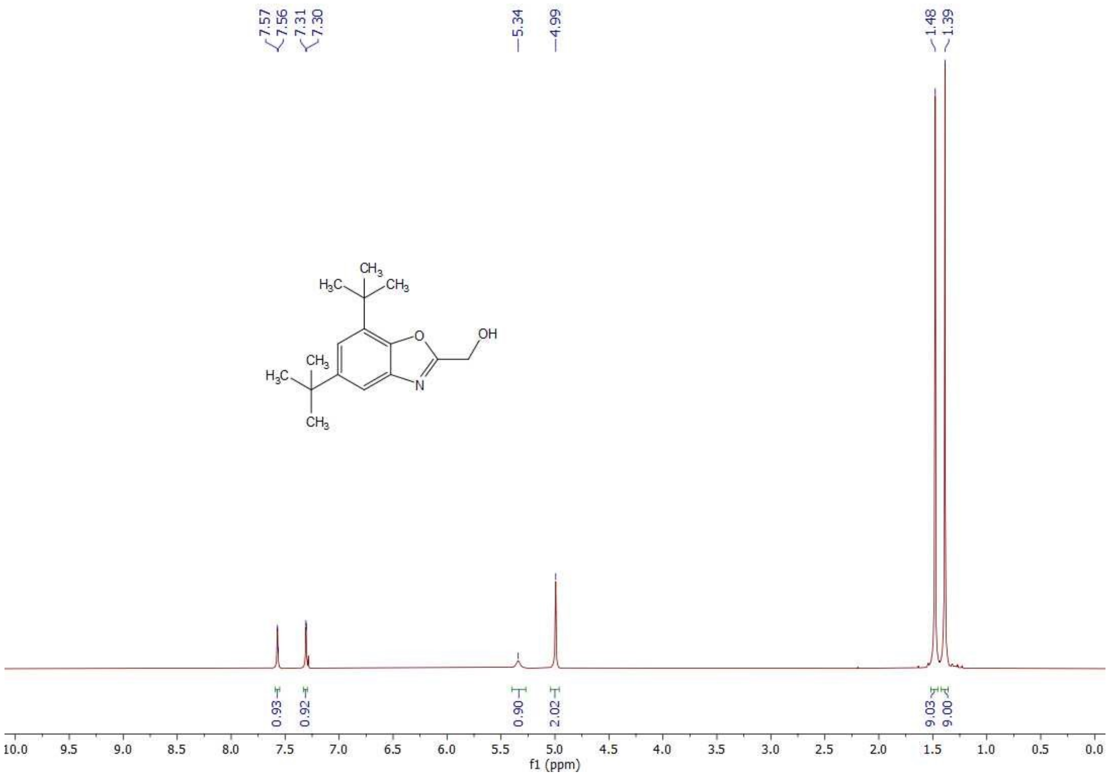

Figure S59. ^{1}H (400 MHz, $CDCl_3$), compound **7g**

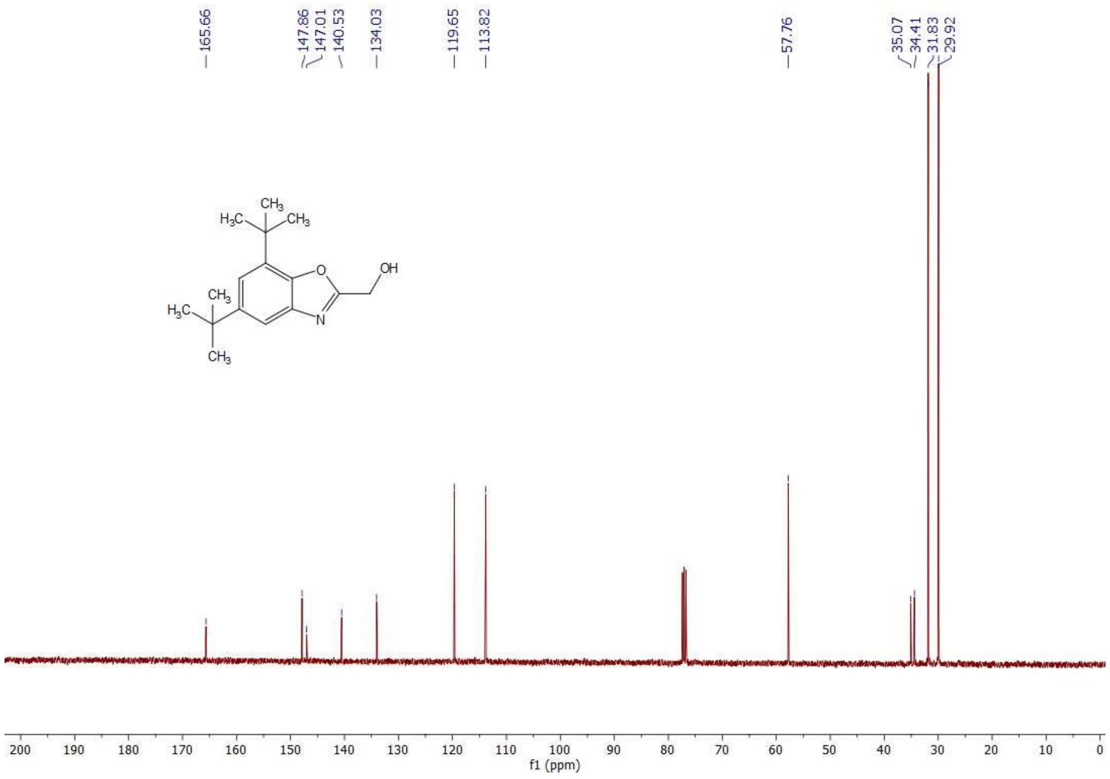

Figure S60. ^{13}C (100 MHz, $CDCl_3$), compound **7g**

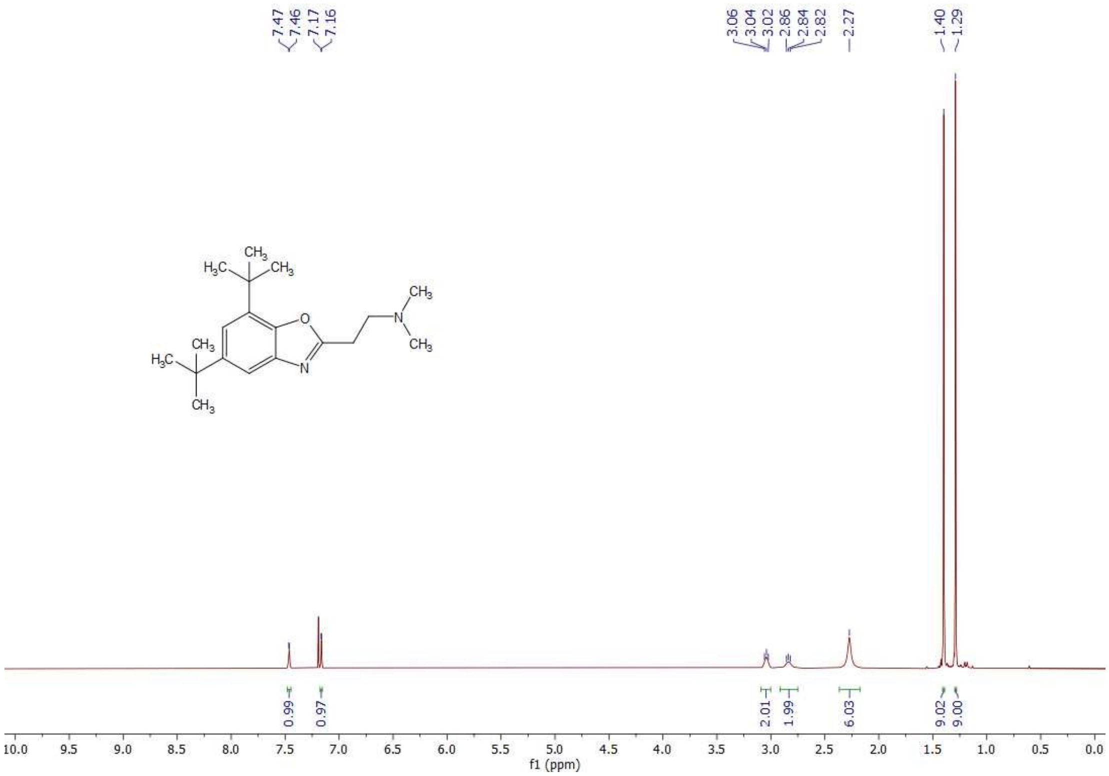

Figure S61. ^{1}H (400 MHz, $CDCl_3$), compound **7h**

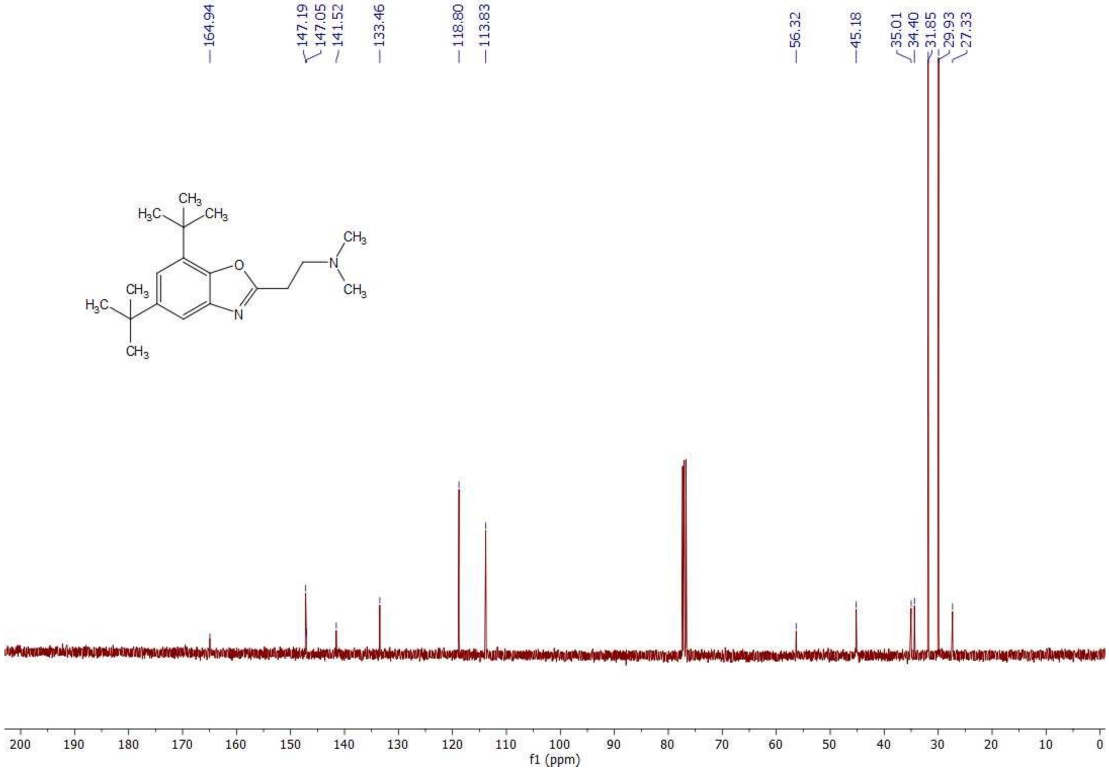

Figure S62. ^{13}C (100 MHz, $CDCl_3$), compound **7h**

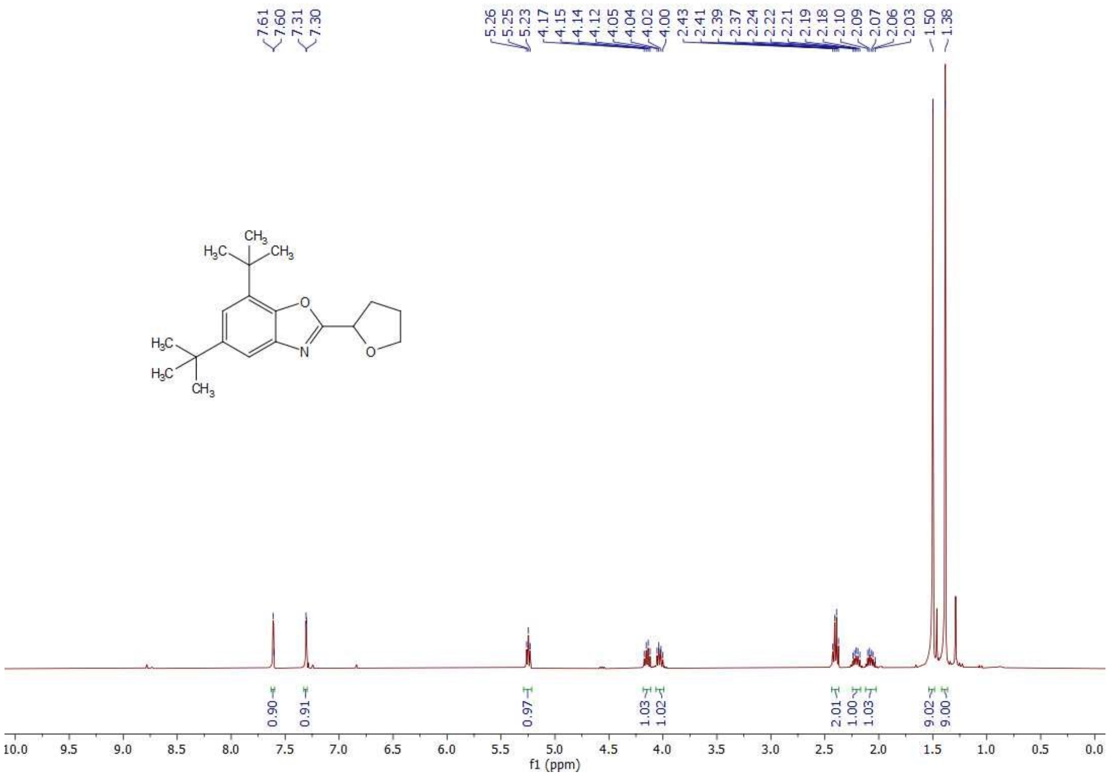

Figure S63. ^{1}H (400 MHz, $CDCl_3$), compound **7i**

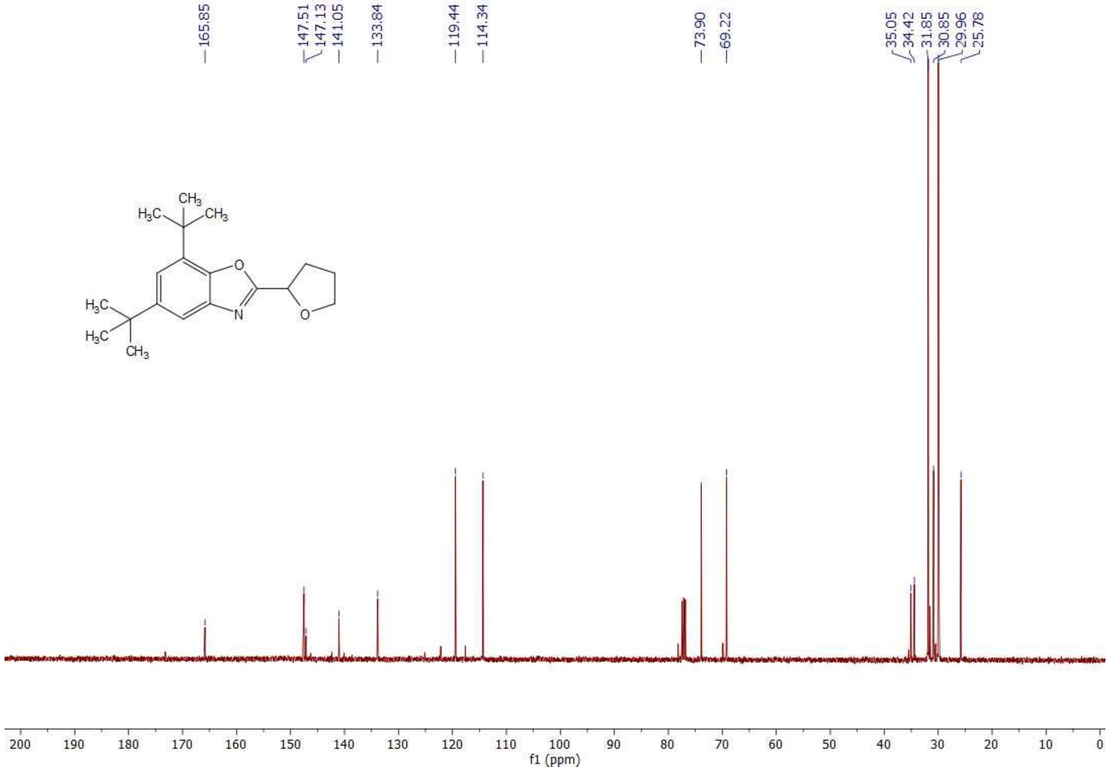

Figure S64. ^{13}C (100 MHz, $CDCl_3$), compound **7i**

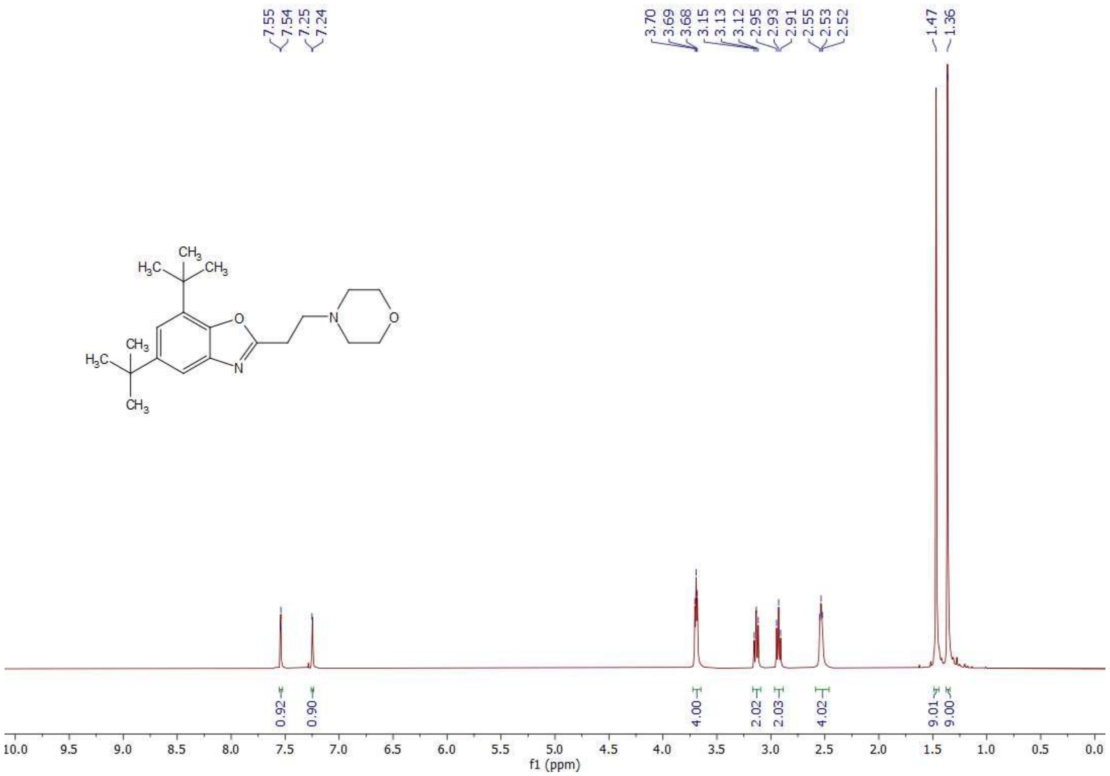

Figure S65. ^{1}H (400 MHz, $CDCl_3$), compound **7j**

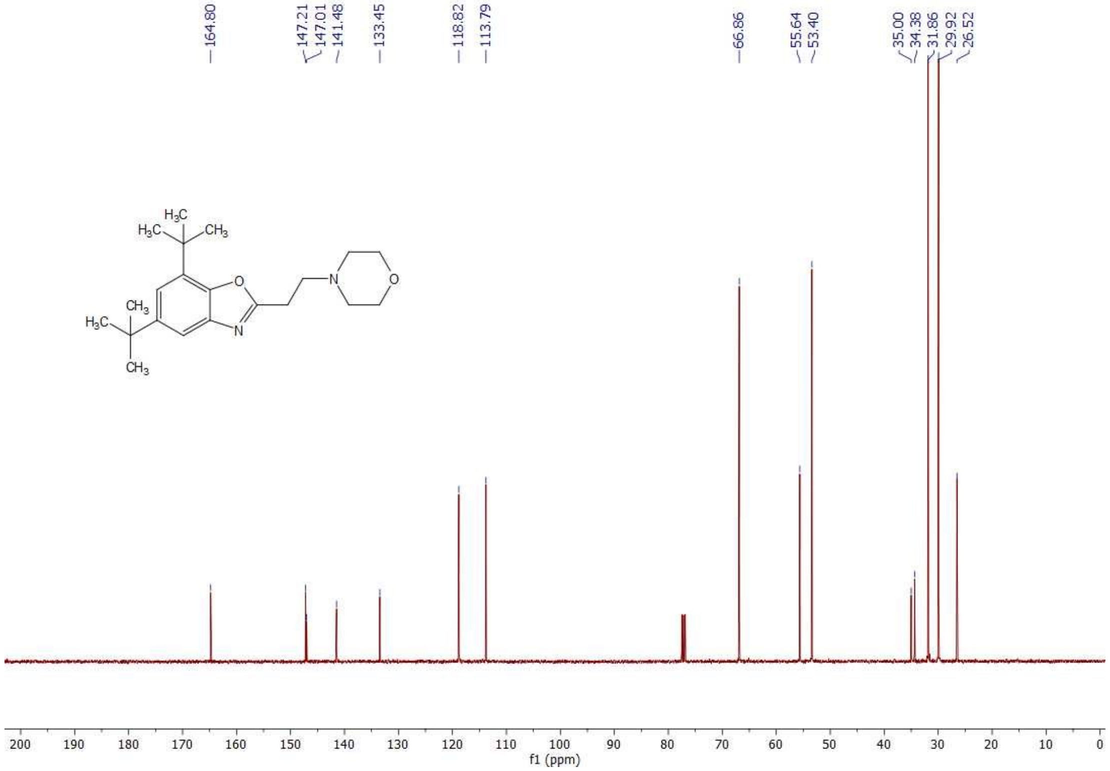

Figure S66. ^{13}C (100 MHz, $CDCl_3$), compound **7j**

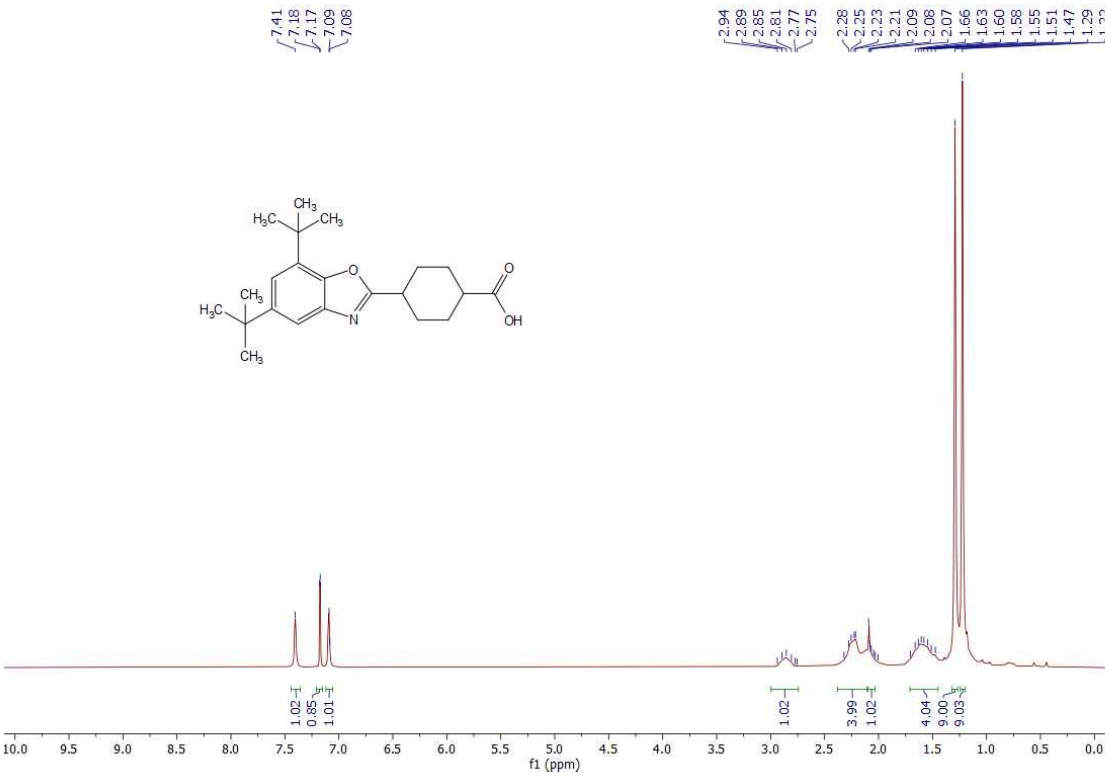

Figure S67. ^{1}H (400 MHz, $CDCl_3$), compound **7k**

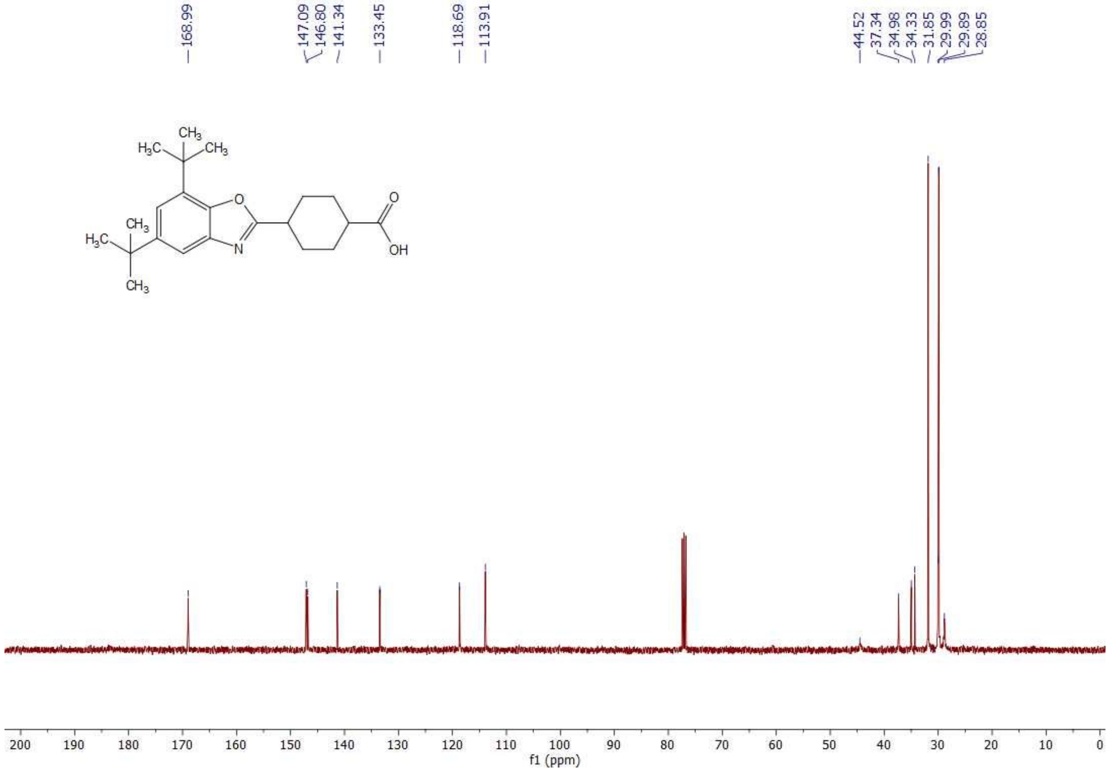

Figure S68. ^{13}C (100 MHz, $CDCl_3$), compound **7k**

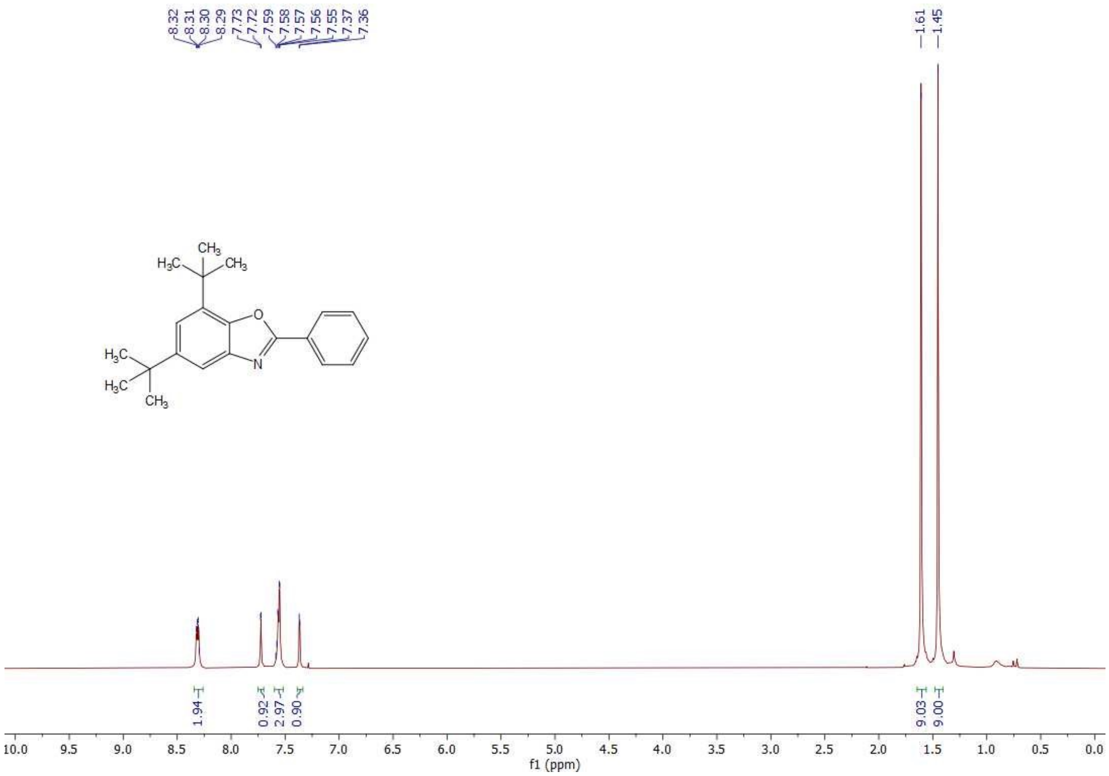

Figure S69. ^{1}H (400 MHz, $CDCl_3$), compound **7l**

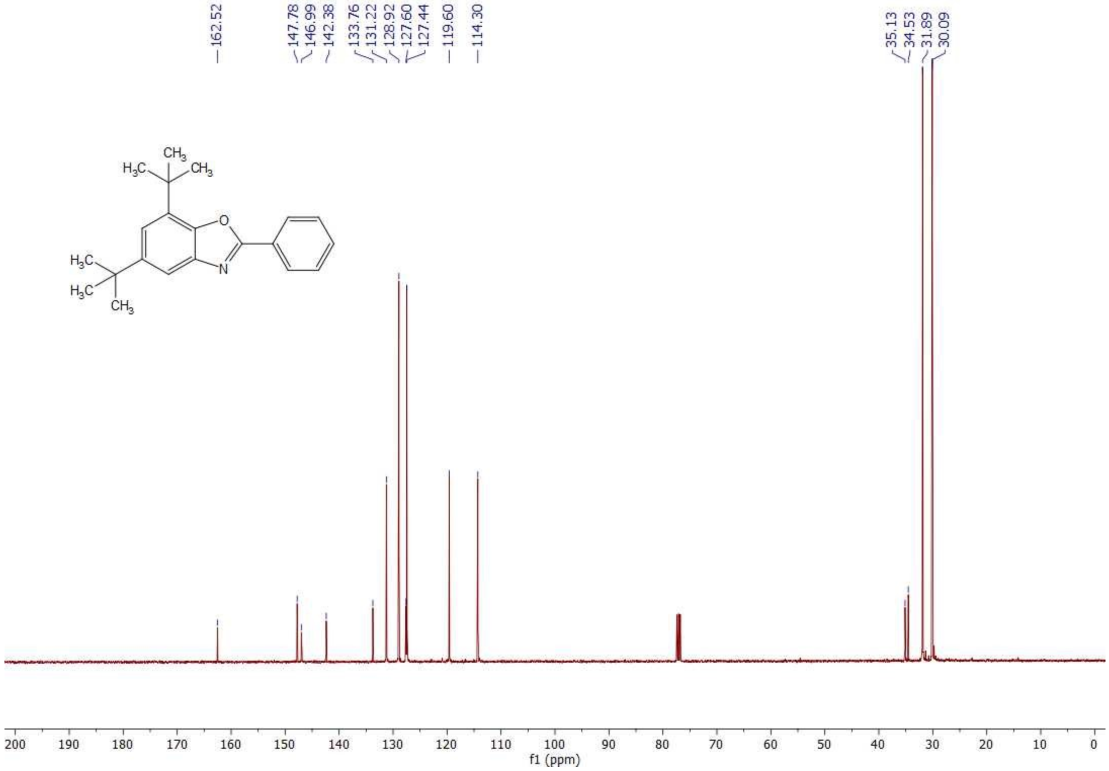

Figure S70. ^{13}C (100 MHz, $CDCl_3$), compound **7l**

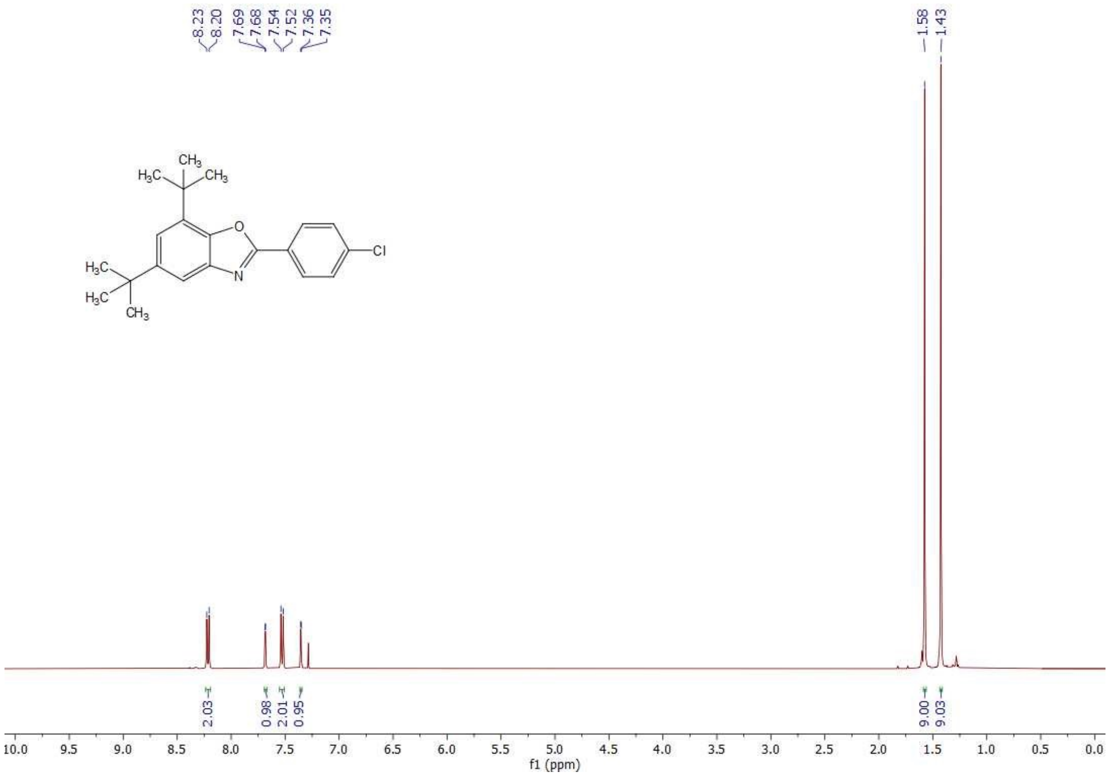

Figure S71. ^{1}H (400 MHz, $CDCl_3$), compound **7m**

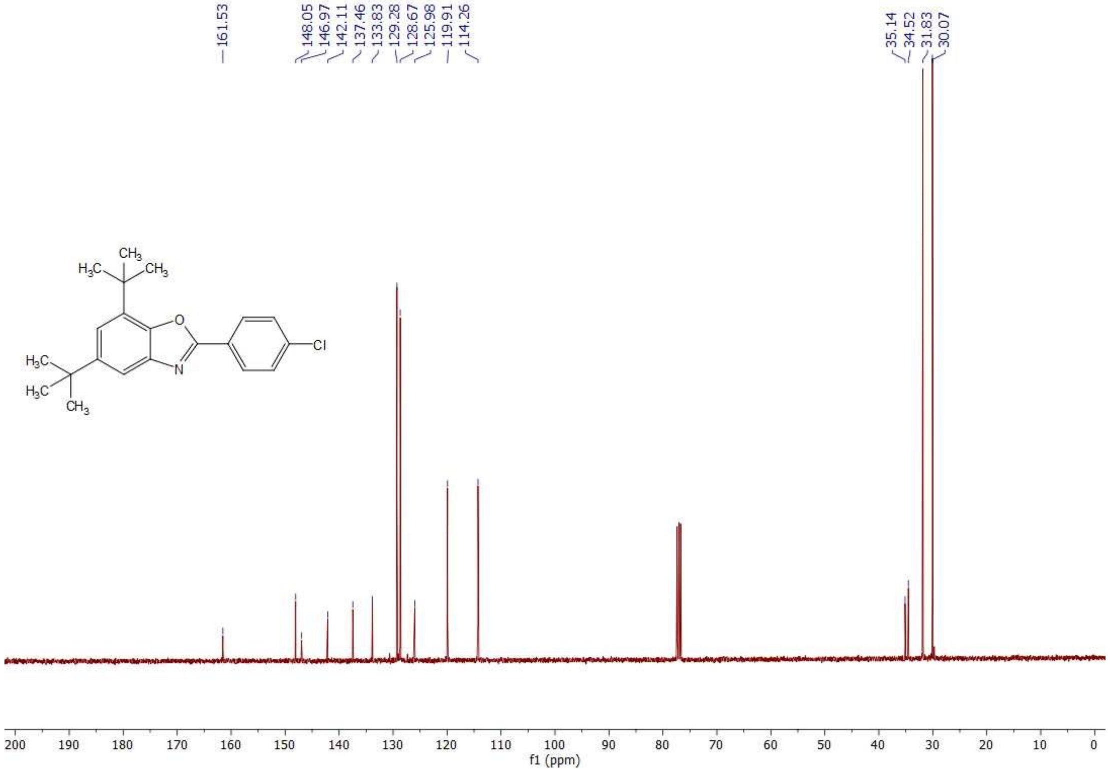

Figure S72. ^{13}C (100 MHz, $CDCl_3$), compound **7m**

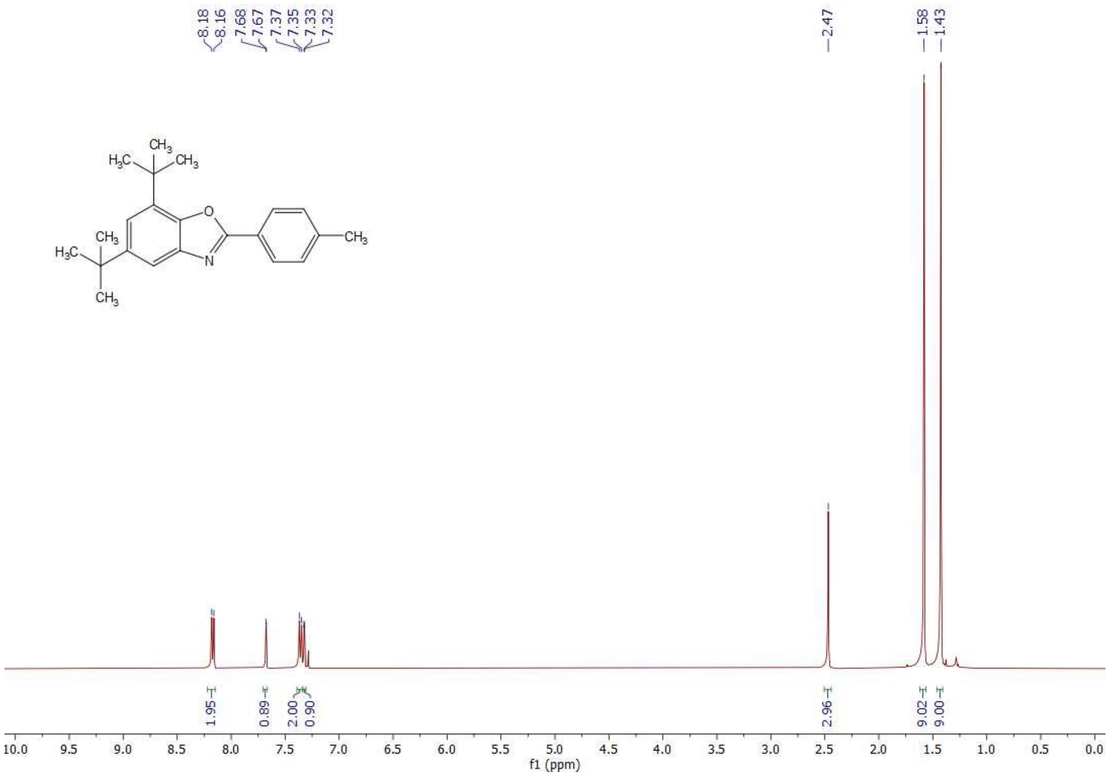

Figure S73. ^{1}H (400 MHz, $CDCl_3$), compound **7n**

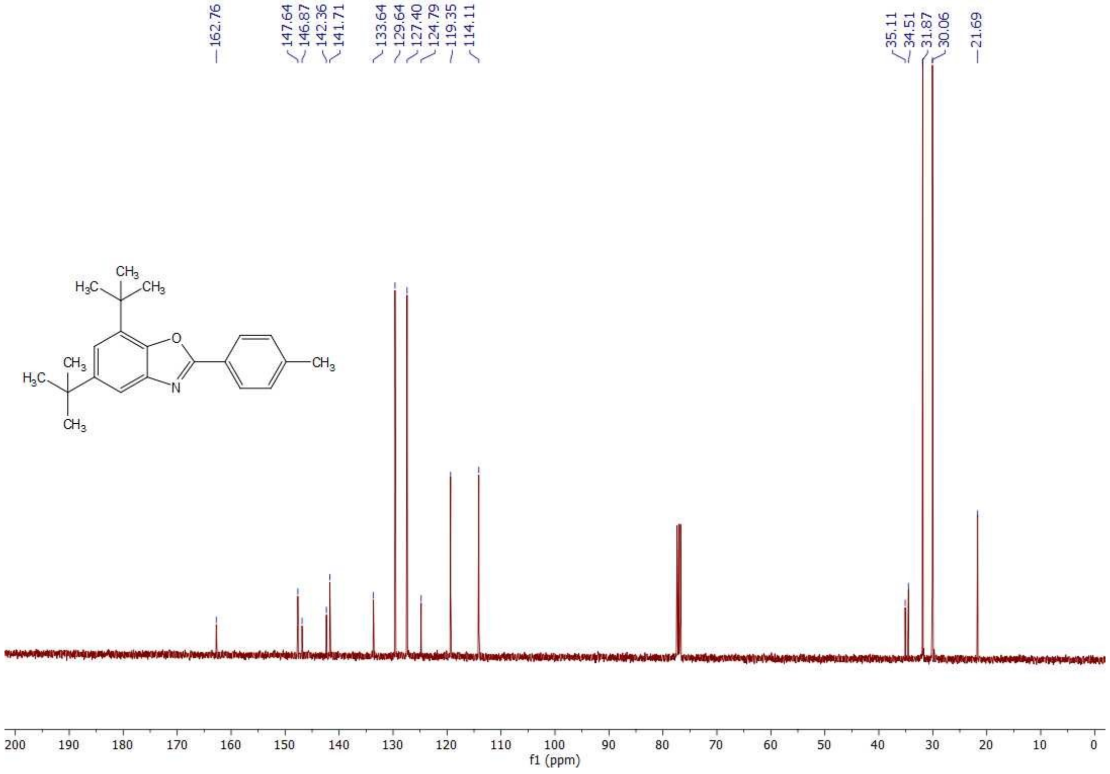

Figure S74. ^{13}C (100 MHz, $CDCl_3$), compound **7n**

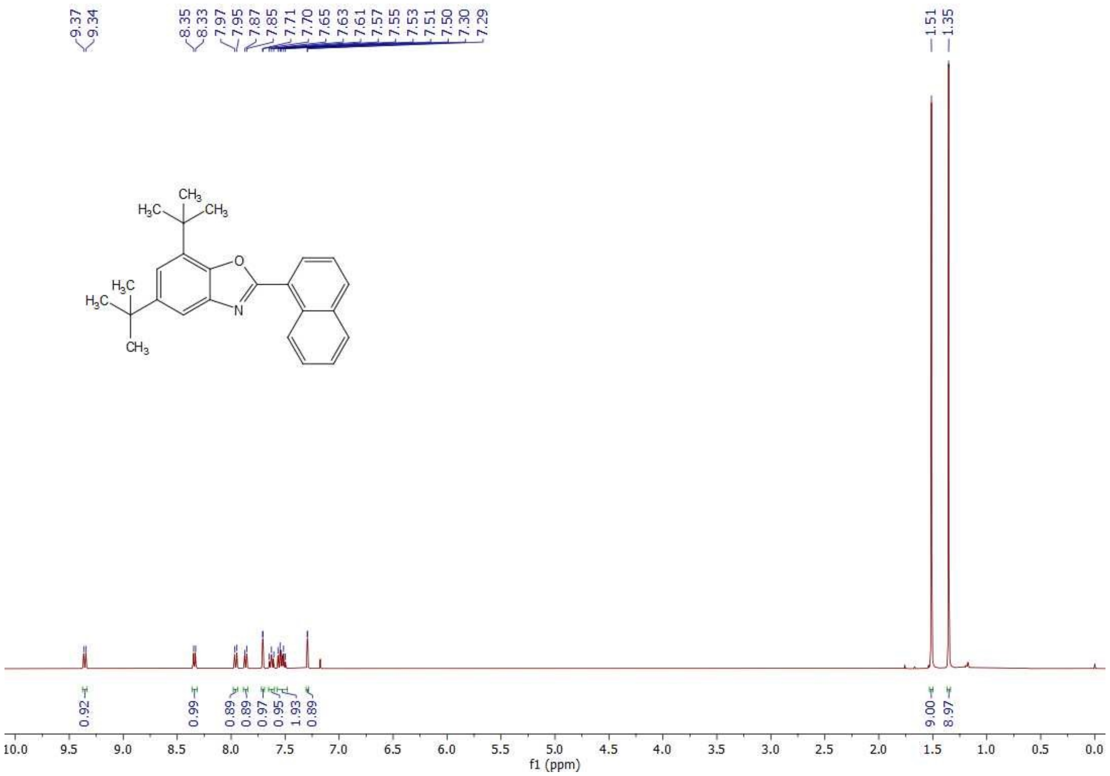

Figure S75. ^{1}H (400 MHz, $CDCl_3$), compound **7o**

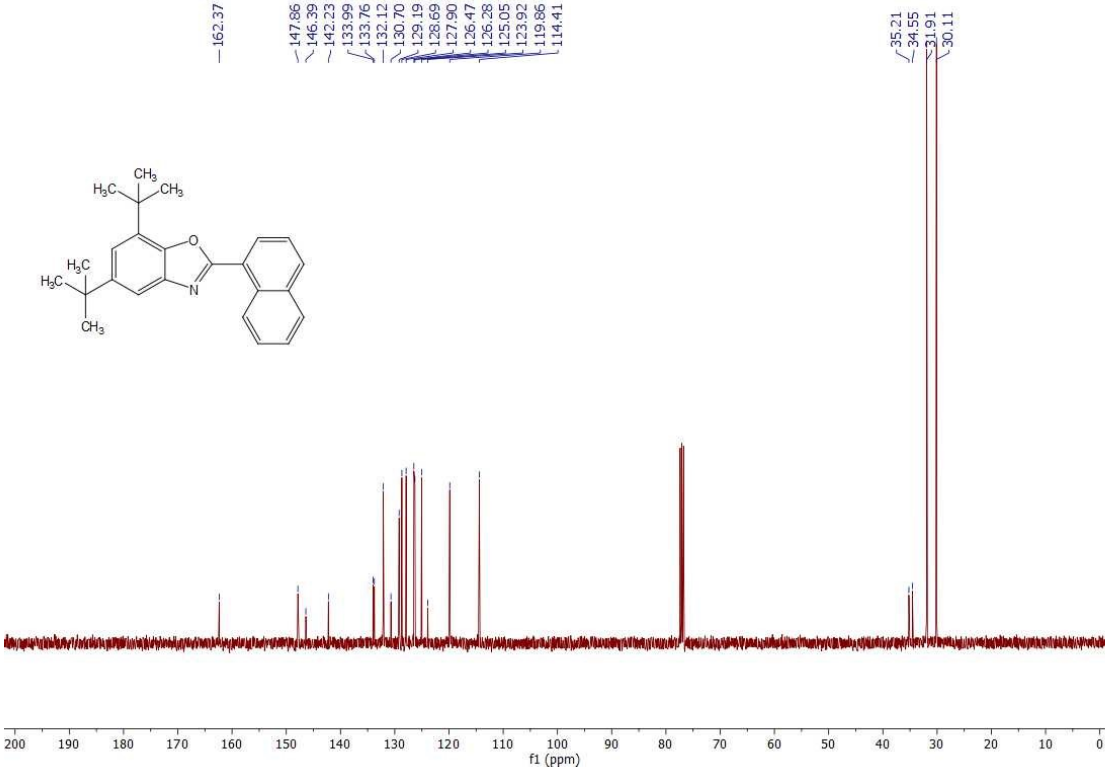

Figure S76. ^{13}C (100 MHz, $CDCl_3$), compound **7o**

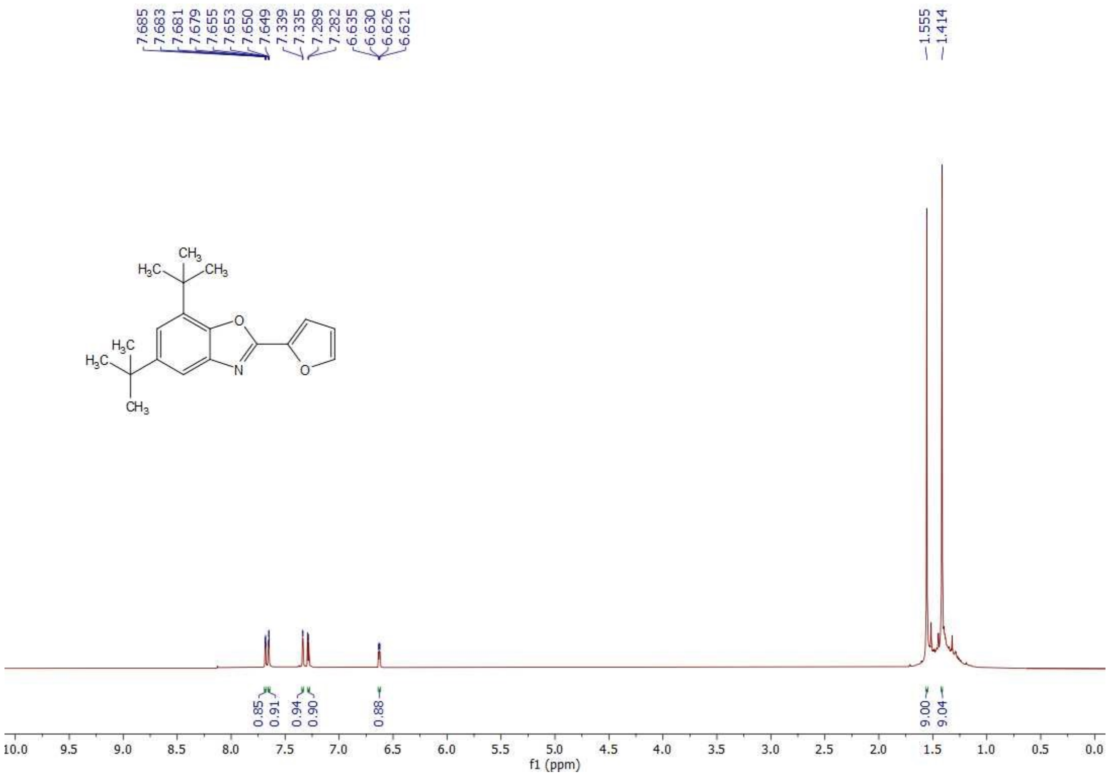

Figure S77. ^{1}H (400 MHz, $CDCl_3$), compound **7p**

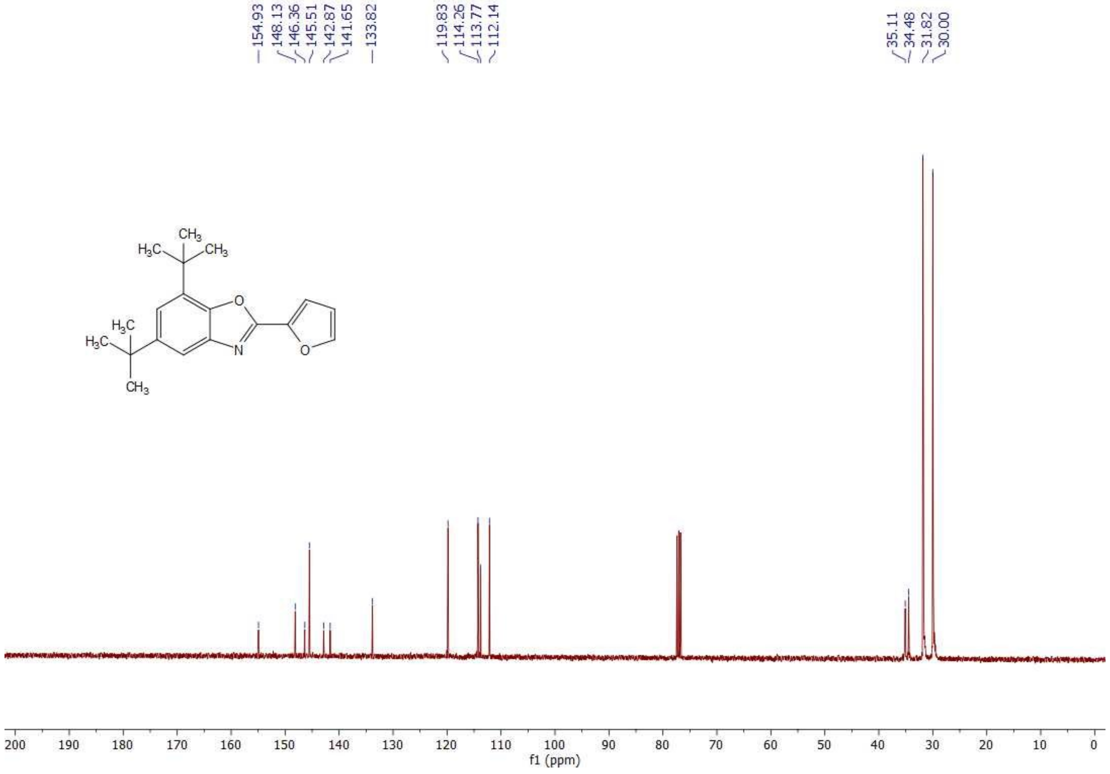

Figure S78. ^{13}C (100 MHz, $CDCl_3$), compound **7p**

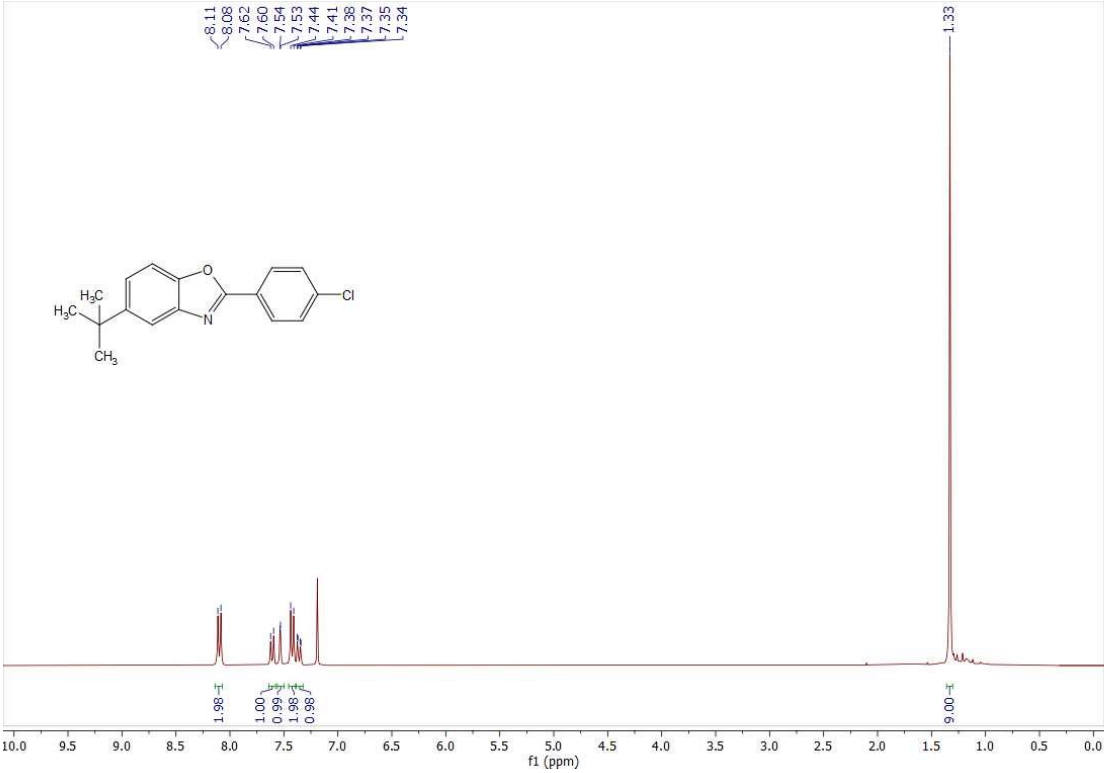

Figure S79. ^{1}H (300 MHz, $CDCl_3$), compound **7q**

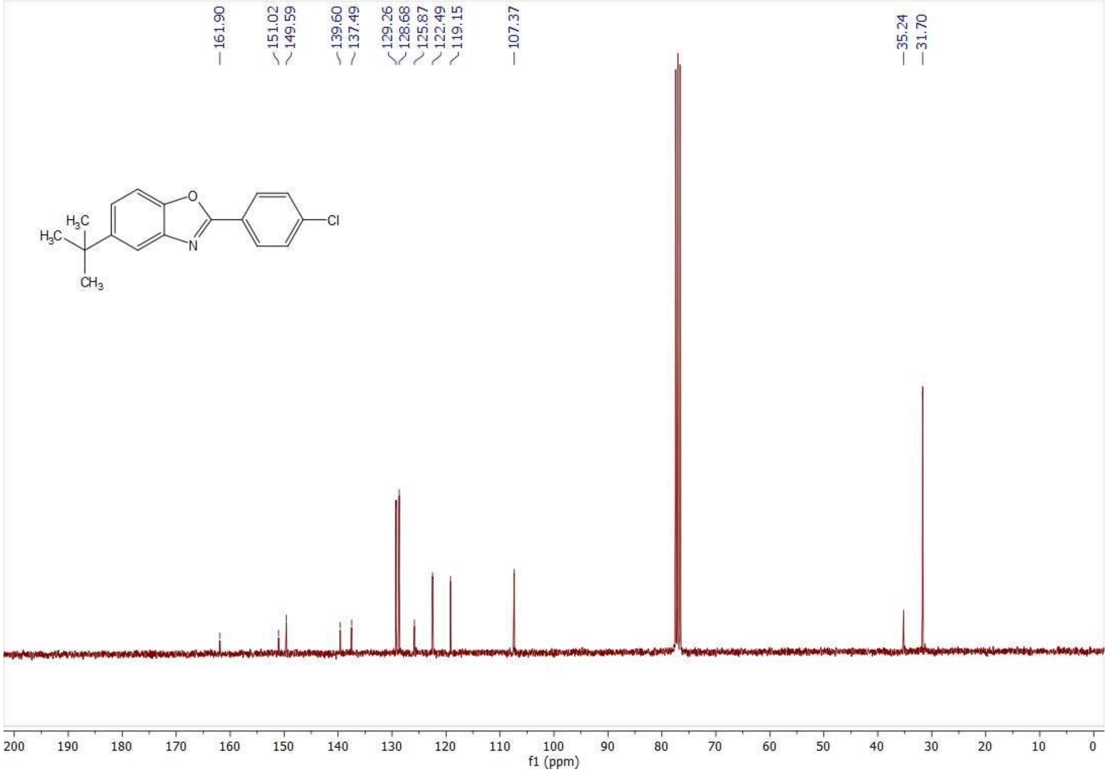

Figure S80. ^{13}C (75 MHz, $CDCl_3$), compound **7q**

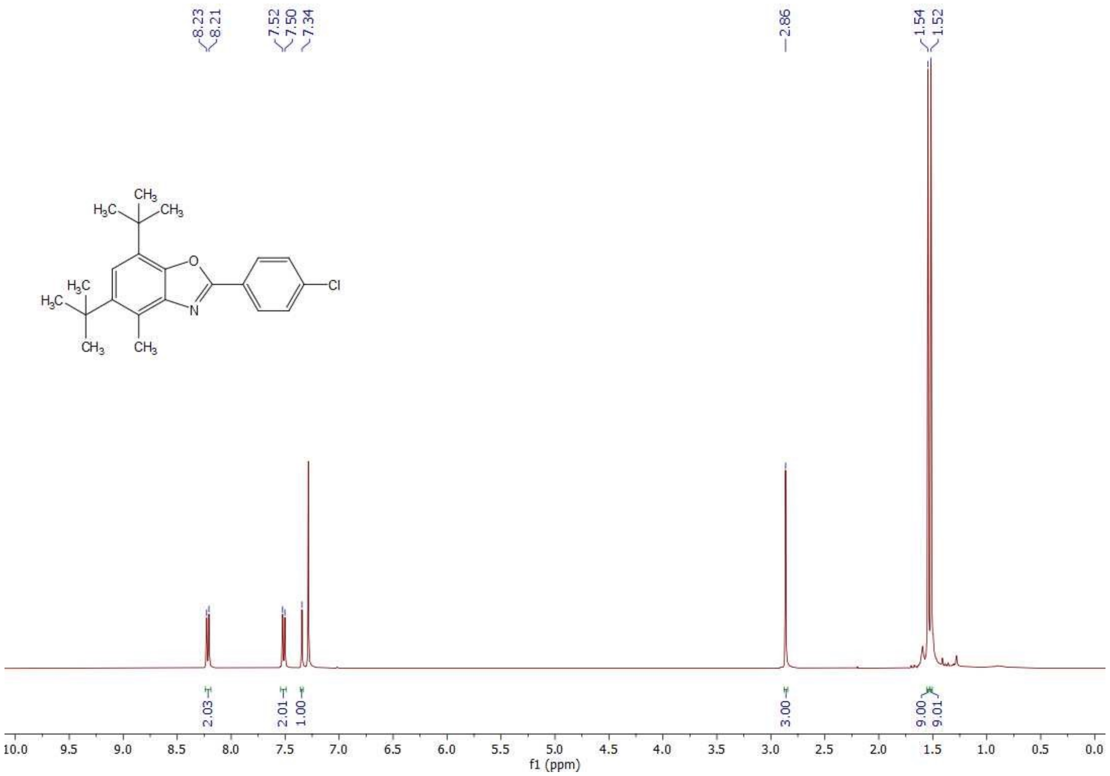

Figure S81. ^{1}H (400 MHz, $CDCl_3$), compound **7r**

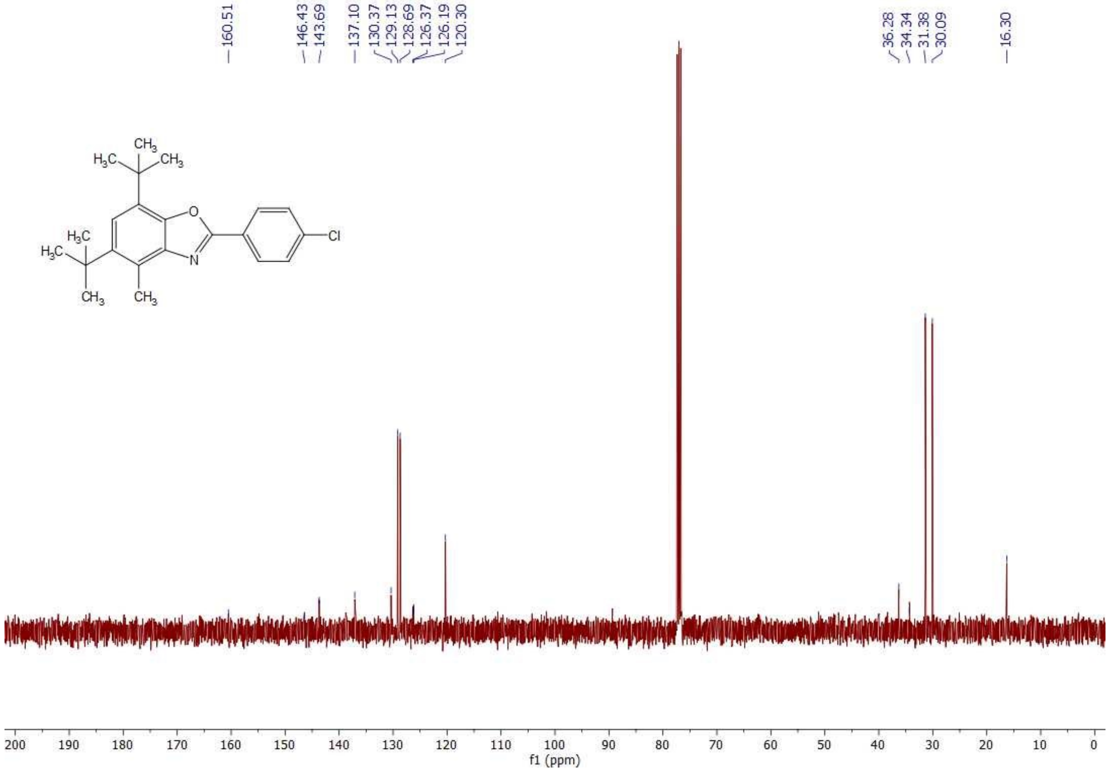

Figure S82. ^{13}C (100 MHz, $CDCl_3$), compound **7r**

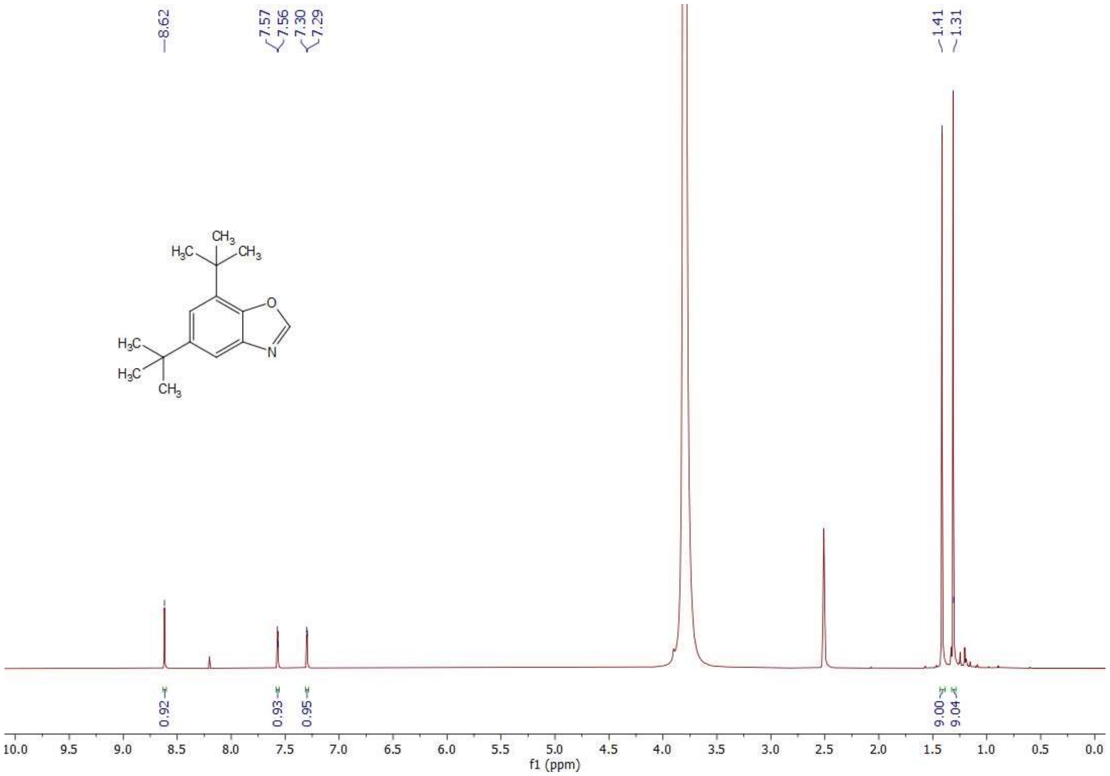

Figure S83. ^{1}H (400 MHz, DMSO-d_6), compound **7s** (*Synthesized from glycine*)

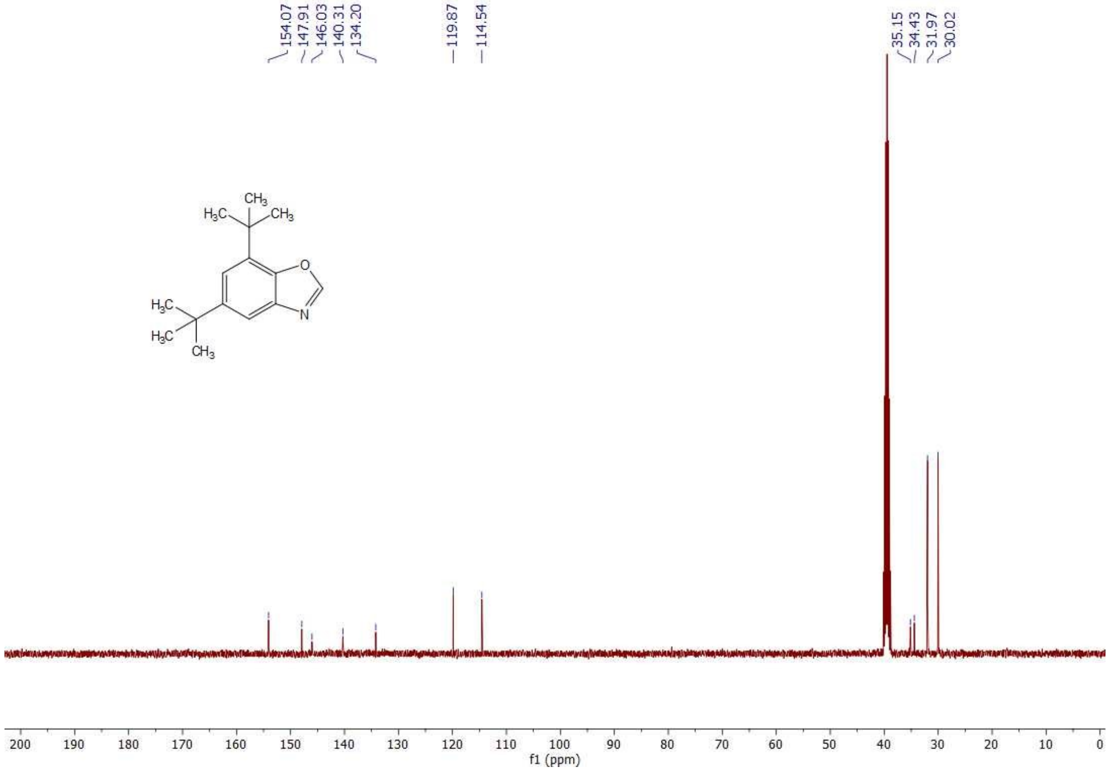

Figure S84. ^{13}C (100 MHz, DMSO-d_6), compound **7s** (*Synthesized from glycine*)

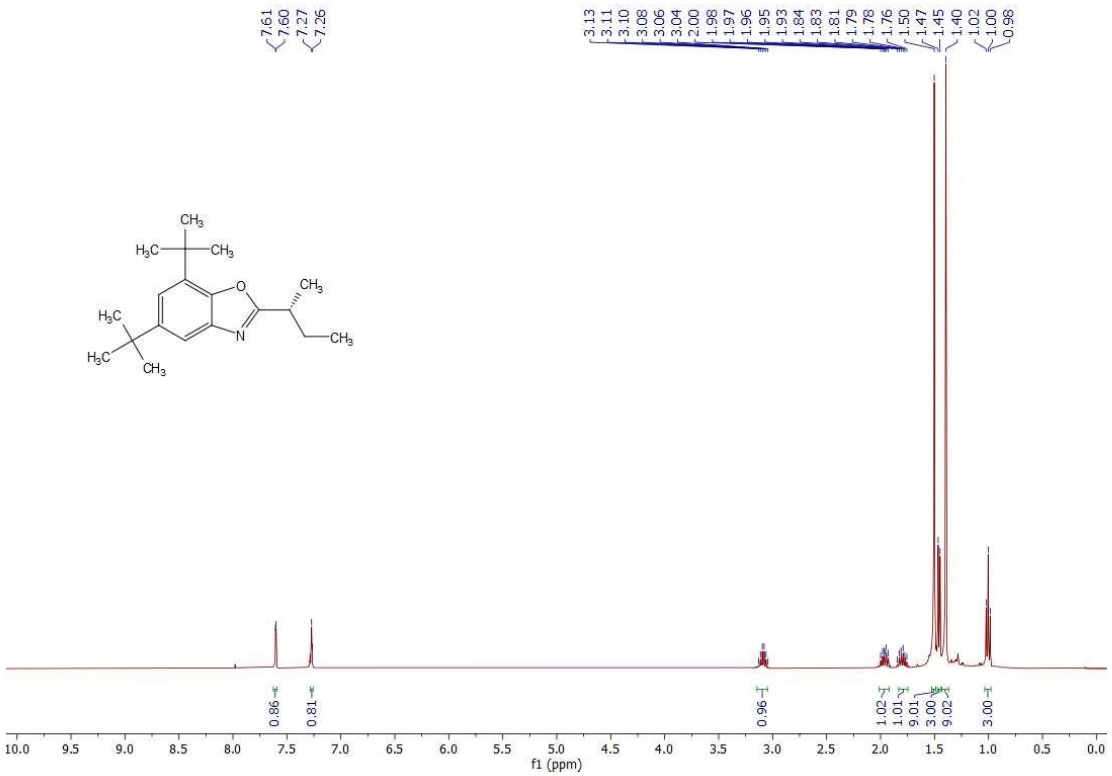

Figure S85. ^{1}H (400 MHz, $CDCl_3$), compound **7t** (*Synthesized from L-isoleucine*)

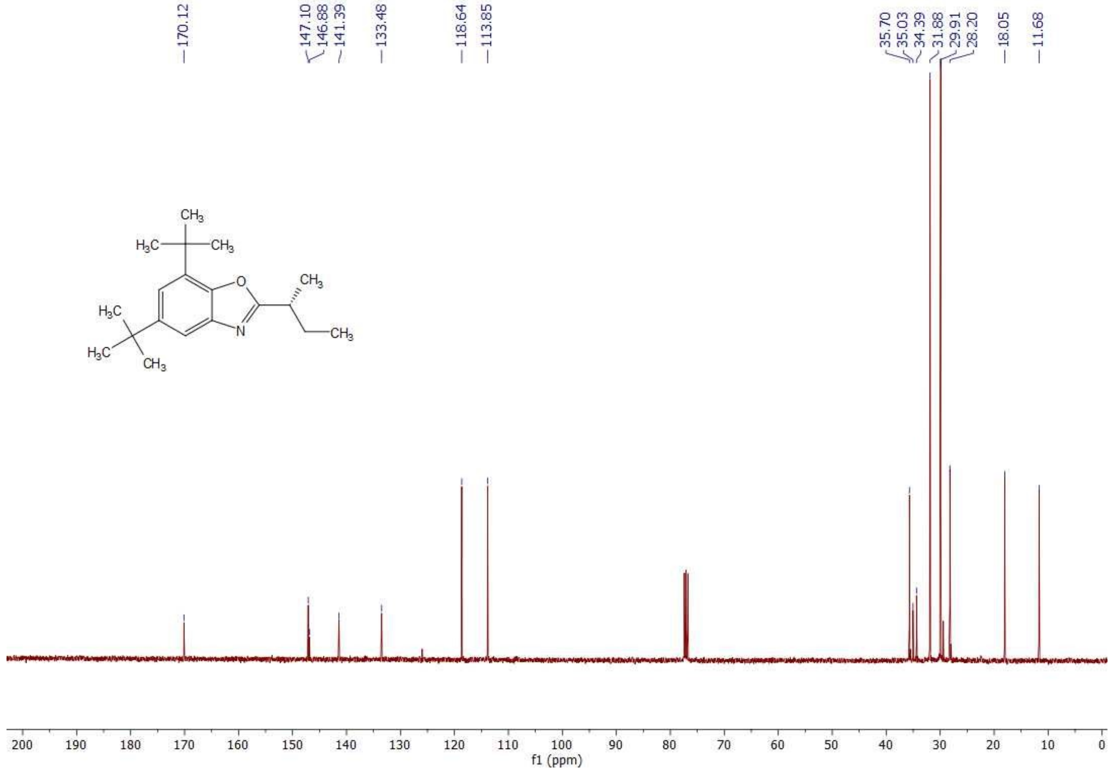

Figure S86. ^{13}C (100 MHz, $CDCl_3$), compound **7t** (*Synthesized from L-isoleucine*)

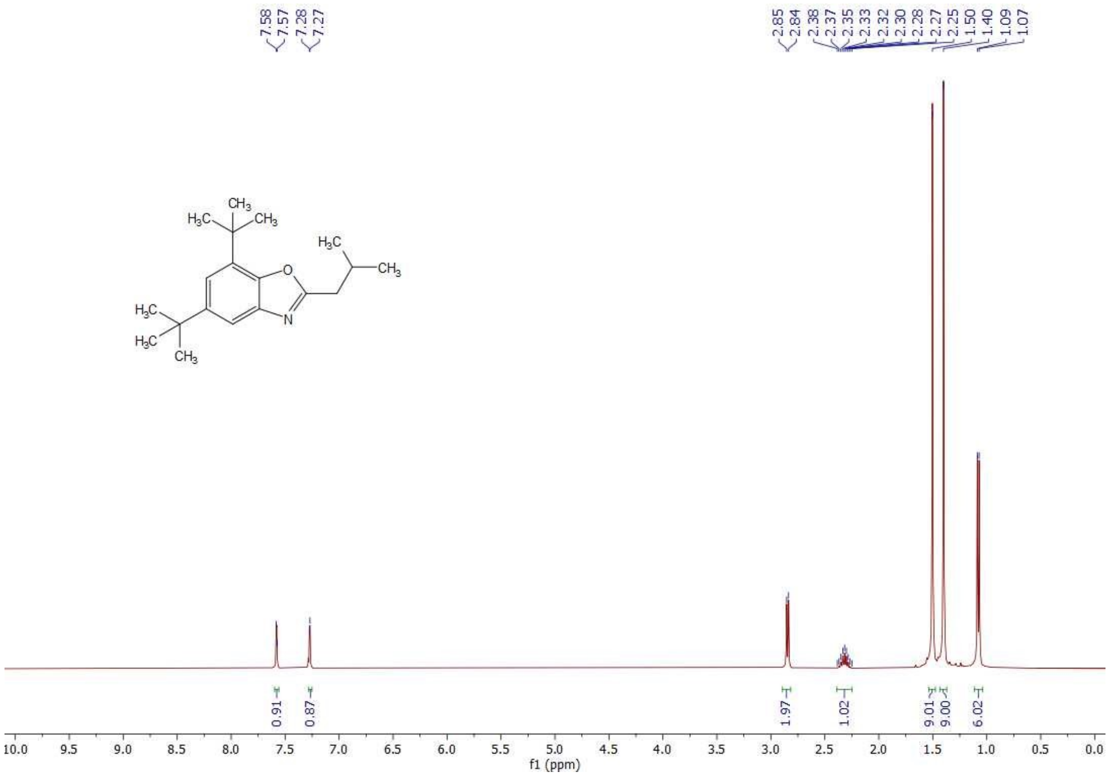

Figure S87. ^{1}H (400 MHz, $CDCl_3$), compound **7u** (*Synthesized from L-leucine*)

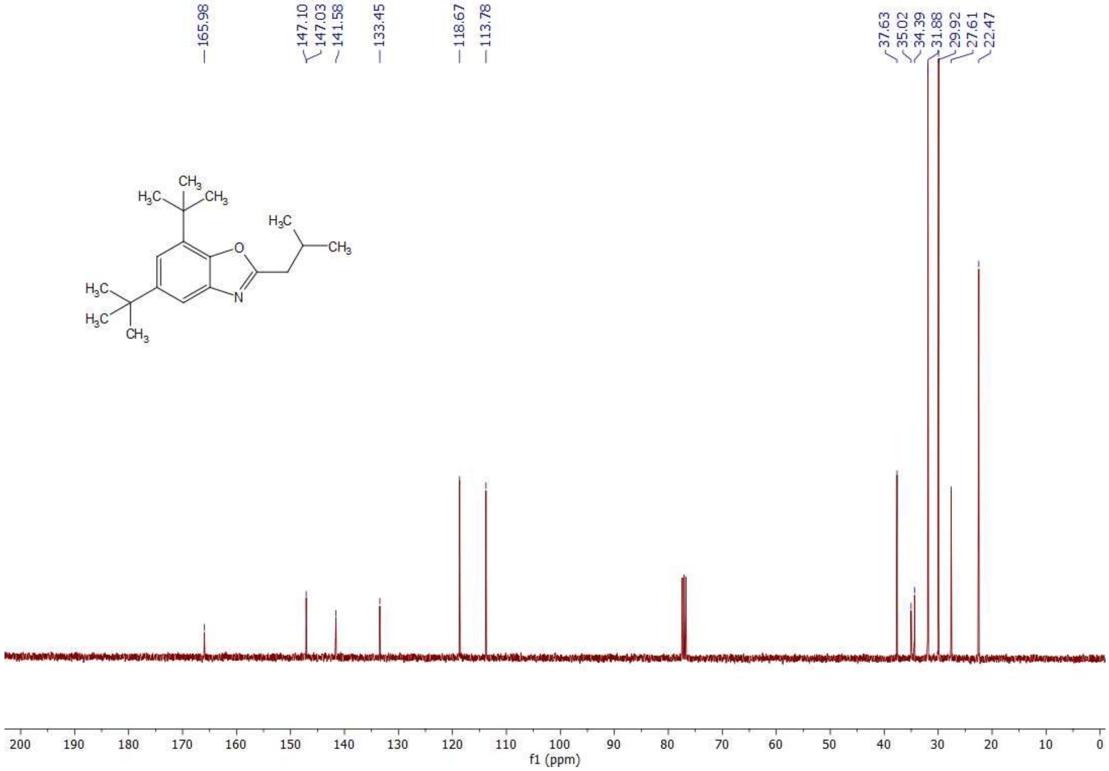

Figure S88. ^{13}C (100 MHz, $CDCl_3$), compound **7u** (*Synthesized from L-leucine*)

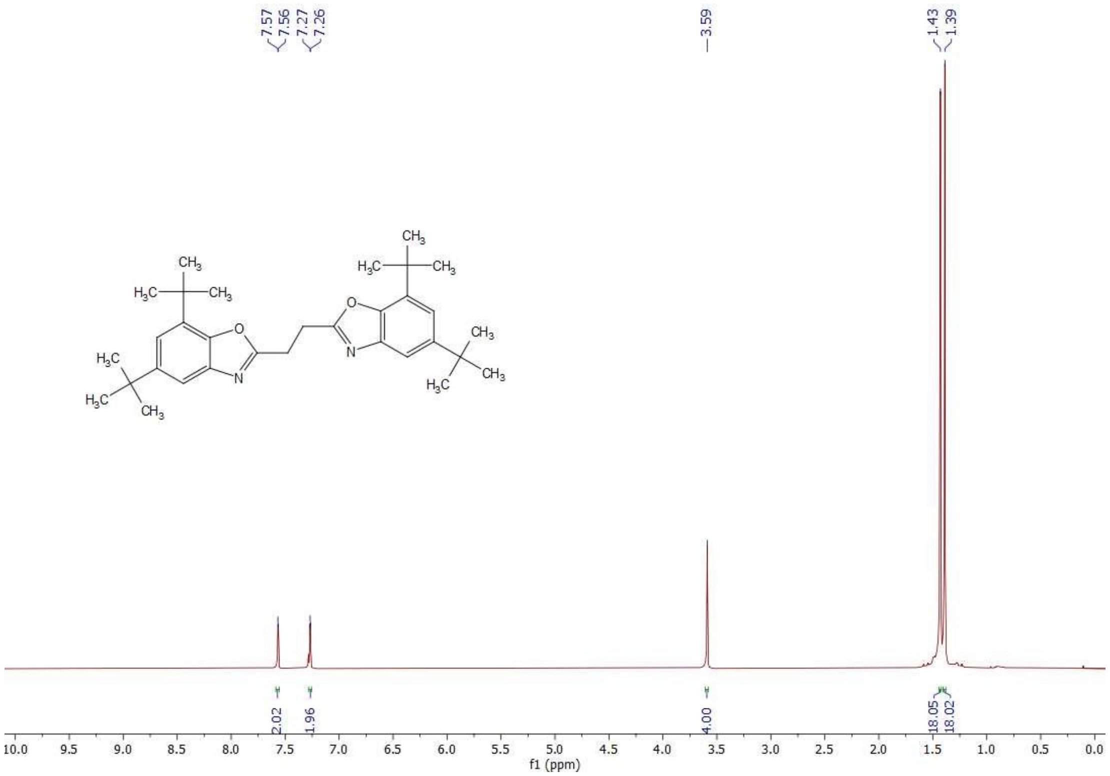

Figure S89. ^{1}H (400 MHz, $CDCl_3$), compound **7v** (*Synthesized from L-arginine*)

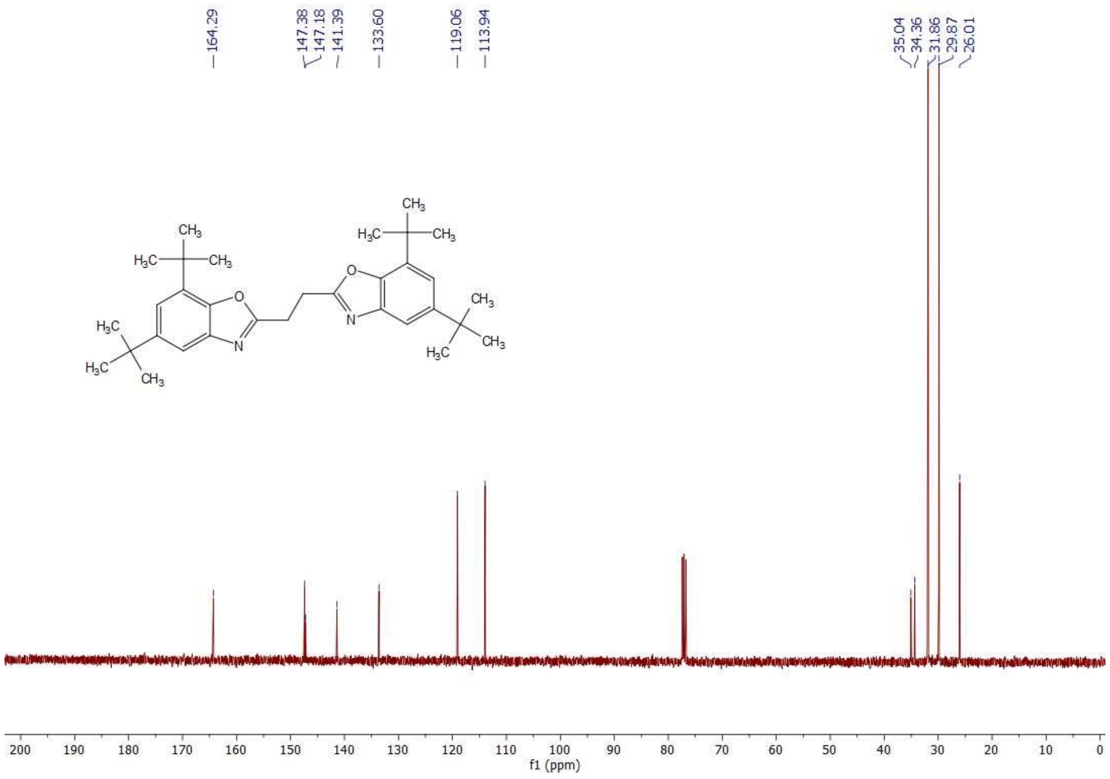

Figure S90. ^{13}C (100 MHz, $CDCl_3$), compound **7v** (*Synthesized from L-arginine*)
